# Broadband transient full-Stokes luminescence spectroscopy

**DOI:** 10.1038/s41586-025-09197-3

**Published:** 2025-06-25

**Authors:** Antti-Pekka M. Reponen, Marcel Mattes, Zachary A. VanOrman, Lilian Estaque, Grégory Pieters, Sascha Feldmann

**Affiliations:** 1 Rowland Institute, Harvard University, Cambridge, MA USA; 2 Institute of Chemical Sciences and Engineering, École Polytechnique Fédérale de Lausanne, Lausanne, Switzerland; 3 Département Médicaments et Technologies pour la Santé, Université Paris-Saclay, CEA, INRAE, Gif-sur-Yvette, France

**Keywords:** Techniques and instrumentation, Circular dichroism, Optical spectroscopy

## Abstract

Materials emitting circularly polarized light (CPL) are highly sought after for applications ranging from efficient displays to quantum information technologies^1–7^. However, established methods for time-resolved CPL (TRCPL) characterization have notable limitations^8–17^, generally requiring a compromise between sensitivity, accessible timescales and spectral information. This has limited the acquisition of in-depth photophysical insight necessary for materials development. Here we demonstrate a high-sensitivity (noise level of the order of 10^−4^), broadband (about 400–900 nm), transient (nanosecond resolution, millisecond range) full-Stokes (CPL and linear polarizations) spectroscopy setup. The achieved combination of high-sensitivity, broad wavelength response and flexible time ranges represents a substantial advancement over previous TRCPL approaches. As a result, TRCPL measurements are shown to be applicable to hitherto inaccessible material systems and photophysical processes, including systems with low (10^−3^) dissymmetry factors and luminescence pathways spanning nanosecond to millisecond time ranges. Finally, full-Stokes measurements allow tracking the temporal evolution of linear polarization components, of interest by themselves, but especially relevant in the context of controlling for associated CPL artefacts^18,19^ in the time domain.

## Abstract (web summary)

A high-sensitivity, broadband, transient, full-Stokes spectroscopy setup is demonstrated, which can detect quickly varying small signals from chiral emitters.

## Main

Materials emitting circularly polarized luminescence have seen a resurgence in research interest over recent years. This is largely driven by emerging and commercially promising technologies using CPL across a broad range in photonics, spin electronics and optoelectronics^20,21^, including photonic and quantum computing^1,2^, security inks^3^, biomedical imaging^4^, efficient device displays^5^ and holographic^6^ and three-dimensional display^7^ technologies. Accordingly, there is marked interest in the accurate characterization of polarized light emission processes.

Earliest CPL measurements trace back to the mid-twentieth century^22,23^, and in the following decades, high-sensitivity CPL instruments were developed. Using a photoelastic modulator (PEM) for rapid polarization modulation^24,25^ followed by lock-in amplification (LIA) or differential photon counting, relative intensity differences of the order of 10^−5^ are measurable. These basic detection principles continue to underlie most high-sensitivity CPL measurements today. CPL is often characterized by the dissymmetry factor, defined as

$${g}_{{\rm{lum}}}=\frac{\Delta {I}_{{\rm{LCP}}/{\rm{RCP}}}}{{I}_{{\rm{avg}}}}=\frac{{I}_{{\rm{LCP}}}-{I}_{{\rm{RCP}}}}{\frac{1}{2}({I}_{{\rm{LCP}}}+{I}_{{\rm{RCP}}})}$$

where *I*_LCP_ and *I*_RCP_ refer to the intensity of LCP (left-handed circularly polarized) and RCP (right-handed circularly polarized) light, respectively.

Although time-resolved measurements are ubiquitous in the characterization of luminescent materials, time-resolved CPL (TRCPL) measurements remain rare. This is despite several TRCPL instruments using PEMs reported in the 1990s (refs. ^8–12^) alongside applications in revealing racemization and energy transfer dynamics^26,27^. In particular, a previous study^13^ used time-correlated single photon counting (TCSPC) and differential photon counting for nanosecond (ns) time resolution and measurement of dissymmetry at the 10^−3^ level in 1995.

In practice, combining PEMs with time resolution introduces limitations. The modulation rate must be compatible with the detector readout rate, excitation repetition rate and the luminescence decay timescale. As the detection scheme is monochromatic, even steady-state broad-spectrum acquisition is time-consuming^18^. With TCSPC additionally dividing signal into time bins and limiting detector count rates to about 1–5% of the excitation rate to avoid pile-up, collecting time-resolved CPL spectra becomes impractical (more discussion in Supplementary Information section 4.3). We note that ref. ^13^ discusses long measurement times in the context of TCSPC-based TRCPL despite MHz excitation rates and a simple filter to select the emission range, already excluding long-lived luminescence and detailed spectra^13^.

These limitations may be why the few contemporary works in TRCPL^14–17^ rely on quarter-wave plates (QWP) instead of modulation, resulting in simpler instruments and faster acquisition. However, the lack of modulation results in low CPL sensitivity. These approaches are, therefore, mostly limited to materials such as chiral lanthanide complexes with extreme dissymmetries^28^.

However, lanthanide complexes are only a fraction of the diverse material platforms explored at present for CPL applications, many of which feature short-lived (ns) luminescence and/or small dissymmetry factors (around 10^−3^) (refs. ^29,30^). Hence, to fuel these developments, TRCPL methods are required that combine sensitive broadband acquisition with the flexibility to probe both fluorescence (ns) and phosphorescence (µs–ms) on their respective timescales.

## Measuring principle

**Fig. 1:**
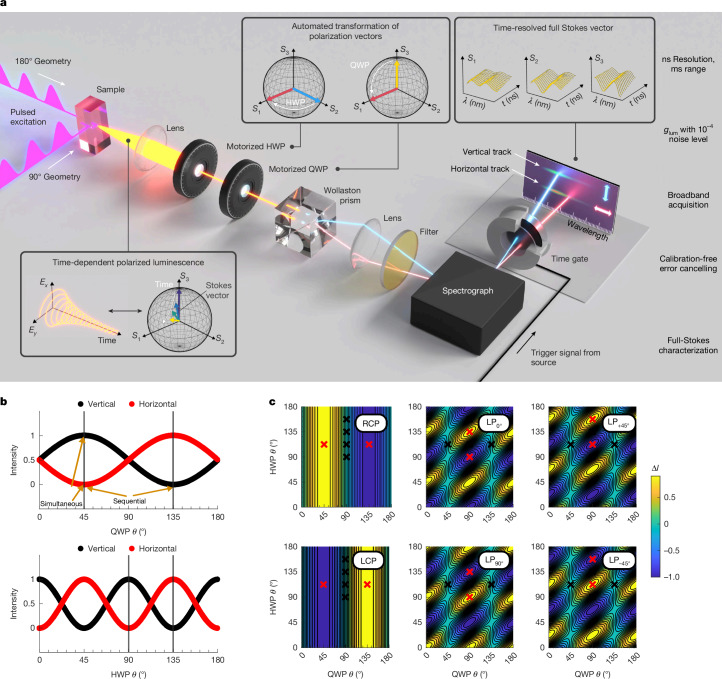
Overview of key setup components, features and principles. **a**, Schematic of the key optical components and measurement procedure. Excitation by a pulsed laser (200 fs, variable repetition rate 0.5–50 kHz) in the 90° geometry eliminates certain polarization artefacts, whereas the 180° geometry is more suitable for thin samples. Superachromatic waveplates (HWP for half-wave plate, QWP for quarter-wave plate) in appropriate orientations transform polarization components into orthogonal linear components, which are separated by a Wollaston prism and passed through a grating spectrograph. Orthogonally polarized spectra are recorded simultaneously as two tracks on a single CCD array detector. An intensifier tube is electronically gated to provide time resolution. Waveplate rotation, automatable by motorized housings, allows swapping the detection tracks, which results in the cancellation of the largest channel transmission mismatches and temporal instabilities. **b**, Calculated intensities at vertical and horizontal detection tracks as a function of QWP angle for an RCP luminescence input and HWP angle for a linearly polarized input. Two tracks are simultaneously recorded, and the measurement is repeated at a second QWP/HWP position at which the tracks swap. **c**, Calculated intensity difference between measurement tracks as a function of both QWP and HWP angle for the pure Stokes basis polarizations. Red crosses indicate angles where measurements are taken for that polarization component. For reference, black crosses (each of which is at Δ*I* = 0) indicate angles where measurements are taken for other polarization components. Source Data

Our approach to TRCPL is shown in Fig. 1. An electronically gated charge-coupled device (CCD) camera allows flexible time gates from 2 ns to several milliseconds (ms), is inherently broadband, and is not limited by photon pile-up effects. To achieve high sensitivity without polarization modulation, we simultaneously record orthogonal linear polarizations on different parts of the CCD array (2,048 × 512 pixels), as recently applied in ref. ^31^ for calibration-free error cancelling in a steady-state CPL spectrometer and previously used in fluorescence correlation time imaging^32^. The reader is referred to these works for an excellent coverage of the theory and associated errors. Error cancelling enables sensitive measurements of dissymmetry, with noise floors of the order of 10^−4^ for sufficiently bright luminescence. Using a single CCD further eliminates issues with channel synchronicity and reduces overall cost and complexity compared with dual-detector approaches^17^.

Polarization components are separated by a series of polarization optics consisting of a half-wave plate (HWP), QWP and a Wollaston prism. Repeating measurements for a series of waveplate angles allows for cancellation of various error sources^31^ and isolation of specific polarization components. Waveplate interactions with different polarization states are shown in Fig. 1c. At suitable angle combinations, only a specific component of the Stokes vector is translated to the intensity difference between channels. Accordingly, linear polarization components in luminescence (a primary error source in CPL measurements^18^) can be quantified apart from CPL. This results in a sensitive, time-resolved broadband measurement of the full Stokes vector. More detail is given in the Methods and Supplementary Information sections 1, 2 and 4.

We note that the relative angle and polarization of the excitation beam with respect to the collection optics (common configurations shown in Fig. 1a) result in varying photoselection effects^19^ and can affect the polarization of collected luminescence (Methods and Supplementary Information section 4.1). These effects can be especially relevant in time-resolved studies, if relaxation of dipole orientations occurs slower than the measurement time resolution^33^. Besides photoluminescence, circularly polarized electroluminescence (CP-EL), for example, from a spin-LED, could equally be characterized instead.

For the remainder of this work, we showcase setup performance with data collected on small molecules in solution with varying degrees (strong, weak and none) of CPL dissymmetry and excited-state timescales from single-ns to >100 μs.

## Chiral lanthanide complex

### Steady-state and microsecond gating of Eu[(+)-facam]_3_ luminescence

**Fig. 2:**
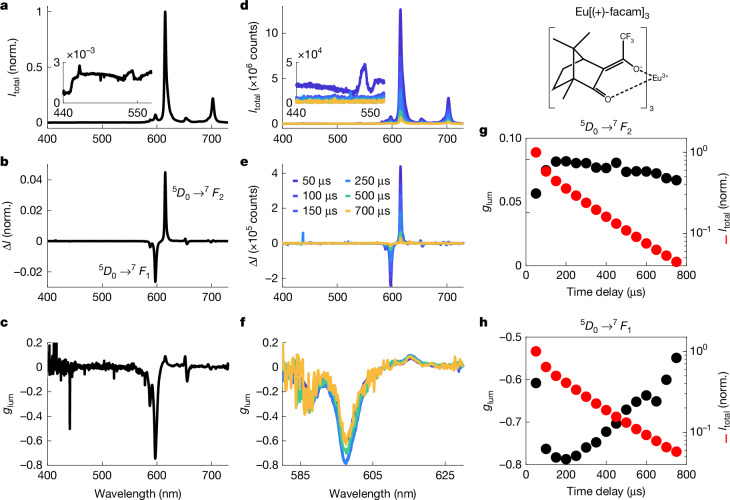
Steady-state and microsecond time-resolved CPL spectroscopy of the chiral standard Eu[(+)-facam]_3_ in DMSO solution. Structure of the chiral standard Eu[(+)-facam]_3_ is shown at the top right. **a**, Total steady-state luminescence intensity (405 nm continuous wave excitation, 450 nm long-pass filter). The inset shows weak emission on the blue side, partially cut off by the filter. **b**, Steady-state CPL spectrum showing the intensity difference *∆I* between LCP and RCP luminescence, normalized to the maximum total luminescence intensity. The main CPL features are labelled, ^5^*D*_0_ → ^7^*F*_1_ near 595 nm and ^5^*D*_0_ → ^7^*F*_2_ near 615 nm. **c**, Steady-state spectrum of the luminescence dissymmetry factor *g*_lum_. **d**, Time-resolved total luminescence spectra (343 nm excitation pulsed at 500 Hz, 50 µs time bins) at selected gate delays. **e**, Time-resolved CPL spectra at selected gate delays. **f**, Time-resolved spectra of the luminescence dissymmetry factor at selected gate delays. **g**, Intensity and dissymmetry factors as a function of time at the ^5^*D*_0_ → ^7^*F*_2_ peak. **h**, Intensity and dissymmetry factors as a function of time at the ^5^*D*_0_ → ^7^*F*_1_ peak (small upticks at 450 μs in **g** and 650 μs in **h** are probably because of cosmic rays). Source Data

Lanthanide complexes with chiral ligands often show strong CPL^28,34^. In particular, Eu[(+)-facam]_3_ ((+)-facam = 3-(trifluoromethylhydroxymethylene)-(+)-camphorate; structure shown in Fig. 2) is commercially available and possesses high emission dissymmetry (*g*_lum_ reaching −0.78 in dry DMSO at 595 nm) (ref. ^24^). As such, Eu[(+)-facam]_3_ is a common standard for CPL setup validation and testing for which multiple literature sources exist for comparison^18,24^. These reports include a recent CCD-based CPL setup with time gating functionality^17^ and fully time-resolved studies in ref. ^16^, which shows the temporal evolution of dissymmetry with approximately 10 μs resolution and millisecond range.

To establish comparison with existing literature, we first present steady-state and long timescale time-resolved (50 μs gate width) CPL spectra of Eu[(+)-facam]_3_ in dry DMSO (Fig. 2). The sharp emission features in this complex arise from *f*–*f* transitions of the Eu^3+^ centre, which generally are of the form $\genfrac{}{}{0ex}{}{\,5}{}{D}_{{J}_{1}}\,\to \,\genfrac{}{}{0ex}{}{7}{}{F}_{{J}_{2}}$ (where *J*_1_, *J*_2_ are the total angular momentum quantum numbers) and can be assigned to $\genfrac{}{}{0ex}{}{5}{}{D}_{0}\,\to \,\genfrac{}{}{0ex}{}{7}{}\,{F}_{{J}_{2}}$ transitions specifically^35–37^. Of these, the most striking CPL features appear at 595 nm and 613 nm, corresponding to a ^5^*D*_0_ → ^7^*F*_1_ magnetic dipole transition and a ^5^*D*_0_ → ^7^*F*_2_ induced electric dipole transition, with reported *g*_lum_ values of –0.78 and +0.072, respectively^16,18,24^ (although with some variance and environmental sensitivity^38^, notably to water^16^).

In the steady state, our luminescence and CPL spectra match literature (Fig. 2a–c), although the recorded *g*_lum_ of −0.745 for the ^5^*D*_0_ → ^7^*F*_1_ transition is slightly lower than expected. Besides potential issues with, for example, water ingress, a possible explanation is the relatively coarse grating necessary for broadband acquisition and finite detector pixel size resulting in a wavelength resolution limit of about 1 nm, similar to the line width of the feature (see Supplementary Fig. 19 for effects of water contamination and Supplementary Fig. 10 for the effects of slit width on measured *g*_lum_).

Although the time-resolved luminescence spectra (Fig. 2d–f) show little evolution of the spectral shape over time, the magnitude of *g*_lum_ changes (Fig. 2g,h). The reduction in *g*_lum_ over time is similar to what was observed in ref. ^16^, in which a decrease from a peak value of approximately −0.8 with a time constant of approximately 1 ms is reported and assigned to sample heterogeneity. Moreover, our data show an increase in *g*_lum_ for the first few time bins. This is more apparent for the ^5^*D*_0_ → ^7^*F*_1_ peak (Fig. 2h) than the ^5^*D*_0_ → ^7^*F*_2_ peak (Fig. 2g). The early-time rise in *g*_lum_ is possibly linked to weak transitions seen on the blue side in the earliest time bins (Fig. 2d, inset), which will overlap more with the ^5^*D*_0_ → ^7^*F*_1_ peak at 595 nm than the ^5^*D*_0_ → ^7^*F*_2_ peak at 613 nm. These features are also faintly visible in the steady-state measurement (Fig. 2a, inset), although partially cut off by the filter. These early-time features are investigated in more depth in the next section. A flat or slightly rising *g*_lum_ at early times is also reported in ref. ^16^, although in this study, the earliest time points were not sampled. Overall, both steady-state and μs-timescale time-resolved measurements are consistent with the literature for Eu[(+)-facam]_3_ in DMSO.

### Nanosecond polarization dynamics of Eu[(+)-facam]_3_

**Fig. 3:**
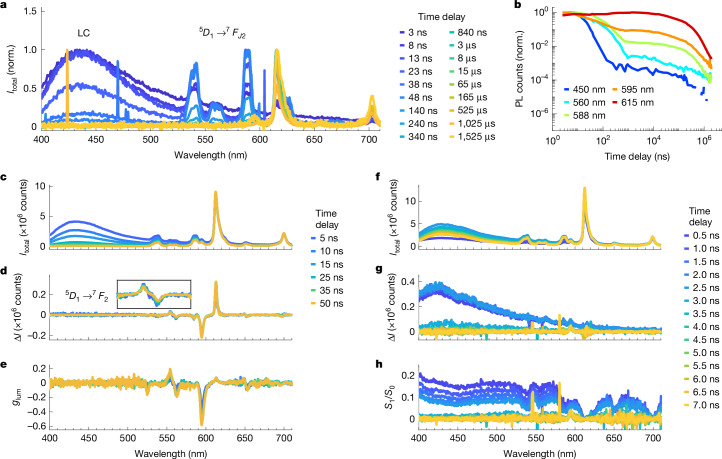
Nanosecond time-resolved non-polarized luminescence, circular anisotropy and photoselection-induced linear anisotropy of Eu[(+)-facam]_3_ in DMSO solution. **a**, Non-polarization sensitive luminescence spectra (excitation 343 nm, 200 fs and 500 Hz) with varying time bins, normalized by maximum value to highlight spectral changes. Key early-time spectral features are labelled as the broad peak around 400–500 nm (LC), and a series of peaks at 520–600 nm ($\genfrac{}{}{0ex}{}{\,5}{}{D}_{1}\,\to \,\genfrac{}{}{0ex}{}{7}{}{F}_{{J}_{2}}$). **b**, Kinetic traces of the non-polarization sensitive luminescence signal, at wavelengths corresponding to key features in spectra. **c**–**e**, Time-resolved CPL spectra (excitation 343 nm, 200 fs and 50 kHz) with 5 ns bins using horizontal excitation polarization to avoid photoselection. The transition ^5^*D*_1_ → ^7^*F*_2_ at 560 nm is shown magnified in an inset and labelled. **f**–**h**, Time-resolved *S*_1_ linear polarization measurement (excitation 343 nm, 200 fs and 50 kHz) with 2 ns bins and 0.5 ns steps, using vertical excitation polarization to intentionally induce photoselection (note that because of the time step and bin width, early-time bins fall within instrument response). Source Data

Having established a comparison with available literature in the steady-state and μs-timescale TRCPL of Eu[(+)-facam]_3_ in DMSO, we turn to TRCPL on single-ns timescales (Fig. 3). To the best of our knowledge, this is the first report of such data.

The time-resolved luminescence spectra (Fig. 3a) show several features at early times apart from the aforementioned $\genfrac{}{}{0ex}{}{5}{}{D}_{0}\,\to \,\genfrac{}{}{0ex}{}{7}{}\,{F}_{{J}_{2}}$ transitions. Most prominent is a broad unstructured peak around 435 nm (exact peak position may be affected by transmission and sensitivity at the blue edge), with a series of narrower peaks in the 520–570 nm range. To assign these early-time Eu[(+)-facam]_3_ features, we briefly discuss the photophysics of europium(III) complexes^35^. The Eu^3+^ ion has several well-studied spectral lines arising from $\genfrac{}{}{0ex}{}{5}{}{D}_{1}\,\to \,\genfrac{}{}{0ex}{}{7}{}\,{F}_{{J}_{2}}$ transitions^37,39^. These transitions are weakly absorbing (being magnetic dipole transitions or Laporte forbidden electric dipole transitions^40,41^), but strongly absorbing ligands with suitable energy levels can act as antennae that sensitize the luminescent *f–f* excited states in the lanthanide ion^42^.

Initially, a ligand-centred state (^1^LC) is excited, which can transfer the energy to the Eu^3+^ ion after intersystem crossing (ISC) to a ^3^LC triplet state^43^. Incomplete ISC or energy transfer can result in LC-state luminescence^44^. As energy transfer occurs preferentially via the ^5^D_1_ level^45^ per the respective selection rules, emission from ^5^D_1_ (and even the higher-energy states ^5^D_2_ and ^5^D_3_) is occasionally observed, typically with a much shorter decay time than the main ^5^D_0_ emission^35^.

The features observed for Eu[(+)-facam]_3_ are consistent with such a mechanism, showing features consistent with LC, $\genfrac{}{}{0ex}{}{5}{}{D}_{1}\,\to \,\genfrac{}{}{0ex}{}{7}{}{F}_{{J}_{2}}$ and $\genfrac{}{}{0ex}{}{5}{}{D}_{0}\,\to \,\genfrac{}{}{0ex}{}{7}{}{F}_{{J}_{2}}$ with progressively longer lifetimes. Kinetics at various wavelengths are collected in Fig. 3b, and multi-exponential fits are collected in Supplementary Fig. 6). Owing to multiple timescales and overlapping features (LC and $\genfrac{}{}{0ex}{}{5}{}{D}_{1}\,\to \,\genfrac{}{}{0ex}{}{7}{}{F}_{{J}_{2}}$ luminescence have different lifetimes, and $\genfrac{}{}{0ex}{}{5}{}{D}_{0}\,\to \,\genfrac{}{}{0ex}{}{7}{}{F}_{{J}_{2}}$ luminescence has previously been reported as biexponential^16^), extracting robust lifetimes is difficult. Estimated timescales are 13 ns for the LC peak and 130 ns for the $\genfrac{}{}{0ex}{}{5}{}{D}_{1}\,\to \,\genfrac{}{}{0ex}{}{7}{}{F}_{{J}_{2}}$ lines. Both are short-lived compared with the $\genfrac{}{}{0ex}{}{5}{}{D}_{0}\,\to \,\genfrac{}{}{0ex}{}{7}{}{F}_{{J}_{2}}$ lines (lifetimes of the order of 100 μs), which dominate steady-state measurements.

To determine the CPL activity of early-time luminescence pathways, we have performed a time-resolved CPL measurement with gate steps and widths of 5 ns (Fig. 3c–e). For minimizing photoselection effects, a horizontal excitation polarization was used. To achieve a sufficient signal-to-noise ratio for CPL, a 50-kHz laser repetition rate was necessary, and, therefore, some roll-over emission is present (data with 50 ns gate steps and 500 Hz repetition rate to avoid roll-over is shown in Supplementary Fig. 8). Both LC and $\genfrac{}{}{0ex}{}{5}{}{D}_{1}\,\to \,\genfrac{}{}{0ex}{}{7}{}{F}_{{J}_{2}}$ luminescence are observed. The ligand-centred transition does not seem to be discernibly CPL-active. Even if some dissymmetry were present, we might expect this to be far smaller than that of Eu^3+^ transitions and within the noise level of this measurement. By contrast, some $\genfrac{}{}{0ex}{}{5}{}{D}_{1}\,\to \,\genfrac{}{}{0ex}{}{7}{}{F}_{{J}_{2}}$ transitions possess evident luminescence dissymmetry. In particular, the ^5^*D*_1_ → ^7^*F*_2_ transition around 560 nm shows strong bisignate CPL with |*g*_lum_| ≈ 0.2 as might be expected, because it is a magnetic dipole transition similar to the strongly dissymmetric ^5^*D*_0_ → ^7^*F*_1_ transition at 595 nm (ref. ^40^).

To rule out ns-timescale linear polarization artefacts, we intentionally induced photoselection by exciting Eu[(+)-facam]_3_ with vertically polarized light and performed an *S*_1_ linear polarization measurement with 2 ns oversampled (partially overlapping) time bins (Fig. 3f–h). Linear polarization is measured in the LC emission band, almost completely disappearing within the instrument response (around 2 ns). Eu^3+^ features exhibit far less linear polarization, as might be expected, because these states are populated by energy transfer and not direct excitation. Our results are consistent with strong photoselection of ligand dipoles followed by rapid reorientation on a timescale within the instrument response (2 ns). Even when intentionally maximized, substantial linear polarization is not present on timescales used for the CPL measurements in Fig. 3c–e, increasing our confidence in those findings.

To summarize, TRCPL with ns time resolution allows us to observe several short-lived electronic transitions in a europium(III) complex and evaluate their CPL activity for the first time. Hence, the method provides a handle to uncover signals that are otherwise suppressed in steady-state and microsecond time-resolved measurements but may be crucial for understanding photophysical relaxation pathways.

## Chiral organic TADF emitter

**Fig. 4:**
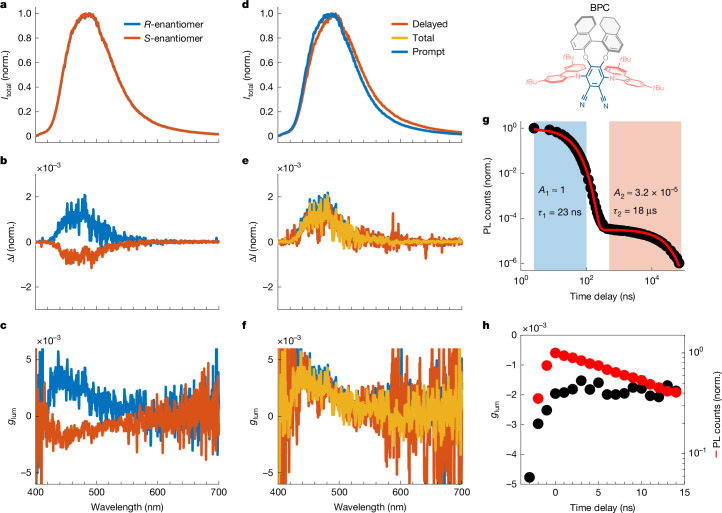
Steady-state and time-resolved CPL experiments of a chiral TADF-active dye BPH in toluene solution. Structure of the chiral TADF-active dye BPH is shown at the top right. **a**–**c**, Steady-state CPL spectra of (*R*)- and (*S*)-enantiomers (excitation 343 nm, 200 fs, 12.5 kHz). **d**–**f**, Time-resolved CPL spectra of the (*R*)-enantiomer. ‘Prompt’ refers to the first 100 ns, ‘Delayed’ refers to approximately 500 ns–80 μs, and ‘Total’ refers to a gate covering the complete emission process (0–80 μs). **g**, Kinetic trace of the total intensity decay integrated over the full spectrum, with highlighted regions showing the ‘Prompt’ and ‘Delayed’ time regions and parameters of a biexponential fit (red trace). **h**, Kinetic traces of the dissymmetry factor (average from 445–455 nm) and total intensity decay (integrated over 400–450 nm) with 3 ns time bins and 1 ns steps for the *S*-enantiomer (50 kHz repetition rate). Source Data

Although CPL-active lanthanide complexes provide a convenient benchmark, studies on chiral emitters often involve materials with much weaker dissymmetry and faster luminescence decay. For example, purely organic small molecules in solution rarely exceed dissymmetry factors of 10^−2^ even in best-performing materials^30,46^. Hence, we demonstrate the broad applicability and sensitivity of our TRCPL setup using a CPL-active organic molecule. To introduce multiple luminescence timescales, we used a chiral organic dye (*R*/*S*)-BINOL-phthalonitrile-tBuCz ((*R*/*S*)-BPC; Fig. 4), which shows thermally activated delayed fluorescence (TADF) and for which only steady-state CPL has been previously measured^47^.

Steady-state CPL spectra of the two enantiomers in toluene solution show the expected mirror-image CPL (Fig. 4a–c) with g_lum_ values of +1.8 × 10^−3^ (*R*) and –1.3 × 10^−3^ (*S*) at the luminescence peak, matching previous reports (|*g*_lum_| = 1.6 × 10^−3^) (ref. ^47^). To introduce time resolution, we performed two experiments: first, we separately gated the prompt, delayed and total emission components of *R*-BPC (Fig. 4d–f). The kinetic trace of the total emission (Fig. 4g) shows a biexponential decay process. Compared with the delayed component, prompt emission is much shorter-lived (23 ns compared with 18 μs), but more than 10,000 times more intense. Consequently, the total emission spectrum is dominated by prompt emission and prompt and total spectra overlap (Fig. 4d). There is a slight unexpected spectral shift between the prompt and delayed components. For TADF, the luminescent state is expected to be the same (^1^CT) in both time regimes. In principle, the shift could arise from delayed fluorescence overlapping with another triplet-mediated luminescence pathway. Regardless, the dissymmetry of the delayed component is not meaningfully affected, and the CPL/*g*_lum_ spectra (Fig. 4e,f) are indistinguishable within noise for the different components. This not only confirms that the measurement is sensitive enough to accurately quantify *g*_lum_ of the order of 10^−3^, but it can also do so reliably for a temporally separated emission component comprising less than 3% of the total emission intensity.

Finally, we demonstrate ns-scale kinetic traces of weak CPL with *S*-BPC (Fig. 4h). The first few bins show a rise caused by the instrument response. As convolution and division are not commutative, dissymmetry factors in this region should not be interpreted^48^. The measured *g*_lum_ remains constant over the remaining part of the measured 15-ns time range, demonstrating the ability to track *g*_lum_ values of the order of 10^−3^ on ns timescales. Temporal characterization of CPL in this material, which combines ns-scale and μs-scale decays with *g*_lum_ of the order of 10^−3^, would not be feasible with pre-existing techniques.

## Polarization artefacts and relaxation in achiral dye

**Fig. 5:**
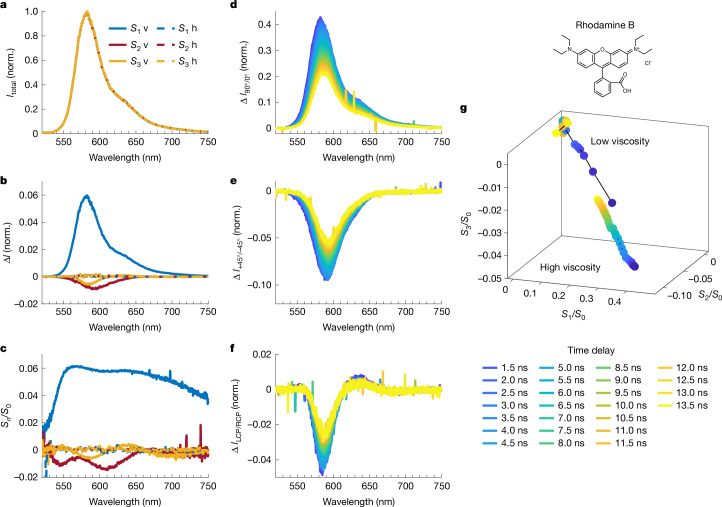
Broadband steady-state and time-resolved full Stokes vector spectroscopy of a standard achiral dye in aqueous solutions with low and high viscosity. Structure of rhodamine B is shown at the top right. **a**–**c**, Steady-state Stokes vector measurements (excitation 515 nm, 200 fs and 50 kHz) in a low-viscosity environment with horizontal (h) and vertical (v) excitation polarizations to inhibit and induce photoselection effects, respectively. Vertical excitation polarizations correspond to solid lines, and horizontal excitation polarizations to dashed lines. We point out that all spectra overlap in **a**, and in **b** and **c** the dashed lines are all flat about 0. **d**–**f**, Time-resolved intensity differences (excitation 515 nm, 200 fs and 50 kHz) over the Stokes polarization basis (normalized to total intensity maximum) in a high-viscosity environment with vertical excitation polarization to induce photoselection. **g**, Time evolution of all three Stokes components (averaged over a 10 nm range about the emission peak) in low-viscosity and high-viscosity environments with vertical excitation polarization (2 ns time bins and 0.5 ns time steps). Source Data

Finally, using the achiral dye rhodamine B (Fig. 5), we demonstrate the low-noise zero baseline of our setup when photoselection is minimized, and the time evolution of various apparent polarization components when photoselection is induced on purpose.

First, we measured the steady-state luminescence spectra of rhodamine B in water as a relatively low-viscosity environment with different excitation polarizations (Fig. 5a–c). Specifically, we used horizontal excitation polarization to minimize and vertical excitation polarization to intentionally maximize photoselection effects. The total emission spectrum is not affected by the excitation polarization (Fig. 5a) and horizontally polarized excitation yields a flat baseline about 0 with noise on the order of 10^−3^ or below for all polarization components *S*_1_, *S*_2_ and *S*_3_, as expected for an achiral molecule (Fig. 5b,c, dashed lines; also see Supplementary Fig. 13 for measurements showing *S*_1/2/3_ noise levels pushed to the order of 10^−4^).

When vertically polarized excitation is used, non-zero values for all polarization components appear (Fig. 5b,c, solid lines).

Expectedly, the *S*_1_ component (horizontal–vertical linear polarization) shows the greatest response (the *S*_2_ component at 45°/135° linear polarization may partially arise from a slight tilt of the excitation beam, so the excitation polarization is not perfectly vertical along the detection axis).

Importantly, we observe a substantial non-zero *S*_3_ component for an achiral sample, that is, a CPL artefact. CPL artefacts induced by linear polarization components are the main challenge in accurate CPL measurements^18,19^ across setup designs^31^. They are usually attributed to imperfections in the optical components, such as residual static birefringence and circular dichroism^18,31^.

For time-resolved CPL measurements, it is important to understand how the temporal evolution of linear polarization components translates to CPL artefacts. This requires time-evolving linear polarization luminescence on sufficiently long timescales to be measured.

In solution, photoselection-induced linear polarization decays as the excited molecules rotate and randomize the orientation of their transition dipoles. To vary the rotational relaxation times of our fluorophore and thus the linear polarization decay times, we increased the solvent viscosity^49^ by adding sucrose. This is an alternative to changing solvents completely, as the luminescence of rhodamine B is solvent-dependent^50^.

In a high-viscosity environment with maximized photoselection, a very large *S*_1_ component is present immediately after excitation (Fig. 5d) and decays over time. Smaller *S*_2_ and *S*_3_ components are also measured and depolarize over time (Fig. 5e,f). Their spectral shape and magnitude relative to *S*_1_ are consistent with the steady-state measurements in a low-viscosity medium (Fig. 5b).

Time evolution of the Stokes parameters about the luminescence peak in low- and high-viscosity solutions is shown in Fig. 5g. For the low-viscosity solution, depolarization is almost complete within the instrument response and the Stokes parameters are subsequently clustered around 0. For the high-viscosity solution, emission remains partially polarized after 10 ns, tracing an approximately straight line through the *S*_1_/*S*_2_/*S*_3_ space (Fig. 5g). The *S*_3_ component, representing a CPL artefact, is therefore proportional to the real *S*_1_ component (although considerably smaller in magnitude).

Our results reaffirm the importance of linear-polarization-induced CPL artefacts and, consequently, the importance of measuring linear polarization components. Even in isotropic samples, linear anisotropy may be induced by photoselection, and especially for high-viscosity solutions or solid-state samples these effects can influence the luminescence polarization dynamics. Even in these cases, the impact can be minimized by appropriate measurement parameters.

## Conclusion and discussion

We developed time-resolved broadband full-Stokes-vector luminescence spectroscopy as a versatile method for the polarization-resolved investigation of excited-state dynamics. Our design establishes broadband ns time resolution at ms range with a sensitivity noise floor of the order of 10^−4^.

We then demonstrated the use of this setup in probing various timescales and degrees of CPL activity.

First, we validated our setup using the CPL standard Eu[(+)-facam]_3_, for which we reproduced previously reported steady-state and μs-scale time-resolved spectra. Leveraging the ns time resolution of our instrument, we showed the polarization-resolved luminescence dynamics of ligand-centred excited states and high-energy *f*–*f* transitions in Eu^3+^.

The high sensitivity of our method allowed us to track the temporal evolution (early-time ns to late-time µs) of weak CPL signals in a chiral TADF emitter with dissymmetry factors of the order of 10^−3^, marking the first CPL dynamics report of such a material.

Last, we mapped out the temporal dynamics of polarization components and artefacts covering the full Stokes vector by deliberately introducing photoselection in an achiral dye. We show that CPL artefacts may also show time dynamics for certain timescales and experimental parameters, but also show that such artefacts can be effectively mitigated with appropriate experimental design.

To facilitate adoption by the wider community, we share the full setup design, along with the algorithms used for measurement automation. Furthermore, we provide a compendium of practical considerations, including non-obvious error sources such as beam deviation and variations in detector pixel sensitivity. These factors open new avenues to further push the limits of sensitivity and time resolution in the future, for example, by using optical gating for covering ultrafast timescales.

Overall, broadband time-resolved full-Stokes luminescence spectroscopy greatly expands the sensitivity, timescale and scope of accessible polarization information compared with the state-of-the-art methods. Importantly, this is achieved while retaining a straightforward design using stock components. We hope that our work will fuel the development of next-generation high-performance optical materials by revealing their underlying dynamics with unprecedented detail.

## Methods

### Separation of polarization components of sample luminescence

A schematic of the setup with specifications and part numbers of the main components are provided in Supplementary Fig. 1 and Supplementary Information section 1.1.

Sample luminescence is collected by a lens and passed through a set of polarization optics consisting of a superachromatic HWP, a superachromatic QWP and a 1° Wollaston prism. The HWP and QWP are placed in motorized rotation mounts, whereas the Wollaston prism remains fixed. After the polarization optics, the luminescence is focused onto a (vertical) spectrograph slit by a lens. The result is a free-space configuration with no fibre coupling or mirrors between the sample and spectrograph.

Polarization tracks are spatially separated (vertically) from the single luminescence beam by the fixed Wollaston prism. Therefore, one track will always correspond to a vertical polarization and the other to a horizontal polarization (which we will call horizontal and vertical tracks accordingly). Tracks are passed to a grating and focused onto an intensified CCD 2D-array detector (ICCD).

An illustration of how the various Stokes components produce intensity differences between the tracks in the ideal case with both a HWP and QWP present is presented in Fig. 1c. Waveplate orientations are defined about the fast axis relative to the table plane. Rotation of waveplates results in projecting the luminescence polarization components of interest (0°/90°, +45°/−45°, LCP/RCP for *S*_1_, *S*_2_ and *S*_3_, respectively) onto the horizontal or vertical tracks. The other waveplate orientation (varied by 45°/90° from the first for the HWP/QWP, respectively) is then used to swap the polarization components between the tracks. This combination of simultaneous (orthogonal polarizations measured at the same time on different tracks) and sequential (orthogonal polarizations measured on the same track after waveplate rotation) measurements allows for cancellation of major errors due to different transmissions along the two beam paths and time instability as previously described^31^.

We note that it is in principle possible to only use an HWP for conducting an *S*_1_/*S*_2_-only measurement and to only use a QWP for an *S*_3_-only measurement. Yet, for a faster, fully automatable full-Stokes measurement, it is advantageous to have both waveplates in place for all measurements. In all cases, the waveplate angles for measuring a given *S*_*n*_ component are such that the other two components are split evenly across the two channels and, therefore, are not measured as a false polarization signal in the ideal case. Importantly, for CPL measurements, the HWP orientation in principle does not matter, but in all orientations, the HWP will act to transform LCP to RCP and vice versa, which must be accounted for in processing.

Imperfections in real-life optics can produce polarization artefacts, including the well-known false CPL signals when substantial linear polarization is present^18^. It is, therefore, advisable in polarization measurements to take care when interpreting small polarization components in the presence of other, larger polarization components.

### Excitation/collection geometry and excitation polarization

Sample excitation is possible in various configurations, defined by the relative angle and polarization of the excitation beam with respect to the collection optics. In general, artefact-free CPL measurements are possible in two configurations: (1) with 90° excitation/collection geometry and horizontal excitation polarization or (2) with 180° excitation/collection and unpolarized excitation polarization^19^.

All data presented in this work were collected from solution samples, for which the 90° excitation/collection geometry with square-based four-window cuvettes was found to be the most straightforward approach (more details in Supplementary Information section 4.1). In this geometry, photoselection effects in collected luminescence (affecting the degree of induced linear polarization) can be minimized by using a horizontal excitation polarization and maximized by a vertical excitation polarization.

Horizontally polarized excitation was used for artefact-free CPL measurements. Vertically polarized excitation was used to intentionally induce photoselection and linear components in luminescence.

Excitation beam polarization states were set using a HWP and linear polarizer after the excitation light source.

### Sample preparation and measurement conditions

Eu[(+)-facam]_3_ was used as received from the supplier (Sigma-Aldrich). Rhodamine B was used as received from the supplier (Radiant Dyes). (*R*/*S*)-BINOL-phthalonitrile-tBuCz was synthesized according to previously described methods^47^.

Solution concentrations were 0.5 mM (non-polarization-resolved measurements) and 11 mM (polarization-resolved measurements) for Eu[(+)-facam]_3_ in DMSO, 0.6 mM for (*R*/*S*)-BINOL-phthalonitrile-tBuCz in toluene, 37 μM for rhodamine B in water, 33 μM for rhodamine B in a water:sucrose mixture.

Solution samples were prepared using anhydrous solvents in a nitrogen-filled glovebox, except for aqueous solutions of rhodamine B, which were prepared under ambient conditions. Aqueous solutions were prepared using distilled water. For high-viscosity solutions, sucrose was dissolved in water near the solubility limit (approx. 2 g ml^−1^). The Eu[(+)-facam]_3_ solution in ‘wet’ DMSO was prepared by adding 0.5% v/v deionized water to the 11 mM Eu[(+)-facam]_3_ solution, in which the solution was measured 5 h after the addition of water, similar to the methodology described in ref. ^16^.

Solutions were placed in screw-top four-windowed quartz cuvettes with a 1-cm square base. Air ingress to cuvettes was reduced by further sealing with PTFE tape and parafilm within the glovebox, where appropriate. Solutions were measured at ambient temperature in a laboratory with controlled and monitored temperature, recorded as 21 ± 0.3 °C for all measurements.

### Sample excitation

For time-resolved measurements, samples were optically excited using the output of a Light Conversion PHAROS laser (Yb:KGW lasing medium, 1,030 nm, pulse energy 400 μJ, pulse width duration 200 fs and repetition rate of 50 kHz). The pump beam was generated from the seed in a harmonic generation unit (Light Conversion HIRO) by nonlinear crystals (beta-barium borate and lithium triborate) with residual fundamental removed by dichroic mirrors within the unit. Second and third harmonics can be generated, giving pump wavelengths of 515 nm or 343 nm, respectively. Other excitation wavelengths were generated using an optical parametric amplifier (Light Conversion ORPHEUS-NEO). Pump pulse energy at the sample was 10–70 nJ, with the pump focused down to a beam diameter of approximately 1 mm on the sample. The laser repetition rate is controllable by a pulse picker, and repetition rates in the range of 0.5–50 kHz were used as specified, where data are presented.

For continuous wave excitation at 405 nm, a laser diode (ThorLabs DL5146-101S mounted in a ThorLabs LDM9T temperature-controlled mount) was used, with a constant output power of 5–50 mW.

### Signal collection and time ranges

The ICCD sensor has 2,048 × 512 pixels, enabling simultaneous recording of multiple tracks. Horizontal and vertical polarization components, spatially separated (on the vertical axis) by a Wollaston prism, are simultaneously recorded. The spectrograph grating splits the wavelengths in both tracks horizontally across the sensor. Vertical pixel binning is used to produce two effective vertical pixels for each wavelength pixel, giving *I*_h_(*λ*) and *I*_v_(*λ*) for the horizontal and vertical channels, respectively.

The intensifier of the ICCD only passes through a signal when a gate pulse is applied across it. Setting gate pulse values allows for adjusting the time over which luminescence is measured. For time-resolved measurements, the quantities recorded during a single acquisition are *I*_h_(*λ*, *t*) and *I*_v_(*λ*, *t*), where *t* is defined by the gate pulse applied. Time series are built up by repeating the measurement with modified gate delays and widths.

Accessible time bins range from approximately 2 ns to 2 ms for the described setup, with the lower limit arising from the intensifier specifications. The upper limit is practically limited by the laser repetition rate, as very long gate pulse values are achievable. In our setup, the laser operates at 50 kHz and can be pulse-picked for a lower frequency operation at the same per-pulse fluence (and lower time-averaged power). The practical limit for sufficient luminescence for measurements was found to be approximately 500 Hz, corresponding to a maximum time range of 2 ms; extending this would be reasonably straightforward by increasing per-pulse power at the sample, in principle.

For time-averaged values, comparable to steady-state measurements, a bin size spanning the entire time period between excitation pulses (or at least the time period during which luminescence is present) can be used with a pulsed excitation. Alternatively, the setup can be switched to a continuous wave excitation source, in which case only a time-averaged value is measured regardless of detector time gating.

### Data processing

Error cancellation and changing the Stokes polarization component measured require repeating the measurement with rotated waveplates. Subsequent data processing to remove dark background signals and obtain error-corrected polarization spectra and related quantities was carried out with simple scripts. Algorithms for measurement automation are presented in Supplementary Information section 5.

To denote the orientations of the HWP and QWP, we will call the recorded intensities *I*_v,QWP__*θ*__,HWP__*θ*_ and *I*_h,QWP__*θ*__,HWP__*θ*_ and drop the wavelength/time label for conciseness. The exact series of performed measurements depends on the specific experiment.

A simple *S*_3_ measurement measures *I*_LCP_ and *I*_RCP_, recorded as

$${I}_{{\rm{LCP}}}={I}_{{\rm{h}},{\rm{QWP}}4{5}^{^\circ },{\rm{HWP}}{0}^{^\circ }}+{I}_{{\rm{v}},{\rm{QWP}}13{5}^{^\circ },{\rm{HWP}}{0}^{^\circ }}$$

$${I}_{{\rm{RCP}}}={I}_{{\rm{v}},{\rm{QWP}}4{5}^{^\circ },{\rm{HWP}}{0}^{^\circ }}+{I}_{{\rm{h}},{\rm{QWP}}13{5}^{^\circ },{\rm{HWP}}{0}^{^\circ }}$$

From this, we may calculate the quantities

$$\Delta I={I}_{{\rm{LCP}}}-{I}_{{\rm{RCP}}}$$

$${I}_{{\rm{total}}}={S}_{0}=\,{I}_{{\rm{LCP}}}+{I}_{{\rm{RCP}}}$$

$${g}_{{\rm{lum}}}=\frac{{I}_{{\rm{LCP}}}-{I}_{{\rm{RCP}}}}{\frac{1}{2}({I}_{{\rm{LCP}}}+{I}_{{\rm{RCP}}})}$$

$${S}_{3}=\,{I}_{{\rm{RCP}}}-{I}_{{\rm{LCP}}}$$

with analogous processing steps for the linear components *S*_1_ and *S*_2_ for different waveplate angles and LCP/RCP replaced by the appropriate linear polarization axes (0°/90° and +45°/−45°, respectively). These are shown in Supplementary Information section 2.

Where appropriate, the transmission curve of long-pass filters used was measured and corrected for in data processing. Transmission characters of other optical components were not corrected for.

## Online content

Any methods, additional references, Nature Portfolio reporting summaries, source data, extended data, supplementary information, acknowledgements, peer review information; details of author contributions and competing interests; and statements of data and code availability are available at 10.1038/s41586-025-09197-3.

## Supplementary information

Supplementary Information This file contains Supplementary Text, Supplementary Figs., Supplementary Data, Practical Considerations for Measurements, Algorithms and Automation and Supplementary References.

Peer Review File

## Source data

Source Data Fig. 1

Source Data Fig. 2

Source Data Fig. 3

Source Data Fig. 4

Source Data Fig. 5

## Data Availability

Raw and processed data underlying the plots in this paper are available at Zenodo (10.5281/zenodo.15360970)^51^. Source data are provided with this paper.

## Code availability

Pseudocode describing the applied algorithms is provided in the Supplementary Information. The Python implementation for hardware control, which relies on a proprietary software development kit (SDK) for the specific camera used and therefore cannot be publicly released, is available from the corresponding author upon reasonable request.
