# Supplementary material for: Broadband transient full-Stokes luminescence spectroscopy
Source: Nature. 2025 Jun 25;643(8072):675–82. doi: 10.1038/s41586-025-09197-3 (PMC12267050; doi:10.1038/s41586-025-09197-3)
Supplement: Supplementary file 1 — This file contains Supplementary Text, Supplementary Figs., Supplementary Data, Practical Considerations for Measurements, Algorithms and Automation and Supplementary References. [file 41586_2025_9197_MOESM1_ESM.docx]

**Supplementary Information**

**Broadband Transient Full-Stokes Luminescence Spectroscopy**

Antti-Pekka M. Reponen^1^, Marcel Mattes^1,2^, Zachary A. VanOrman^1,2^, Lilian Estaque^3^, Grégory Pieters^3^, and Sascha Feldmann^1,2^*

^1^Rowland Institute, Harvard University, Cambridge, MA, USA

^2^Institute of Chemical Sciences and Engineering, École Polytechnique Fédérale de Lausanne, Lausanne, Switzerland

^3^Université Paris-Saclay, CEA, INRAE, Département Médicaments et Technologies pour la Santé, Gif-sur-Yvette, France

*Email: sascha.feldmann@epfl.ch

**1: Setup Description**

**1.1: List of Setup Components**

Pulsed excitation source: Light Conversion PHAROS (1030 nm, 180 fs, 50 kHz, variable repetition rate with pulse picker); Light Conversion HIRO harmonic unit output at 515 nm/343 nm, or Light Conversion ORPHEUS-NEO OPA output used for other excitation wavelengths.

CW excitation source: ThorLabs DL5146-101S 405 nm laser diode

Wollaston prism: ThorLabs WPQ10 1⁰ beam separation, uncoated quartz, 400-2000 nm

Quarter-wave plate: ThorLabs SAQWP05M-700 superachromatic 325-1100 nm

Half-wave plate (detection/excitation): ThorLabs SAHWP05M-700 superachromatic 310-1100 nm

Linear polarizer (excitation): ThorLabs WP25M-UB

Waveplate motorized high-precision rotational housing: ThorLabs PRM1/MZ8

Waveplate housing motor controller: ThorLabs KDC101

L2: Focusing lens (excitation): ThorLabs LA4579 UV fused silica, uncoated, f = 301.1 mm plano-convex

L3: Collimating lens (detection): ThorLabs LA4380 UV fused silica, uncoated, f = 100.3 mm plano-convex

L4: Focusing lens (detection): ThorLabs AC508-100-A

F: 375/450/530 nm longpass filter (depending on excitation source)

Spectrograph: Andor Kymera 328i

Grating: SR-GRT-0150-0500 150 l/mm 500 nm blaze reflective grating

Detector: Andor DH340T-18U-74 gated intensified CCD camera (iStar iCCD)


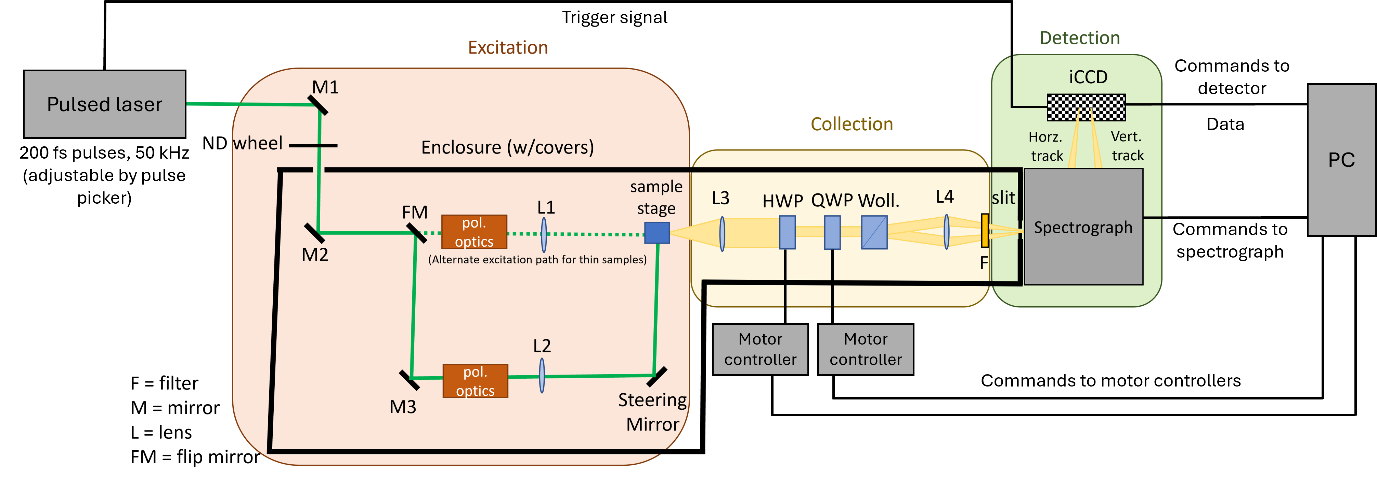


*Figure S1: Setup schematic showing the main components, beam paths and electronic signals in the instrument.*

*
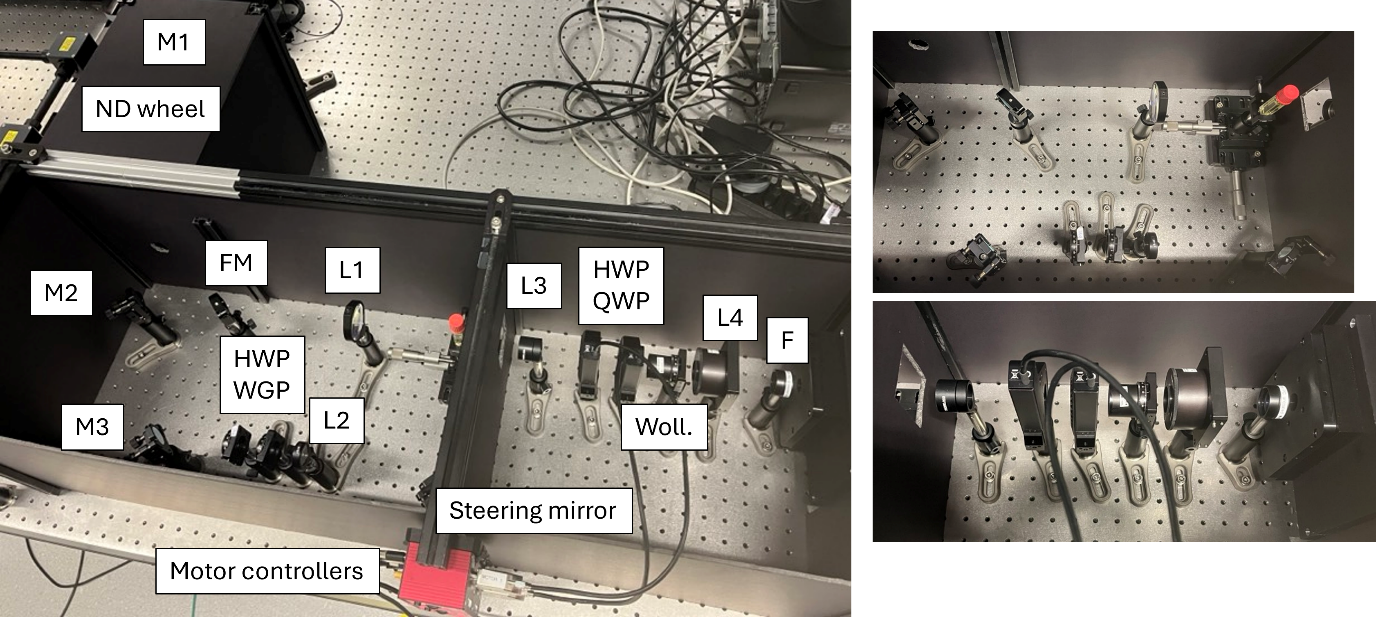
*

*Figure S2: Pictures of the setup, with parts laid out and labelled as in Figure S1. WGP refers to a wire grid polarizer.*

**1.2: PEM-based CPL setup for data comparison**

To establish consistency of our approach with standard photoelastic modulator (PEM)-based CPL detection systems, we present in the SI some steady-state CPL (SSCPL) spectra using a home-built PEM-based setup. Setup specifics and details of its operation are described at length in our earlier work (*18*).

The setup consists of the following main components:

Excitation source: 365 nm LED (Thorlabs Solis 365C)

PEM: Hinds PEM200

Linear polarizer: Thorlabs WP25M-UB

Monochromator: Acton SP-2155

PMT: Hamamatsu H10723-20

Lock-in amplifier: Zürich Instruments MFLI 5 MHz

To block scattered excitation light, a 365 nm bandpass filter was placed between the LED and the sample and a 380 nm longpass filter was placed between the sample and detector.

**2: Supplementary Text**

**2.1: Measured and Calculated Quantities**

As described in the Methods, two tracks are simultaneously recorded at a given orientation of the HWP and QWP. As these tracks are separated after the waveplates by a fixed Wollaston prism, one track always will correspond to vertical polarization and the other to horizontal polarization. Accordingly, we will call the recorded intensities $I_{v,QWPϴ,HWPϴ}$ and $I_{h,QWPϴ,HWPϴ}$.

An $S_{3}$ measurement would then seek to find $I_{LCP}$ and $I_{RCP}$, recorded as

$$I_{LCP}=I_{h,QWP45^{\circ},HWP0^{\circ}}+I_{v,QWP135^{\circ},HWP0^{\circ}}$$

$$I_{RCP}=I_{v,QWP45^{\circ},HWP0^{\circ}}+I_{h,QWP135^{\circ},HWP0^{\circ}}$$

from which we calculate the quantities

$$\Delta I=I_{LCP}-I_{RCP}$$

$$I_{total}=I_{LCP}+I_{RCP}$$

Whereas for an $S_{1}$measurement we have

$$I_{0⁰}=I_{h,QWP0^{\circ},HWP0^{\circ}}+I_{v,QWP0^{\circ},HWP45^{\circ}}$$

$$I_{90⁰}=I_{v,QWP0^{\circ},HWP0^{\circ}}+I_{h,QWP0^{\circ},HWP45^{\circ}}$$

$$\Delta I=I_{0⁰}-I_{90⁰}$$

and for an $S_{2}$measurement

$$I_{+45⁰}=I_{h,QWP0^{\circ},HWP22.5^{\circ}}+I_{v,QWP0^{\circ},HWP67.5^{\circ}}$$

$$I_{-45⁰}=I_{v,QWP0^{\circ},HWP22.5^{\circ}}+I_{h,QWP0^{\circ},HWP67.5^{\circ}}$$

$$\Delta I=I_{+45⁰}-I_{-45⁰}$$

While two QWP/HWP positions are sufficient for cancelling out errors associated with static channel transmission imbalances and time instabilities affecting both channels (as illustrated in Figure S3), there are four QWP positions (for $S_{3}$) and eight HWP positions (for $S_{1}/S_{2}$ each) which could in principle be used (in pairs). In some scenarios (e.g. as described in SI section 4.7), it is useful to measure over more than two waveplate positions.

An $S_{3}$ measurement in such a case would then additionally measure ${I'}_{LCP}$ and ${I'}_{RCP}$, recorded as

$${I'}_{LCP}=I_{h,QWP225^{\circ},HWP0^{\circ}}+I_{v,QWP315^{\circ},HWP0^{\circ}}$$

$${I'}_{RCP}=I_{v,QWP225^{\circ},HWP0^{\circ}}+I_{h,QWP315^{\circ},HWP0^{\circ}}$$

From which we calculate the quantities

$$\Delta I={[(I}_{LCP}+{I'}_{LCP})-(I_{RCP}+{I^{'}}_{RCP})]$$

$$I_{total}={[(I}_{LCP}+{I'}_{LCP})+(I_{RCP}+{I^{'}}_{RCP})]$$

For an $S_{1}$measurement, one could similarly measure some of the additional pairs of angles to measure and calculate the following quantities:

$${I'}_{0⁰}=I_{h,QWP0^{\circ},HWP90^{\circ}}+I_{v,QWP0^{\circ},HWP135^{\circ}}$$

$${I'}_{90⁰}=I_{v,QWP0^{\circ},HWP90^{\circ}}+I_{h,QWP0^{\circ},HWP135^{\circ}}$$

$${I''}_{0⁰}=I_{h,QWP0^{\circ},HWP180^{\circ}}+I_{v,QWP0^{\circ},HWP225^{\circ}}$$

$${I''}_{90⁰}=I_{v,QWP0^{\circ},HWP180^{\circ}}+I_{h,QWP0^{\circ},HWP225^{\circ}}$$

$${I'''}_{0⁰}=I_{h,QWP0^{\circ},HWP270^{\circ}}+I_{v,QWP0^{\circ},HWP315^{\circ}}$$

$${I'''}_{90⁰}=I_{v,QWP0^{\circ},HWP270^{\circ}}+I_{h,QWP0^{\circ},HWP315^{\circ}}$$

$$\Delta I=[(I_{0^{0}}+{I^{'}}_{0^{0}}+{I^{''}}_{0^{0}}+{I'''}_{0⁰})-(I_{{90}^{0}}+{I^{'}}_{{90}^{0}}+{I^{''}}_{{90}^{0}}+{I'''}_{90⁰})]$$

$$I_{total}=[\left( I_{0^{0}}+{I^{'}}_{0^{0}}+{I^{''}}_{0^{0}}+{I^{'''}}_{0^{0}} \right)+(I_{{90}^{0}}+{I^{'}}_{90^{0}}+ {I^{''}}_{{90}^{0}}+{I'''}_{90⁰})]$$

And similarly, for an $S_{2}$measurement:

$${I'}_{+45⁰}=I_{h,QWP0^{\circ},HWP112.5^{\circ}}+I_{v,QWP0^{\circ},HWP157.5^{\circ}}$$

$${I'}_{-45⁰}=I_{v,QWP0^{\circ},HWP112.5^{\circ}}+I_{h,QWP0^{\circ},HWP157.5^{\circ}}$$

$${I''}_{+45⁰}=I_{h,QWP0^{\circ},HWP202.5^{\circ}}+I_{v,QWP0^{\circ},HWP247.5^{\circ}}$$

$${I''}_{-45⁰}=I_{v,QWP0^{\circ},HWP202.5^{\circ}}+I_{h,QWP0^{\circ},HWP247.5^{\circ}}$$

$${I'''}_{+45⁰}=I_{h,QWP0^{\circ},HWP292.5^{\circ}}+I_{v,QWP0^{\circ},HWP337.5^{\circ}}$$

$${I'''}_{-45⁰}=I_{v,QWP0^{\circ},HWP292.5^{\circ}}+I_{h,QWP0^{\circ},HWP337.5^{\circ}}$$

$$\Delta I=[(I_{{+45}^{0}}+{I'}_{+45⁰}+{I^{''}}_{{+45}^{0}}+{I'''}_{+45⁰})-(I_{{-45}^{0}}+{I^{'}}_{{-45}^{0}}+{I^{''}}_{{-45}^{0}}+{I'''}_{-45⁰})]$$

$$I_{total}=[(I_{{+45}^{0}}+{I'}_{+45⁰}+{I^{''}}_{{+45}^{0}}+{I'''}_{+45⁰})+(I_{{-45}^{0}}+{I^{'}}_{{-45}^{0}}+{I^{''}}_{{-45}^{0}}+{I'''}_{-45⁰})]$$

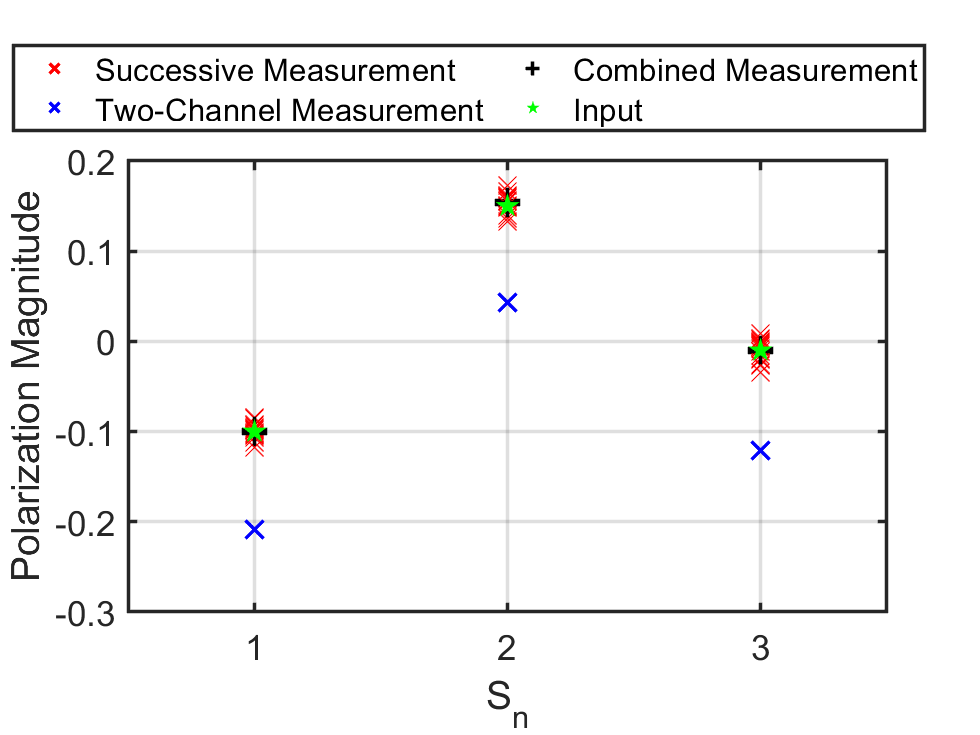


*Figure S3:* *Simulated example illustrating how combining successive measurements by waveplate rotation and simultaneous measurements by two-channel detection can accurately recover all Stokes components even with substantial spatial and temporal error sources. Here, track transmission mismatch is set to 20% and a random time drift set to 5% of the incident light intensity for this case.*

**2.2: Stokes Components vs. Anisotropy/Dissymmetry Values**

The Stokes components are defined as

$$S_{0}= I_{0^{\circ}}+I_{90^{\circ}}= I_{+45^{\circ}}+I_{-45^{\circ}}=I_{LCP}+I_{RCP}$$

$$S_{1}= I_{0^{\circ}}-I_{90^{\circ}}$$

$$S_{2}= I_{+45^{\circ}}-I_{-45^{\circ}}$$

$$S_{3}= I_{RCP}-I_{LCP}$$

Since the absolute count numbers are generally not of interest, for convenience we may scale the data such that ${\max(S}_{0})=1$. Alternatively, we can calculate the relative quantity $\frac{S_{1/2/3}}{S_{0}}$ in cases where the polarized proportion at each wavelength is to be presented.

Note that the quantities $S_{1/2/3}$ (even if normalized by $S_{0}$) which describe the polarization state of light are similar but not exactly the same as the commonly used quantities for describing material properties; these being the linear polarization anisotropy ($r$) and circular polarization dissymmetry of the luminescence ($g_{lum}$) which are instead defined as (*48*):

$$r=\frac{I_{\parallel}-I_{\perp}}{I_{\parallel}+2I_{\perp}}$$

where $I_{\parallel}$ and $I_{\perp}$are parallel and perpendicular linearly polarized intensities, and

$$g_{lum}=\frac{I_{LCP}-I_{RCP}}{I_{avg}}=\frac{I_{LCP}-I_{RCP}}{\frac{1}{2}(I_{LCP}+I_{RCP})}$$

Therefore, some care is required when comparing to other work where different conventions may be used. Our main literature comparisons relate to circular dissymmetries, and therefore, where only CPL is measured, we report $g_{lum}$ to make those comparisons simpler. When multiple polarization components are presented, we use the Stokes parameters for internal consistency.

**2.3: Müller Calculus Representation of the Measurement**

For convenience and clarity, basic principles of the measurements are shown here. A thorough error analysis treatment for CPL measurements specifically (equivalent to S_3_ measurements here) is found in the work of Baguenard *et al.*(*31*) which we do not aim to reproduce in this work.

We begin with the collecting lens collimating a portion of light emitted by the sample. This light has a polarization state represented by its Stokes vector

$$S_{in}=\left( \begin{matrix} S_{0} \\ S_{1} \\ S_{2} \\ S_{3} \end{matrix} \right)$$

The QWP and HWP have Müller matrices (when their fast axis is vertical) of

$$M_{QWP}=\left( \begin{matrix} 1 & 0 & 0 & 0 \\ 0 & 1 & 0 & 0 \\ 0 & 0 & 0 & -1 \\ 0 & 0 & 1 & 0 \end{matrix} \right)$$

$$M_{HWP}=\left( \begin{matrix} 1 & 0 & 0 & 0 \\ 0 & 1 & 0 & 0 \\ 0 & 0 & -1 & 0 \\ 0 & 0 & 0 & -1 \end{matrix} \right)$$

For arbitrary waveplate orientations *θ*, we use the rotation and inverse rotation matrixes

$$R(\theta)=\left( \begin{matrix} 1 & 0 & 0 & 0 \\ 0 & cos(2\theta) & sin(2\theta) & 0 \\ 0 & -sin(2\theta) & cos(2\theta) & 0 \\ 0 & 0 & 0 & 1 \end{matrix} \right)$$

$$R^{-1}(\theta)=\left( \begin{matrix} 1 & 0 & 0 & 0 \\ 0 & cos(2\theta) & -sin(2\theta) & 0 \\ 0 & sin(2\theta) & cos(2\theta) & 0 \\ 0 & 0 & 0 & 1 \end{matrix} \right)$$

The Wollaston prism (which remains in a fixed orientation) can be represented by separating the beam into two channels passing through orthogonal linear polarizers with

$$P_{horizontal}=\frac{1}{2}\left( \begin{matrix} 1 & 1 & 0 & 0 \\ 1 & 1 & 0 & 0 \\ 0 & 0 & 0 & 0 \\ 0 & 0 & 0 & 0 \end{matrix} \right)$$

$$P_{vertical}=\frac{1}{2}\left( \begin{matrix} 1 & -1 & 0 & 0 \\ -1 & 1 & 0 & 0 \\ 0 & 0 & 0 & 0 \\ 0 & 0 & 0 & 0 \end{matrix} \right)$$

The Stokes vectors of the two tracks at detection are then given by

$$S_{horizontal}=P_{horizontal}R^{-1}\left( \theta_{QWP} \right)M_{QWP}R\left( \theta_{QWP} \right)R^{-1}\left( \theta_{HWP} \right)M_{HWP}R\left( \theta_{HWP} \right)S_{in}=M_{h}S_{in}$$

$$S_{vertical}=P_{vertical}R^{-1}\left( \theta_{QWP} \right)M_{QWP}R(\theta_{QWP}) R^{-1}\left( \theta_{HWP} \right)M_{HWP}R(\theta_{HWP}) S_{in}=M_{v}S_{in}$$

where we have collected the total system matrices for the two channels as $M_{h}$ and $M_{v}$ and the polarization of the luminescence is described by the Stokes vector *S*_in_. Though the output at detection is polarized in each case (being separated to horizontal/vertical components by the Wollaston prism), the detector itself only measures the total intensity (the Stokes component *S*_0_). This is recorded as $I_{h}$ and $I_{v}$ for the horizontal and vertical channels, respectively.

[NB: More properly the detection elements, especially the grating, have some degree of polarization sensitivity. However, as the Wollaston is fixed, this will act analogously to a fixed transmission imbalance between the channels.]

To begin with, for an $S_{1}$measurement we would use $\theta_{QWP}=0^{\circ}$ with $\theta_{HWP}=0^{\circ}$ and 45°

Then for $\theta_{HWP}=0$

$S_{horizontal}= \frac{1}{2}\left( \begin{matrix} 1 & 1 & 0 & 0 \\ 1 & 1 & 0 & 0 \\ 0 & 0 & 0 & 0 \\ 0 & 0 & 0 & 0 \end{matrix} \right)\left( \begin{matrix} S_{0} \\ S_{1} \\ S_{2} \\ S_{3} \end{matrix} \right)=\left( \begin{matrix} \frac{1}{2}(S_{0}+S_{1}) \\ \frac{1}{2}(S_{0}+S_{1}) \\ 0 \\ 0 \end{matrix} \right)$ giving $I_{h,0^{\circ}}=\frac{1}{2}(S_{0}+S_{1})$

$S_{vertical}= \frac{1}{2}\left( \begin{matrix} 1 & -1 & 0 & 0 \\ -1 & 1 & 0 & 0 \\ 0 & 0 & 0 & 0 \\ 0 & 0 & 0 & 0 \end{matrix} \right)\left( \begin{matrix} S_{0} \\ S_{1} \\ S_{2} \\ S_{3} \end{matrix} \right)=\left( -\begin{matrix} \frac{1}{2}(S_{0}-S_{1}) \\ \frac{1}{2}(S_{0}-S_{1}) \\ 0 \\ 0 \end{matrix} \right)$ giving $I_{v,0^{\circ}}=\frac{1}{2}(S_{0}-S_{1})$

and for $\theta_{HWP}=$ $45⁰$

$S_{horizontal}= \frac{1}{2}\left( \begin{matrix} 1 & -1 & 0 & 0 \\ 1 & -1 & 0 & 0 \\ 0 & 0 & 0 & 0 \\ 0 & 0 & 0 & 0 \end{matrix} \right)\left( \begin{matrix} S_{0} \\ S_{1} \\ S_{2} \\ S_{3} \end{matrix} \right)=\left( \begin{matrix} \frac{1}{2}(S_{0}-S_{1}) \\ \frac{1}{2}(S_{0}-S_{1}) \\ 0 \\ 0 \end{matrix} \right)$ giving $I_{h,45^{\circ}}=\frac{1}{2}(S_{0}-S_{1})$

$S_{vertical}= \frac{1}{2}\left( \begin{matrix} 1 & 1 & 0 & 0 \\ -1 & -1 & 0 & 0 \\ 0 & 0 & 0 & 0 \\ 0 & 0 & 0 & 0 \end{matrix} \right)\left( \begin{matrix} S_{0} \\ S_{1} \\ S_{2} \\ S_{3} \end{matrix} \right)=\left( -\begin{matrix} \frac{1}{2}(S_{0}+S_{1}) \\ \frac{1}{2}(S_{0}+S_{1}) \\ 0 \\ 0 \end{matrix} \right)$ giving $I_{v,45^{\circ}}=\frac{1}{2}(S_{0}+S_{1})$

For an $S_{2}$measurement we would use $\theta_{QWP}=0$ and $\theta_{HWP}=22.5⁰$ and $67.5⁰$

Then for $\theta_{HWP}=22.5⁰$

$S_{horizontal}= \frac{1}{2}\left( \begin{matrix} 1 & 0 & 1 & 0 \\ 1 & 0 & 1 & 0 \\ 0 & 0 & 0 & 0 \\ 0 & 0 & 0 & 0 \end{matrix} \right)\left( \begin{matrix} S_{0} \\ S_{1} \\ S_{2} \\ S_{3} \end{matrix} \right)=\left( \begin{matrix} \frac{1}{2}(S_{0}+S_{2}) \\ \frac{1}{2}(S_{0}+S_{2}) \\ 0 \\ 0 \end{matrix} \right)$ giving $I_{h,22.5^{\circ}}=\frac{1}{2}(S_{0}+S_{2})$

$S_{vertical}= \frac{1}{2}\left( \begin{matrix} 1 & 0 & -1 & 0 \\ -1 & 0 & 1 & 0 \\ 0 & 0 & 0 & 0 \\ 0 & 0 & 0 & 0 \end{matrix} \right)\left( \begin{matrix} S_{0} \\ S_{1} \\ S_{2} \\ S_{3} \end{matrix} \right)=\left( -\begin{matrix} \frac{1}{2}(S_{0}-S_{2}) \\ \frac{1}{2}(S_{0}-S_{2}) \\ 0 \\ 0 \end{matrix} \right)$ giving $I_{v,22.5^{\circ}}=\frac{1}{2}(S_{0}-S_{2})$

and for $\theta_{HWP}=$ $67.5⁰$

$S_{horizontal}= \frac{1}{2}\left( \begin{matrix} 1 & 0 & -1 & 0 \\ 1 & 0 & -1 & 0 \\ 0 & 0 & 0 & 0 \\ 0 & 0 & 0 & 0 \end{matrix} \right)\left( \begin{matrix} S_{0} \\ S_{1} \\ S_{2} \\ S_{3} \end{matrix} \right)=\left( \begin{matrix} \frac{1}{2}(S_{0}-S_{2}) \\ \frac{1}{2}(S_{0}-S_{2}) \\ 0 \\ 0 \end{matrix} \right)$ giving $I_{h,67.5^{\circ}}=\frac{1}{2}(S_{0}-S_{2})$

$S_{vertical}= \frac{1}{2}\left( \begin{matrix} 1 & 0 & 1 & 0 \\ -1 & 0 & -1 & 0 \\ 0 & 0 & 0 & 0 \\ 0 & 0 & 0 & 0 \end{matrix} \right)\left( \begin{matrix} S_{0} \\ S_{1} \\ S_{2} \\ S_{3} \end{matrix} \right)=\left( -\begin{matrix} \frac{1}{2}(S_{0}+S_{2}) \\ \frac{1}{2}(S_{0}+S_{2}) \\ 0 \\ 0 \end{matrix} \right)$ giving $I_{v,67.5^{\circ}}=\frac{1}{2}(S_{0}+S_{2})$

Finally, for an $S_{3}$measurement we would use $\theta_{QWP}={45}^{0} \mathrm{and} 135⁰$ with $\theta_{HWP}=0$ (or any other HWP angle, as the HWP will invert the handedness of incident light regardless of its orientation, effectively inverting the sign of $S_{3}$).

Then for $\theta_{QWP}=$ $45⁰$

$S_{horizontal}= \frac{1}{2}\left( \begin{matrix} 1 & 0 & 0 & -1 \\ 1 & 0 & 0 & -1 \\ 0 & 0 & 0 & 0 \\ 0 & 0 & 0 & 0 \end{matrix} \right)\left( \begin{matrix} S_{0} \\ S_{1} \\ S_{2} \\ S_{3} \end{matrix} \right)=\left( \begin{matrix} \frac{1}{2}(S_{0}-S_{3}) \\ \frac{1}{2}(S_{0}-S_{3}) \\ 0 \\ 0 \end{matrix} \right)$ giving $I_{h,45^{\circ}}=\frac{1}{2}(S_{0}-S_{3})$

$S_{vertical}= \frac{1}{2}\left( \begin{matrix} 1 & 0 & 0 & 1 \\ -1 & 0 & 0 & -1 \\ 0 & 0 & 0 & 0 \\ 0 & 0 & 0 & 0 \end{matrix} \right)\left( \begin{matrix} S_{0} \\ S_{1} \\ S_{2} \\ S_{3} \end{matrix} \right)=\left( -\begin{matrix} \frac{1}{2}(S_{0}+S_{3}) \\ \frac{1}{2}(S_{0}+S_{3}) \\ 0 \\ 0 \end{matrix} \right)$ giving $I_{v,45^{\circ}}=\frac{1}{2}(S_{0}+S_{3})$

and for $\theta_{QWP}=$ $135⁰$

$S_{horizontal}= \frac{1}{2}\left( \begin{matrix} 1 & 0 & 0 & 1 \\ 1 & 0 & 0 & 1 \\ 0 & 0 & 0 & 0 \\ 0 & 0 & 0 & 0 \end{matrix} \right)\left( \begin{matrix} S_{0} \\ S_{1} \\ S_{2} \\ S_{3} \end{matrix} \right)=\left( \begin{matrix} \frac{1}{2}(S_{0}+S_{3}) \\ \frac{1}{2}(S_{0}+S_{3}) \\ 0 \\ 0 \end{matrix} \right)$ giving $I_{h,135^{\circ}}=\frac{1}{2}(S_{0}+S_{3})$

$S_{vertical}= \frac{1}{2}\left( \begin{matrix} 1 & 0 & 0 & -1 \\ -1 & 0 & 0 & 1 \\ 0 & 0 & 0 & 0 \\ 0 & 0 & 0 & 0 \end{matrix} \right)\left( \begin{matrix} S_{0} \\ S_{1} \\ S_{2} \\ S_{3} \end{matrix} \right)=\left( -\begin{matrix} \frac{1}{2}(S_{0}-S_{3}) \\ \frac{1}{2}(S_{0}-S_{3}) \\ 0 \\ 0 \end{matrix} \right)$ giving $I_{v,135^{\circ}}=\frac{1}{2}(S_{0}-S_{3})$

As can be seen, the outputs for $S_{2}$ and $S_{3}$ are analogous to results obtained for $S_{1},$ and therefore treatment for recovering the Stokes components is very similar in each case. Accordingly, we only explicitly show the process for $S_{1}$.

We end up with the various measured intensities $I$:

$I_{h,0^{\circ}}=\frac{1}{2}(S_{0}+S_{1})$ $I_{v,0^{\circ}}=\frac{1}{2}(S_{0}-S_{1})$

$I_{h,45^{\circ}}=\frac{1}{2}(S_{0}-S_{1})$ $I_{v,45^{\circ}}=\frac{1}{2}(S_{0}+S_{1})$

from which we can straightforwardly acquire the $S_{0}$ or $S_{1}$ components.

As currently defined, having two waveplate orientations and spatial channels appears redundant. However, we need to consider the effect of imperfections which result in intensity differences between channels for reasons besides the presence of real polarization components. The strongest effects are usually those proportional to total luminescence intensity, which can be roughly grouped under transmission imbalances and time drift-like effects.

*Transmission Imbalance*

A transmission imbalance between the horizontal and vertical channels can result either from imperfectly uniform beam paths or unequal polarization responses. We can represent this by adding transmission factors $T_{h}$ and $T_{v}$ (unknown numbers between 0 and 1) to the horizontal and vertical paths respectively:

$$S_{horizontal}={T_{h}M}_{h}S_{in}$$

$$S_{vertical}={{T_{v}M}_{v}S}_{in}$$

The outputs are then:

$I_{h,0^{\circ}}=\frac{1}{2}T_{h}\left( S_{0}+S_{1} \right)$ $I_{v,0^{\circ}}=\frac{1}{2}T_{v}(S_{0}-S_{1})$

$I_{h,45^{\circ}}=\frac{1}{2}T_{h}\left( S_{0}-S_{1} \right)$ $I_{v,45^{\circ}}=\frac{1}{2}T_{v}(S_{0}+S_{1})$

Taking the $I_{h}$ channels (*i.e*. taking measurements measured with the same polarization channel at different times), we can solve to acquire

$S_{0}=T_{h}\left( I_{h,0^{\circ}}+I_{h,45^{\circ}} \right)$ $S_{1}=T_{h}\left( I_{h,0^{\circ}}-I_{h,45^{\circ}} \right)$

Equivalently we could have used the $I_{v}$ channels to acquire

$S_{0}=T_{v}\left( I_{v,45^{\circ}}+I_{v,0^{\circ}} \right)$ $S_{1}=T_{v}\left( I_{v,45^{\circ}}-I_{v,0^{\circ}} \right)$

In both cases we can recover $S_{0}$ and $S_{1}$ which are scaled by a common quantity $T_{h/v}$. Since absolute count numbers are generally not of interest (in any case the counts reaching the detector will vary with measurement parameters), this allows us to recover the main quantities of interest, *i.e.* the relative magnitudes of $S_{0}$ and $S_{1}$.

*Time Drift*

It is common to have some time-dependent intensity variation between temporally separated measurements. As both channels are measured simultaneously, this will only affect measurements taken at different waveplate orientations. This “drift” could therefore also incorporate changes in transmission caused by waveplate rotation itself (though for error cancelling, these must not be channel-dependent). For this, we would add drift factors $d_{0^{\circ}}$ and $d_{45^{\circ}}$ for the measurements taken at HWP angles 0° and 45⁰, respectively:

$I_{h,0^{\circ}}=\frac{1}{2}d_{0^{\circ}}\left( S_{0}+S_{1} \right)$ $I_{v,0^{\circ}}=\frac{1}{2}d_{0^{\circ}}(S_{0}-S_{1})$

$I_{h,45^{\circ}}=\frac{1}{2}d_{45^{\circ}}\left( S_{0}-S_{1} \right)$ $I_{v,45^{\circ}}=\frac{1}{2}d_{45^{\circ}}(S_{0}+S_{1})$

In this case, we can take the $I_{0^{\circ}}$ channels (*i.e.* taking data measured at the same time with different polarization channels), to acquire

$S_{0}=d_{0^{\circ}}\left( I_{h,0^{\circ}}+I_{v,0^{\circ}} \right)$ $S_{1}=d_{0^{\circ}}\left( I_{h,0^{\circ}}-I_{v,0^{\circ}} \right)$

Equivalently, we could have used the $I_{45^{\circ}}$ channels to acquire

$S_{0}=d_{45^{\circ}}\left( I_{h,45^{\circ}}+I_{v,45^{\circ}} \right)$ $S_{1}=d_{45^{\circ}}\left( I_{v,45^{\circ}}-I_{h,45^{\circ}} \right)$

Where again we can recover $S_{0}$ and $S_{1}$ which are scaled by a common quantity $d_{0/45^{\circ}}$ and therefore recover the main quantities of interest, *i.e.* the relative magnitudes of $S_{0}$ and $S_{1}$.

*Combined Imperfections*

If both time drift and transmission imbalance are present, following the same treatment as before, we end up with:

$I_{h,0}=\frac{1}{2}{d_{0^{\circ}}T}_{h}\left( S_{0}+S_{1} \right)$ $I_{v,0}=\frac{1}{2}{d_{0^{\circ}}T}_{v}(S_{0}-S_{1})$

$I_{h,45}=\frac{1}{2}{d_{45^{\circ}}T}_{h}\left( S_{0}-S_{1} \right)$ $I_{v,45}=\frac{1}{2}d_{45^{\circ}}T_{v}(S_{0}+S_{1})$

This time, it is clear that no combination of two measurements will cancel out errors.

Following Baguenard *et al.* we can calculate the average intensity difference between the two tracks across the two HWP positions as (*31*):

$$\Delta I=\left( I_{h,0^{\circ}}+I_{v,45^{\circ}} \right)-(I_{v,0^{\circ}}+I_{h,45^{\circ}})$$

$$=\frac{1}{2}\left[ \left( {d_{0^{\circ}}T}_{h}\left( S_{0}+S_{1} \right)+ d_{45^{\circ}}T_{v}(S_{0}+S_{1}) \right)-({d_{0^{\circ}}T}_{v}\left( S_{0}-S_{1} \right)+{d_{45^{\circ}}T}_{h}\left( S_{0}-S_{1} \right)) \right]$$

$$=\frac{1}{2}[\left( {d_{0^{\circ}}T}_{h}+d_{45^{\circ}}T_{v}+{d_{0^{\circ}}T}_{v}+{d_{45^{\circ}}T}_{h} \right)S_{1}+\left( {d_{0^{\circ}}T}_{h}+d_{45^{\circ}}T_{v}-{d_{0^{\circ}}T}_{v}-{d_{45^{\circ}}T}_{h} \right)S_{0}]$$

Similarly, the combined total intensity as

$$I_{total}=\left( I_{h,0^{\circ}}+I_{v,45^{\circ}} \right)+(I_{v,0^{\circ}}+I_{h,45^{\circ}})$$

$$=\frac{1}{2}\left[ \left( {d_{0^{\circ}}T}_{h}\left( S_{0}+S_{1} \right)+ d_{45^{\circ}}T_{v}(S_{0}+S_{1}) \right)+({d_{0^{\circ}}T}_{v}\left( S_{0}-S_{1} \right)+{d_{45^{\circ}}T}_{h}\left( S_{0}-S_{1} \right)) \right]$$

$$=\frac{1}{2}[\left( {d_{0^{\circ}}T}_{h}+d_{45^{\circ}}T_{v}-{d_{0^{\circ}}T}_{v}-{d_{45^{\circ}}T}_{h} \right)S_{1}+\left( {d_{0^{\circ}}T}_{h}+d_{45^{\circ}}T_{v}+{d_{0^{\circ}}T}_{v}+{d_{45^{\circ}}T}_{h} \right)S_{0}]$$

Notably, $\Delta I$ and $I_{total}$ are not completely equal to $S_{1}$ and $S_{0}$ multiplied by some common factor (as was the case when time/transmission errors were considered in isolation), but retain components from both Stokes components. This admixture has the magnitude

$${d_{0^{\circ}}T}_{h}+d_{45^{\circ}}T_{v}-{d_{0^{\circ}}T}_{v}-{d_{45^{\circ}}T}_{h}$$

and therefore should be small. However, it remains the case that when both time instability and channel transmission inhomogeneity are present, the $S_{1}$ component estimated through $\Delta I$ contains a term which is linearly proportional to $S_{0}$ (*i.e*. a polarization artifact). Less importantly (as usually we have $S_{0}\gg S_{1}$), the $S_{0}$ component estimated through $I_{total}$ contains a term which is linearly proportional to $S_{1}.$

Following similar reasoning, an estimate for $\frac{S_{1}}{S_{0}}$ might be approached by calculating (similar to the g_lum_ calculation of Baguenard (*31*)):

$$\frac{\Delta I}{I_{total}}=\frac{\left( {d_{0^{\circ}}T}_{h}+d_{45^{\circ}}T_{v}+{d_{0^{\circ}}T}_{v}+{d_{45^{\circ}}T}_{h} \right)S_{1}+\left( {d_{0^{\circ}}T}_{h}+d_{45^{\circ}}T_{v}-{d_{0^{\circ}}T}_{v}-{d_{45^{\circ}}T}_{h} \right)S_{0}}{\left( {d_{0^{\circ}}T}_{h}+d_{45^{\circ}}T_{v}-{d_{0^{\circ}}T}_{v}-{d_{45^{\circ}}T}_{h} \right)S_{1}+\left( {d_{0^{\circ}}T}_{h}+d_{45^{\circ}}T_{v}+{d_{0^{\circ}}T}_{v}+{d_{45^{\circ}}T}_{h} \right)S_{0}}$$

Defining $\alpha=\frac{{d_{0^{\circ}}T}_{v}+{d_{45^{\circ}}T}_{h}}{{d_{0^{\circ}}T}_{h}+d_{45^{\circ}}T_{v}}$, this is simplified to

$$\frac{\Delta I}{I_{total}}=\frac{\left( 1+\alpha\right)S_{1}+(1-\alpha)S_{0}}{\left( 1-\alpha\right)S_{1}+(1+\alpha)S_{0}}$$

which only simplifies to $\frac{S_{1}}{S_{0}}$, if $\alpha=1$; this only holds, if $d_{0^{\circ}}=d_{45^{\circ}}$ or $T_{h}=T_{v}$, *i.e.* a combination of both time drift and channel transmission imbalance is not present.

On the other hand, we could simply note that

$$\frac{I_{v,0^{\circ}}\cdot I_{h,45^{\circ}}}{I_{h,0^{\circ}}\cdot I_{v,45^{\circ}}}=\left( \frac{S_{0}-S_{1}}{S_{0}+S_{1}} \right)^{2}$$

which gives

$$\frac{S_{1}}{S_{0}}=\frac{1-C}{1+C}$$

where $C=\sqrt{\frac{I_{v,0^{\circ}}\cdot I_{h,45^{\circ}}}{I_{h,0^{\circ}}\cdot I_{v,45^{\circ}}}}$ only contains directly measured quantities. Therefore, it is possible to extract out a fully error-corrected quantity $\frac{S_{1}}{S_{0}}$ even in the presence of combined time drift and channel transmission imbalance. Since $I_{total}$ should be a good approximation of $S_{0}$ in the limit $S_{0}\gg S_{1}$ and $\alpha=\frac{{d_{0^{\circ}}T}_{v}+{d_{45^{\circ}}T}_{h}}{{d_{0^{\circ}}T}_{h}+d_{45^{\circ}}T_{v}}\approx1$, we can then recover an approximation of $S_{1}$ as

$$S_{1}\approx I_{total}\frac{1-C}{1+C}$$

thereby recovering all the quantities of interest.

The difference between calculating $\frac{S_{3}}{S_{0}}$through $\frac{\Delta I}{I_{total}}$ and $\frac{1-C}{1+C}$ is illustrated in the figure below, where “channel avg.” refers to $\frac{\Delta I}{I_{total}}$ and “better error cancelling” to $\frac{1-C}{1+C}$. It can be seen that a very similar noise level and numerical value is obtained by either method.

*Figure S4: S_3_ measurement of r-BPC in toluene solution (excitation 343 nm, 200 fs, 12.5 kHz). The quantity* $\frac{S_{3}}{S_{0}}$ *in the bottom panel is calculated in two different ways with different approaches to error cancellation, as outlined in the text.*

**Section 3: Supplementary Data**

*Figure S5: UV-visible absorbance spectra of solutions used in this work.*

*Figure S6: Luminescence decay traces and fitted multi-exponential kinetics for non-polarization sensitive luminescence measurements of Eu[(+)-facam]_3_ in DMSO at various wavelengths corresponding to spectral features of interest (excitation 343 nm, 200 fs, 500 Hz).*

*Figure S7: Time-integrated (excitation 343 nm, 200 fs pulses, 50 kHz, gate pulse width 20 μs covering full time between pulses) CPL measurements of Eu[(+)-facam]_3_ in DMSO for a freshly prepared solution and after multiple hours of measurement time with pulsed 343 nm excitation, showing the decrease in CPL activity likely resulting from sample degradation.*

*Figure S8: Time-resolved CPL measurements of Eu[(+)-facam]_3_ in DMSO with lower excitation frequency (excitation 343 nm, 200 fs, 500 Hz) to minimize wraparound emission.*

*Figure S9: Steady-state measurements of Eu[(+)-facam]_3_ in DMSO with varying spectrograph slit widths. Note that as the slit size approaches the detector pixel size (13.5 μm) decreasing slit sizes becomes less effective for improving resolution.*

*Figure S10: S_1_ Stokes measurement of rhodamine B in aqueous solutions with horizontal and vertical excitation polarization (excitation 515 nm, 200 fs, 50 kHz).*

*Figure S11: S_2_ Stokes measurement of rhodamine B in aqueous solutions with horizontal and vertical excitation polarization (excitation 515 nm, 200 fs, 50 kHz).*

*Figure S12: S_3_ Stokes measurement of rhodamine B in aqueous solutions of different viscosity upon horizontal and vertical excitation polarization (excitation 515 nm, 200 fs, 50 kHz).*

While sucrose is chiral, the polarization spectra retaining the same shape but different intensities with or without sucrose (Figures S10-S12) suggests that the origin of the apparent CPL signal is not any inherent chirality of the system but an artifact induced by the presence of linear polarization. This is further strengthened by the observation that linear polarization of luminescence due to photoselection is much larger in magnitude when sucrose is present, likely due to the increased viscosity of the medium leading to slower orientational relaxation of dye molecules.

*Figure S13: Full-Stokes measurement of rhodamine B in aqueous solution with horizontal excitation polarization displaying low-noise baselines (excitation 343 nm, 200 fs, 50 kHz). There is a small S_1_ offset observed, which may be due to slight imperfections in the polarization state of the excitation beam or the excitation beam orientation slightly deviating from orthogonal w.r.t the collection axis.*

**

*Figure S14: Time-resolved CPL experiments of a chiral TADF-active dye, S-BPH, in toluene solution (excitation 343 nm, 200 fs, 12.5 kHz). Prompt’ refers to the first 100 ns, ‘Delayed’ to approximately 500 ns–80 μs, and ‘Total’ to a gate covering the complete emission process (0–80 μs). Note that prompt and total traces almost completely overlap in the top panel.*

*Figure S15: S_1_ Stokes measurement of camphorquinone (both enantiomers and racemate) in aqueous solutions with horizontal excitation polarization (excitation 405 nm, CW).*

*Figure S16: S_2_ Stokes measurement of camphorquinone (both enantiomers and racemate) in aqueous solutions with horizontal excitation polarization (excitation 405 nm, CW).*

**

*Figure S17: S_3_ Stokes measurement of camphorquinone (both enantiomers and racemate) in aqueous solutions with horizontal excitation polarization (excitation 405 nm, CW).*

*Figure S18: Nanosecond time-resolved non-polarized luminescence of Eu[(+)-facam]_3_ in DMSO solution (excitation 343 nm, 200 fs, 500 Hz) with varying time bins. Data as presented in Figure 3a of the main body, but without normalization to the peak value for each spectrum (values are normalized to the most intense pixel across all slices collected).*

*
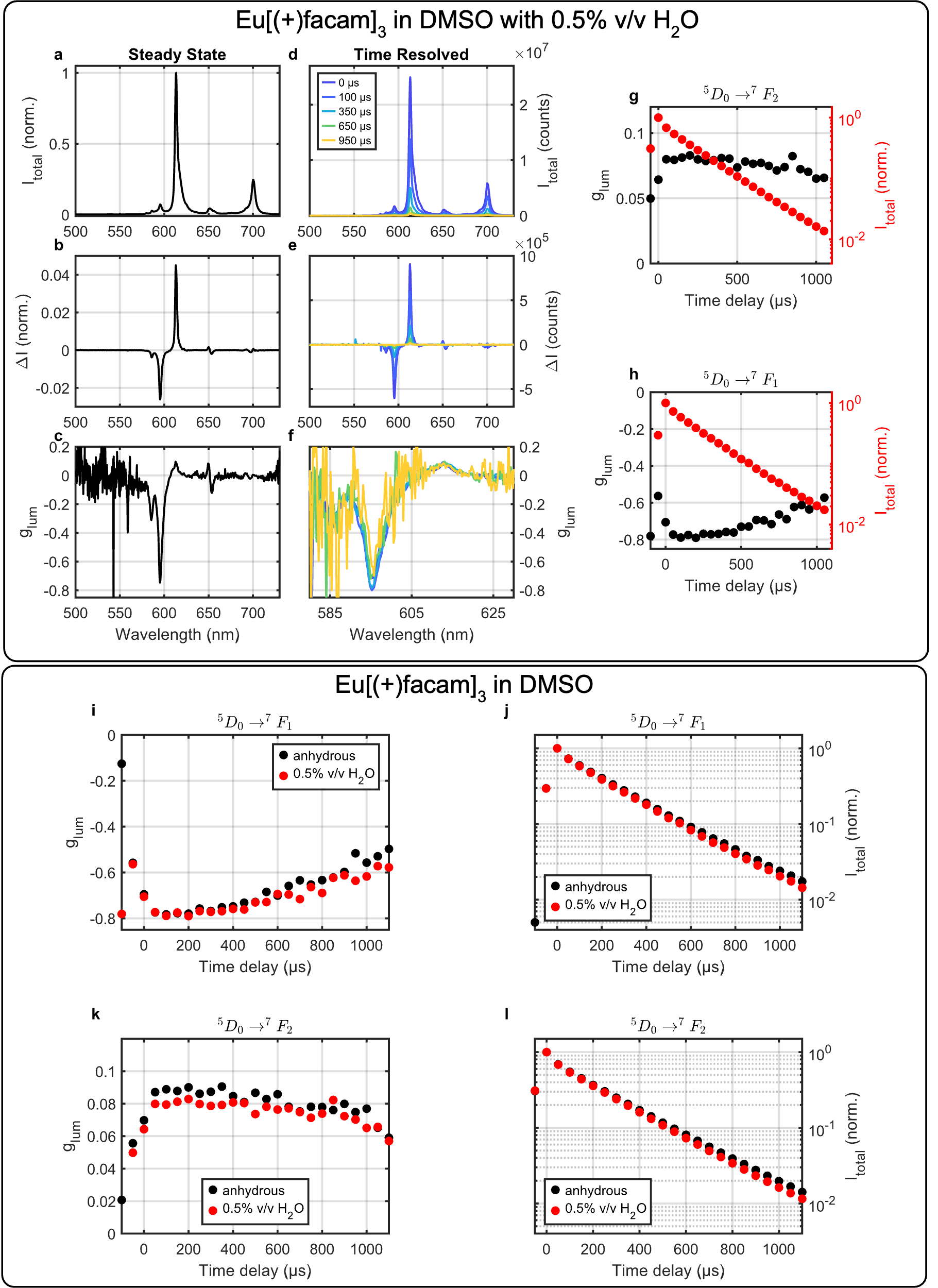
**Figure S19: Time-resolved CPL measurements of Eu[(+)-facam]_3_ in “wet” DMSO (with 0.5% v/v H_2_O added, as done by Hananel et al. (16)). Excitation 373 nm, 200 fs, 500 Hz, horizontally polarized.* ***a-c****,* *Time-averaged CPL measurement (2 ms time gate width).* ***d-f****, Time-resolved CPL measurement (50 μs time gate width and 50 μs gate steps) showing spectra at selected time slices.* ***g-h****,* *Intensity and dissymmetry factor as a function of time at the ^5^D_0_→^7^F_2_ (near 615 nm) and ^5^D_0_→^7^F_1_ (near 595 nm) luminescence peaks.* ***i-l****,* *Comparison of dissymmetry and total luminescence dynamics for Eu[(+)-facam]_3_ in “wet” DMSO and in anhydrous DMSO (measured with the same parameters and concentration).* *Compared to results obtained by Hananel et al., the time-evolution of dissymmetry in anhydrous DMSO is similar but the impact of water contamination is less apparent.*

*Figure S20: Steady-state CPL spectra of the chiral TADF-active compound BPC in toluene solution. Data for both enantiomers is shown. Spectra were acquired using a PEM-based CPL setup (excitation with 365 nm LED, continuous wave).*

*Figure S21: Steady-state CPL spectra of the chiral lanthanide complex Eu(facam)_3_ in DMSO solution. Spectra were acquired using a PEM-based CPL setup (excitation with 365 nm LED, continuous wave) and previously included in our publication describing that setup (19).*

**4: Practical Considerations for Measurements**

In this section, we briefly outline various effects which we believe constitute helpful background knowledge for readers seeking to perform TRCPL measurements, specifically with an approach like ours. Some items discussed are general and well-established in literature (such as photoselection), and some specific to our measurement scheme (such as optics imperfections).

**4.1: Photoselection Effects and How to Mitigate Them**

Photoselection arises from preferential excitation of dipoles along the electric field (polarization) axis of excitation light. It is important to consider in CPL measurements due to their sensitivity to linear anisotropy-induced artifacts, with photoselection being a common source of linear anisotropy even in otherwise isotropic samples, as well described by Blok and Dekkers (*19*).

Photoselection can never be truly eliminated, as the propagating light beam contains no electric field component parallel to its propagation direction for any polarization (including unpolarized light). Dipoles along this axis can therefore not be excited. However, mitigation strategies for eliminating preferential linear polarization in detected light exist. Blok and Dekkers (*19*) point out two particular configurations where artifact-free CPL values can be recorded: orthogonal excitation and collection (90⁰ geometry) with horizontally polarized excitation, and collinear excitation and collection (180⁰ geometry) with unpolarized excitation. We briefly discuss these, and the particular strengths of both configurations from a practical point of view.

*Photoselection with a 90⁰ Experimental Geometry*

Preferential orientation of dipoles along an axis parallel to the light collection axis will result in uniformly (un)polarized light at the collector. This is illustrated in Figure S22, and is the situation when the excitation polarization is horizontal and the collection axis is orthogonal to the excitation axis (*i.e.,* using the 90⁰ geometry).

On the other hand, using vertical polarization in the same 90⁰ geometry results in maximally polarized light along the collection axis. These two experiments therefore provide a convenient comparison for minimizing and intentionally maximizing photoselection effects in the collected light.


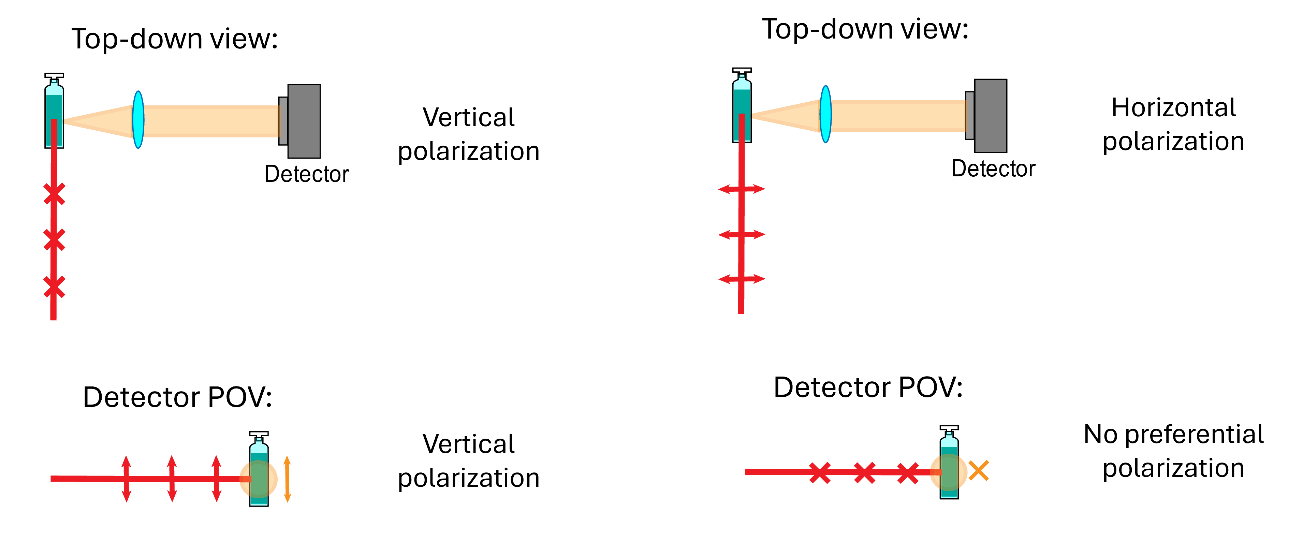


*Figure S22: Illustration of photoselection effects and resultant preferential luminescence polarization at the detector for a 90⁰ excitation-collection geometry. Cases with vertical and horizontal excitation polarization are depicted.*

A particular advantage of the 90⁰ geometry is that the avoidance of photoselection-induced preferential linear polarization in the collected light can be achieved by using a well-defined linear polarization state. This can be experimentally achieved straightforwardly and with high extinction ratio by a suitable polarizer. This is especially relevant for excitation sources such as ultrafast pulsed lasers which are strongly polarized at the source and for which depolarization to a sufficient degree may not be experimentally trivial.

Further, solution samples in square-based cuvettes can be oriented at right angles to excitation and collection, avoiding polarization-selective reflection angles and yielding a robust and straightforward methodology.

*Photoselection with a 180⁰ Experimental Geometry*

However, for samples such as thin films, mounting the samples to be normal to and homogeneous along the excitation-collection axes is often not possible. Polarization-selective reflections may become an issue when the excitation is incident at an offset angle. Further, emission intensity along an axis orthogonal to the excitation may be significantly reduced, especially in the limit of infinite viscosity and limited depolarization (*51*).

In such cases, the 180⁰ provides a valuable alternative for mitigating photoselection effects in collected luminescence. In this case, even a thin sample can be mounted orthogonal to the excitation and collection axes.

In a 180⁰ measurement geometry, both vertical and horizontal excitation polarizations will result in linear photoselection effects in the collected luminescence (illustrated in Figure S23). Instead, unpolarized excitation light is required to minimize polarization artifacts in CPL measurements.


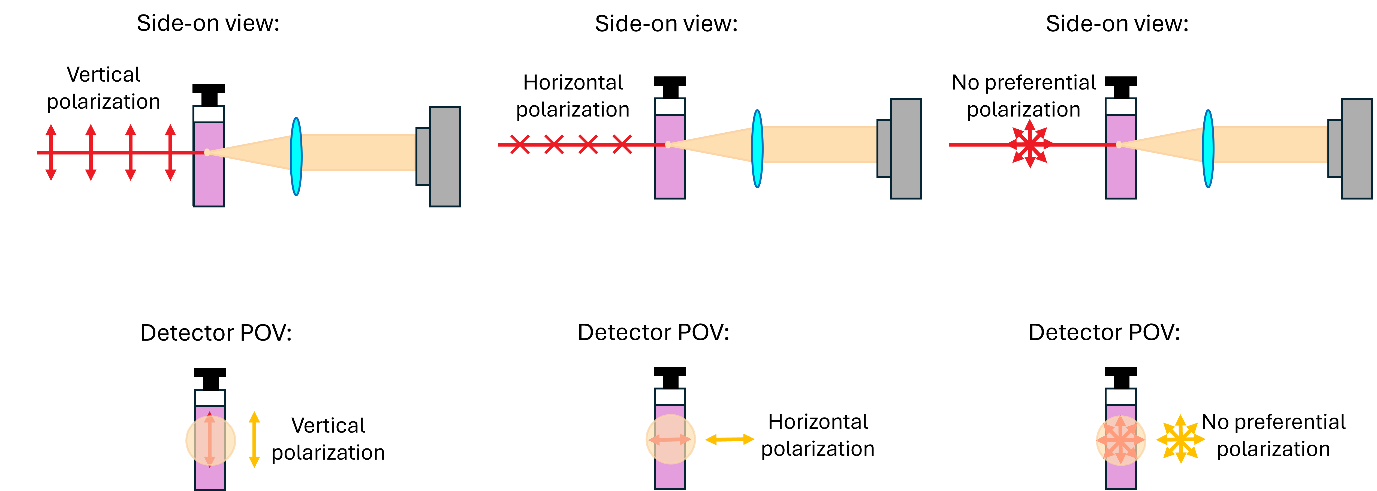


*Figure S23: Illustration of photoselection effects and resultant preferential luminescence polarization at the detector for a 180⁰ excitation-collection geometry. Cases with vertically polarized, horizontally polarized and unpolarized excitation are depicted.*

**4.2: Circular Dichroism-induced CPL Artifacts**

A well-known but easily overlooked source of erroneous CPL dissymmetry values is self-absorption in the presence of electronic circular dichroism (ECD). Corrections for such effects specifically for measurement geometries similar to ours can be derived (*52*). Similar effects are seen in solid films, which may display substantial effects from self-absorption with ECD (*53*).

In Figure S24, we briefly illustrate the effect when luminescence and the lowest-energy absorption feature have the same sign of dissymmetry (as expected when the same transition is involved in both features). For example: absorption and emission of right-handed light is preferred. In this case, right-handed light is preferentially emitted, but preferential self-absorption of the right-handed component in luminescence will act to reduce the magnitude of CPL measured. If sufficient self-absorption occurs, a bisignate CPL spectrum could result.


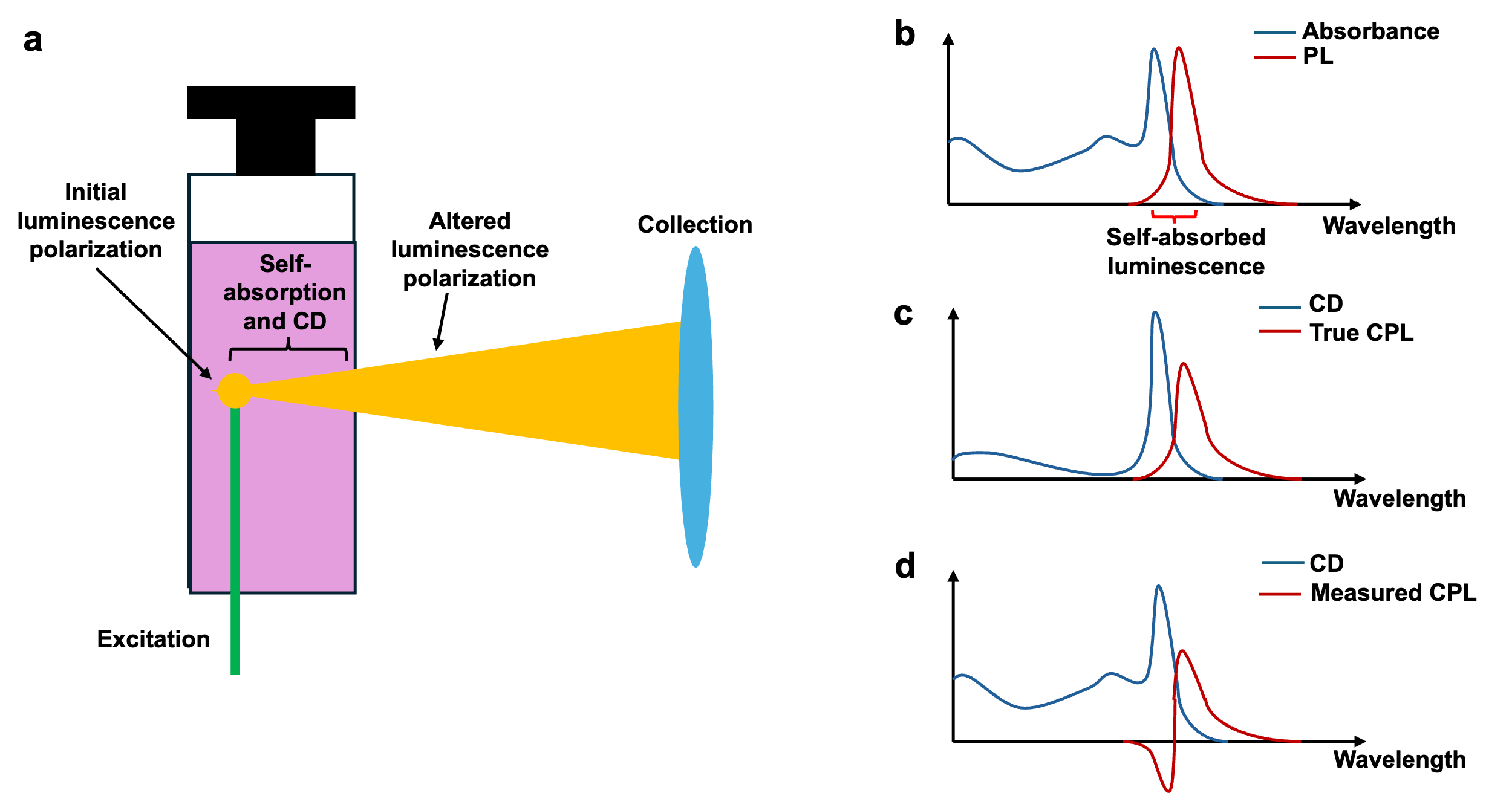


*Figure S24: Illustration of CPL artifacts induced by self-absorption of a sample exhibiting circular dichroism.* ***a,*** *Schematic of how collected luminescence may undergo self-absorption in a typical measurement configuration* ***b,*** *Illustration of spectrally overlapping absorbance and photoluminescence (PL), leading to self-absorption in the overlap region* ***c-d,*** *Illustration of how “true” CPL (measured in the limit of no self-absorption) may differ from measured CPL in the presence of significant self-absorption in a sample exhibiting circular dichroism (CD).*

For a solution sample in a thick cuvette measured in the 90⁰ geometry, one could in principle adjust the degree of self-absorption by controlling the position of the sample relative to the excitation beam, as illustrated in Figure S25. While this may not fully remove the artifact directly, it can at least act as a “sanity check” to easily determine whether self-absorption is influencing the measured CPL values significantly. Alternatively, a concentration series for a solution sample can also provide information about self-absorption effects, if measured dissymmetries are found to be concentration-dependent.


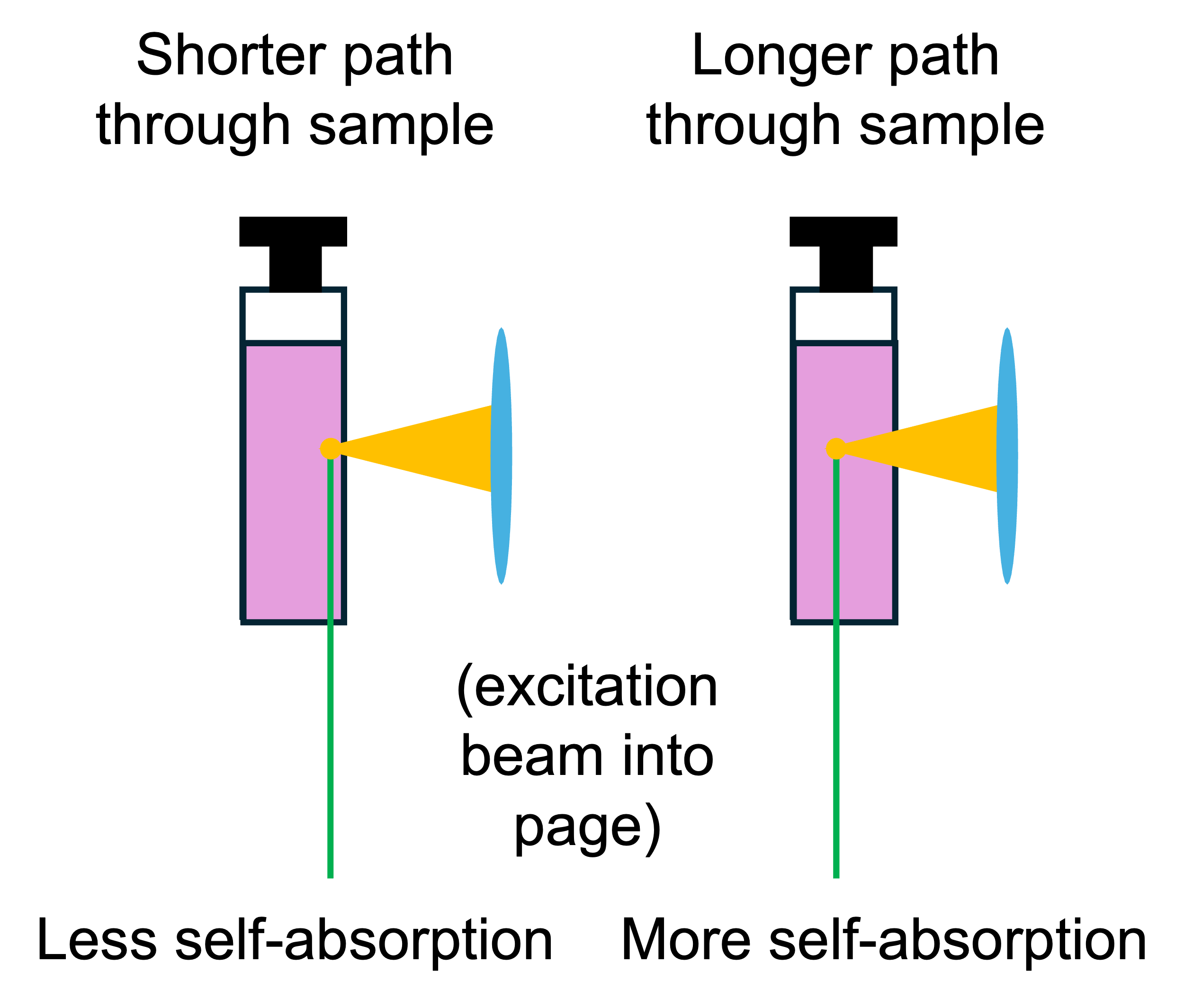


*Figure S25: Illustration of how the degree of self-absorption for luminescence collected from a sample can be varied straightforwardly in a 90⁰ excitation-collection geometry.*

**4.3: Signal-to-Noise and Acquisition Time in TCSPC-based Time-Resolved CPL**

We briefly explain here, why TCSPC-based approaches, such as described by Schauerte *et al.* (*13*) face issues with impractically long collection times.

At the most fundamental level, TCSPC signal acquisition rate is limited by the need to avoid photon pile-up effects. Noise in single photon counting measurements is well-described by Poisson statistics, where the signal-to-noise ratio (S/N) scales as $\sqrt{N}$ for $N$ photons counted. For noise levels on the order of 10^-4^ (desirable for accurate measurement of typical g_lum_ of about 10^-3^), this requires counting 10^8^ photons. Typically, TCSPC measurements are carried out with photon detection rate not exceeding $0.05f$ where $f$ is the sync signal frequency (*i.e.* excitation source repetition rate) to avoid distorting kinetics through photon pile-up. It follows that achieving the 10^-4^ noise level *via* TCSPC requires measuring for $\frac{2\times{10}^{9}}{f}$ seconds, practically necessitating MHz repetition rate excitation; even for a 1 MHz excitation, this gives a collection time of over half an hour. The problem is made worse when one takes into account that the photons are further distributed over multiple time bins, with most of the photons falling into the earlier bins. Achieving high S/N in the later time bins is therefore prohibitively time-consuming. As TCSPC detectors are single-element detectors, building up a spectrum also necessitates stepping through wavelengths one at a time, multiplying the required measurement time.

To mitigate issues, excitation sources with repetition rates on the order of 10 MHz could be used, and multiple counting channels can be used. However, even at 1 MHz the excitation pulse spacing is only 1 μs, insufficient for many materials emitting *via* spin- or symmetry-forbidden transitions (*e.g.* chiral lanthanide complexes (*28*)).

**4.4: Signal-to-Noise and Acquisition Time in CCD-based Time-Resolved CPL**

As discussed in Section 4.3, for accurate CPL measurements a large number of photons at each wavelength must be counted. In practice, the Poisson noise approximation will be an underestimate for a CCD-based setup, as the intensified CCD detector has additional noise contributions compared to a photon-counting system (readout noise, shot noise in the dark signal, shot noise in the signal) which may also vary with acquisition parameters. However, Poisson noise provides a convenient back-of-the-envelope estimate of the requisite signal strength to achieve a given level of precision.

Compared to a TCSPC-based system, the lack of pileup effects and large number of simultaneously measured wavelength channels in a CCD-based setup allows for significantly faster data acquisition. Further, if full kinetics are not desired, it is possible to measure a single time slice at an arbitrary time range which is significantly faster.

We find that measuring such a large number of photons can still be relatively time-consuming, owing to limits on detector readout rate, laser repetition rate and detector saturation effects. Depending very heavily on the type of sample measured, measurement times for useable data range from seconds (lanthanide complex in the steady state) to hours (time-resolved kinetics of a chiral organic molecule). Low luminescence intensity (dim samples or short time slices of long-lived luminescent species) will further increase measurement duration. It is therefore important to properly define measurement settings for sensible measurement durations.

For faster measurements, we want to maximize the number of charges collected and read out per second. This is most straightforwardly controlled by excitation power (per pulse), but may eventually cause degradation or other issues. Increasing the excitation laser (and thus gate pulse) repetition rate will increase signal strength, but normally we want to avoid wrap-around emission (*i.e*. emission that leaks into the photon collection after the next pulse) if possible. Beyond this, appropriate choice of time gates, exposure time and accumulations are important. Where appropriate, pixel binning is also a possible approach. For our purposes, we normally bin vertical pixels such that only two tracks are recorded and read out, which greatly increases detector readout rate compared to a full-sensor readout and reduces readout noise. In particular, increasing the intensifier gain will also amplify noise, and therefore should be the “last resort” parameter to increase count numbers.

**4.5: Overexposure and Phosphor Glow**

While the gated iCCD detector does not suffer from photon pile-up in the same way as TCSPC measurements do, the detector will eventually saturate (pixels have a finite well depth), which together with the maximum readout speed and minimum exposure time provides a hard upper limit for data acquisition rate. Additionally, inherent to the iCCD device are a photocathode and phosphor screen for converting incident photons into electrons for amplification and gating and back to photons for detection at the CCD. Overexposing the phosphor will result in afterglow fading slowly over minutes to hours. Though this might affect both channels similarly, and normally would be a small effect compared to the real luminescence intensity, we nevertheless advise caution. Practically, we have found that phosphor overexposure is a concern before well depth, when increasing incident light intensity.

**4.6: Slit Width**

Slit width introduces a tradeoff between intensity and resolution. For a narrow-line emission like that of Eu^3+^, we recommend narrow (10-50 μm) slit widths to avoid smearing out features. This effect is shown in Figure S9, where the dissymmetry values for Eu(facam)_3_ are shown to vary with slit width. For a pixel array detector, the pixel size usually introduces the lower limit to which slit size can improve resolution. Finer gratings allow for greater resolution at the cost of narrower bandpass (and, for holographic gratings, greater polarization sensitivity).

For broader spectral shapes, such as the organic molecules investigated here, a wider (100-200 μm) slit allows for more light in-coupling, which is advantageous.

**4.7: Sample Degradation**

As sample excitation occurs through a pulsed laser (and often a pulsed UV laser), and accurate CPL requires large numbers of counted photons meaning high intensities or long measurements, sample degradation is a concern. Some degradation was observed in Eu(facam)_3_, illustrated in g_lum_ values being lower after prolonged laser exposure in Figure S7. To mitigate this, sample exposure should be minimized and fresh samples used. A flow cell might also be appropriate for sensitive applications, and stirring solutions is advisable.

**4.8: Wollaston prism: Deflection angle wavelength dependence**

Compared to standard polarizing beamsplitter cubes or plates, Wollaston prisms can offer superior performance in terms of extinction ratio and polarization splitting over a wide wavelength range. High extinction ratios are crucial for achieving sensitive polarization measurements, and broadband response is necessary for simultaneous acquisition over a wide spectral range. In our measurement scheme, both output beams exiting from the same face at symmetric deviation angles is also advantageous for a splitting/collection approach minimizing the amount of optical components necessary.

However, the deflection angle of Wollaston prisms is wavelength-dependent in general, and it has been suggested that this could necessitate complex workarounds in applications like ours (*31*). To address such concerns, we briefly present data showing that a suitable choice of components will result in the wavelength-dependence being miniscule.

For the Wollaston prism used (WPQ10, Thorlabs), the manufacturer provides data of the variation in deflection with wavelength. Over the full span of the approximate operation range of our instrument (ca. 400-900 nm) this deviation is less than 0.1⁰.

To demonstrate the effect on our setup specifically, we refer to Figure S26, which displays a full-sensor image of rhodamine B luminescence (horizontally zoomed to the luminescence region) showing the two polarization tracks. To help visualize possible line shifts in the vertical sensor direction, we have also plotted lines showing vertical positions of peak counts at each wavelength.

The measurement in question shows a variation in peak y-position of approximately 1-2 px over 150 nm, which is small compared to the sensor size (512 px), track separation (~150 px) and vertical peak width (~10 px).

During actual measurements, vertical binning is performed over two regions which are ~200 px in height (the top and bottom halves of the sensor, approximately). The full line is therefore collected regardless of small variations in vertical position.

In summary, the variation in angular deviation is small to the point of being barely detectable, and especially with vertical binning over a much larger pixel range is unlikely to result in significant changes of the collected signal. In particular, Baguenard *et al.* found their CCD-based CPL measurements insensitive to much larger tilt misalignments (31).

**
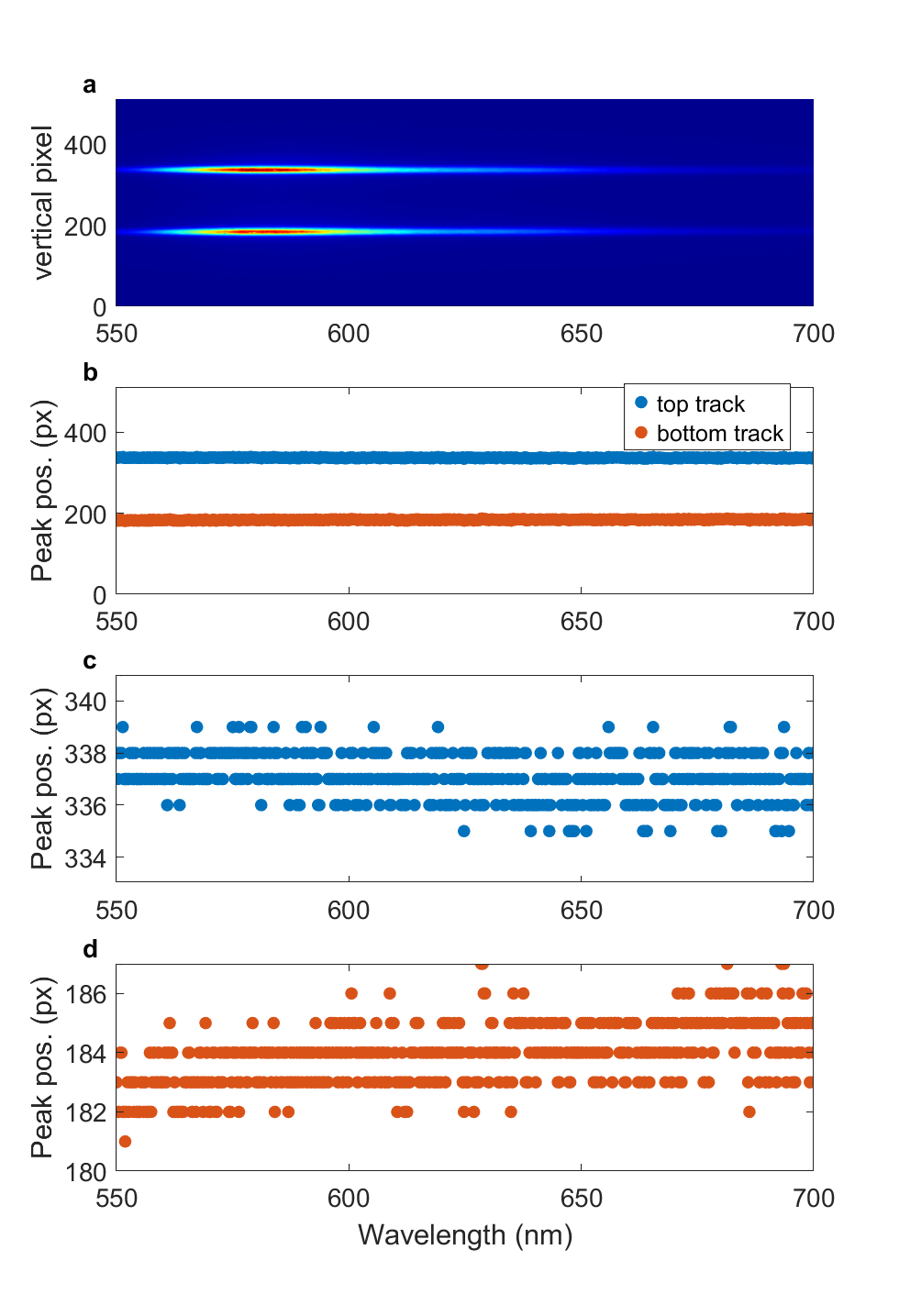
**

*Figure S26:* ***a****, Full-sensor image (horizontally cropped to the luminescence region) showing orthogonally polarized tracks (rhodamine B in water, 343 nm excitation, vertical ex. polarization).* ***b****, Vertical pixel peak positions for the upper and lower tracks.* ***c****/****d****, Zoomed-in traces of vertical peak positions for the top/bottom tracks, respectively.*

**4.9: Detector Pixel Non-uniformity**

Individual pixels in a CCD detector generally exhibit variations in their response characteristics. The result is fixed pattern noise, that is, differences in count values read out by individual pixels when an identical amount of light is incident. This will be partially due to per-pixel variation of the dark signal, which will be temperature-dependent but otherwise fixed, and partially due to per-pixel variation in sensitivity (on the order of approximately 1%) due to manufacturing irregularities. The dark signal variation can be removed by background subtraction and, in principle, the per-pixel sensitivity variation, like other channel-dependent sensitivity variations, should be corrected for (in the difference spectra and dissymmetry factor) by the two-step measurement procedure, where a QWP rotation flips the horizontal/vertical channels. However, as discussed in the following section, this is not always the case.

**4.10: Beam Deviation and Waveplate Rotation**

For the waveplates used (Thorlabs, QWP: SAQWP05M-700 and HWP: SAHWP05M-700) the manufacturer specifies a beam deviation of < 3 arcmin. We have found the extent of deviation to vary somewhat between individual waveplates. Though the deviation tolerances are quite small, rotating the waveplate causes the transmitted beam to trace a circle. This may cause noticeable effects at collection optics, with some dependence on experimental parameters like slit width, excitation geometry, etc. While good results may be achievable without addressing waveplate deviation explicitly in fortuitous cases, for reproducible performance across different measurements awareness of such effects is important.

For simplicity, we split the deviation effects into horizontal and vertical deviation, as illustrated in Figure S27. These predominantly affect different parts of detection, with horizontal deviation mainly an issue at the spectrograph entrance slit and vertical deviation mainly an issue at the CCD.

Horizontal deviation may cause partial clipping of the beams going into both tracks, as the spectrograph in-coupling slit is small and vertically oriented. This will lead to a change in recorded intensity as the waveplate is rotated. We have found that this intensity change is not always fully cancelled out. We suspect this is because the assumption for error-cancelling is that any “time instability” error sources affect both channels in an identical fashion, which is not necessarily the case, as the clipping at the slit happens after the polarization components are separated into two beams. The result is generally a baseline offset for dissymmetry measurements, for example, an achiral sample showing a CPL artifact with g_lum_ on the order of 10^-3^ (see Figure S28 for rhodamine B). This effect can, however, be mitigated.

Vertical deviation, on the other hand, causes the pixels which collect light in the second measurement (waveplate orientation) to not be exactly the same pixels as in the first measurement. Due to this, the assumption for error-cancelling (that any “channel mismatch” error sources are constant over subsequent measurements) of pixel sensitivity variations is not met, resulting in fixed-pattern noise (*i.e.* consistent between measurements) approximately at the 10^-2^ level, which does not improve on further data accumulation. This is illustrated in Figure S28, where noise is consistently present in the same pixels even in separate measurements with different horizontal pixel binning values. This can also be mitigated.


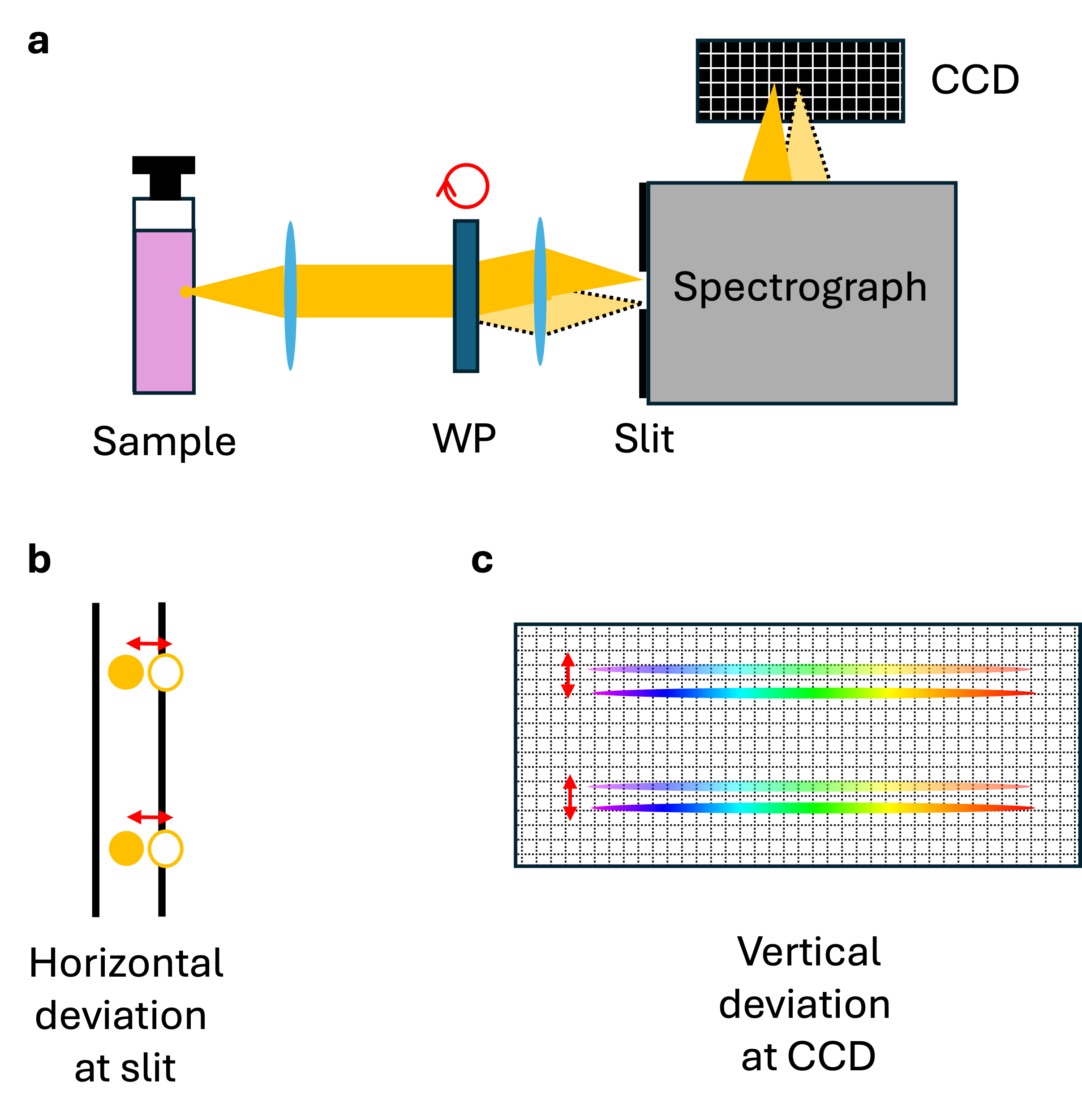


*Figure S27: Simplified illustration of the effects of waveplate deviation, which causes the beam to slightly move upon waveplate rotation. At the slit, this may result in clipping of the beam, if horizontal movement occurs. At the CCD array, this may result in different pixels collecting the signal, if vertical movement occurs, which will interfere with averaging out channel transmission characteristics.*

*Figure S28: CPL measurement of rhodamine B in aqueous solution (excitation 515 nm, 200 fs, 50 kHz, horizontal polarization) with various horizontal pixel binning values. This data shows two effects of beam deviation during waveplate rotation. First, a baseline offset (CPL artifact) due to channel-dependent intensity variations from horizontal beam deviation. Second, a higher-than-expected non-random noise background arising from incomplete cancellation of fixed-pattern noise due to vertical beam deviation. Both effects can be mitigated, as outlined in the text.*

While the primary error-cancellation procedure calls for measuring over two waveplate orientations (out of 4/8 possible ones for the QWP/HWP, respectively), using further waveplate orientations (calculation described in SI Section 2.1) can alleviate beam deviation effects. This is illustrated for horizontal deviation offsets in Figure S29. Depending on the waveplate angle pair chosen, the CPL offset changes sign but approximately retains its magnitude. We believe this to arise from the deviated beam tracing a circle over full rotation of the waveplate, causing a symmetrical effect on the channel intensities for the second pair of angles. Consequently, using data from all four QWP angles results in near-complete elimination of the baseline offset.


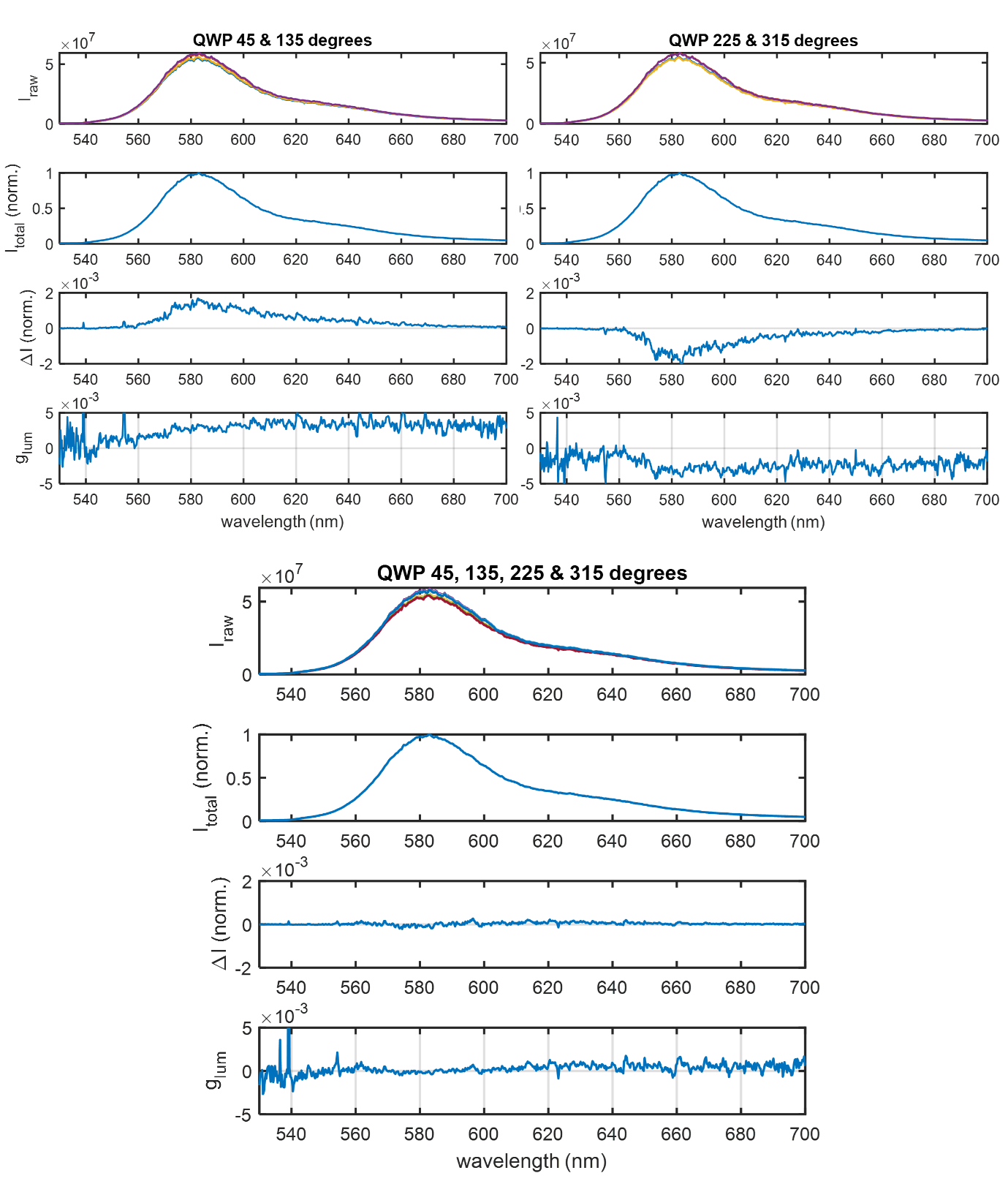


*Figure S29: CPL measurement of rhodamine B in aqueous solution (excitation 515 nm, 200 fs, 50 kHz) using various quarter-wave plate (QWP) angle combinations. Due to horizontal beam deviation, a small baseline offset artifact is observed when using two QWP angles for error-cancellation, with the sign depending on the angle pair chosen. Measuring over all four QWP angles cancels out this effect, resulting in a clean baseline.*

Horizontal beam deflection can also be mitigated by using a 90⁰ excitation-collection geometry with a slow excitation focusing lens, such that the excitation beam forms a line through the sample.

Besides using multiple waveplate orientations, vertical deviation effects can also be corrected for by the somewhat crude but effective method of steering the excitation beam vertically after QWP rotation such that the same pixels are used to collect signal in both measurements. This is relatively simple, as the detector has a live imaging readout, making alignment straightforward. However, this relies on the sample being homogeneous (valid for solutions). Some amount of transmission change from beam steering is unavoidable, although this was found to work sufficiently well for high-accuracy measurements in our case.

Manual beam resteering is illustrated in Figure S30, and the result on measurement “noise” in Figure S31. For exactly the same measurement parameters, the apparent noise level is massively improved by manually resteering the beam after waveplate rotation. This is because the “noise” without beam resteering is not true statistical noise, but rather static imperfections in the error cancellation caused by changing the pixels over which data is collected.

While these approaches allow for control over deviation-induced effects sufficiently to perform high-accuracy polarization measurements, it would be preferable to eliminate waveplate deviation entirely in the future. Besides higher-spec waveplates, approaches such as deviation-compensating optical elements are a potential way to manage the effect directly at the cost of additional optical elements.

Besides mitigating beam deflection by optics, a possibly elegant and automatable approach to mitigate pixel sensitivity is the scanning multichannel approach. Here, instead of doing a single series of accumulations at a given grating position, a smaller number of accumulations is performed at multiple, slightly offset grating positions (*54*). This approach has been successfully used to mitigate pixel noise in, for example, femtosecond stimulated Raman spectroscopy (*55*). As an added benefit, this will similarly smooth out any larger-scale sensitivity variations of the intensifier/detector, which normally have slightly lower sensitivity near the edge regions, without requiring a manual calibration file. However, transmission characteristics of optics will still contribute to spectral shape and require calibration to recover the original spectrum.


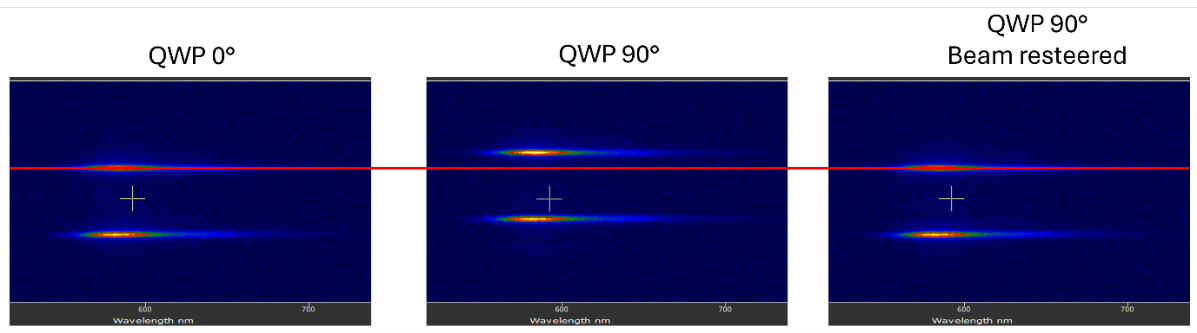


*Figure S30: Illustration of vertical beam drift upon waveplate rotation. The spectral traces are translated vertically to different pixels when the beam drifts, resulting in incomplete error cancellation. By manually resteering the beam between measurements (done using the steering mirror immediately before the sample) this can be reversed. Images manipulated to exaggerate the effect for clarity.*

*Figure S31: CPL measurement of rhodamine B in aqueous solution (excitation 343 nm, 200 fs, 50 kHz). Vertically polarized excitation results in a photoselection-induced CPL artifact, as discussed in the main body, resulting in apparent CPL for this achiral dye. Data showing such an artifact was measured with and without manual resteering of the beam between waveplate rotations, with otherwise exactly the same parameters. Resteering greatly reduces the apparent noise level, but preserves most of the shape and intensity of the CPL artifact. For a horizontal excitation polarization, no CPL artifact is present, and a low-noise baseline is measured with beam resteering.*

**5: Algorithms and Automation**

This section details our proposed algorithms that enable systematic acquisition of time- and polarization-resolved data.

Automation brings numerous advantages, including ensuring reliability during measurements, saving time for performing measurements and improving reproducibility of results. Our key motivation behind automation is to eliminate potential inaccuracies introduced by manual operations and to enhance user comfort. A measurement algorithm was developed for the given hardware configuration for time- and polarization-resolved measurements. The developed procedure is essential for automatically capturing data with our setup, specifically the g_lum_ and the full Stokes vector. For polarization-resolved measurements, the angle of the QWP and HWP are systematically varied, and measurements are accumulated. Time- and polarization-resolved measurements extend this approach to include time gating for capturing time-dependent information.

For the automated determination of the time-resolved Stokes vector in our constructed setup we propose a Time- and Polarization-Resolved Algorithm (TPRO). Pseudocode is provided in Algorithm 1, detailing the steps involved in capturing measurements under various conditions.


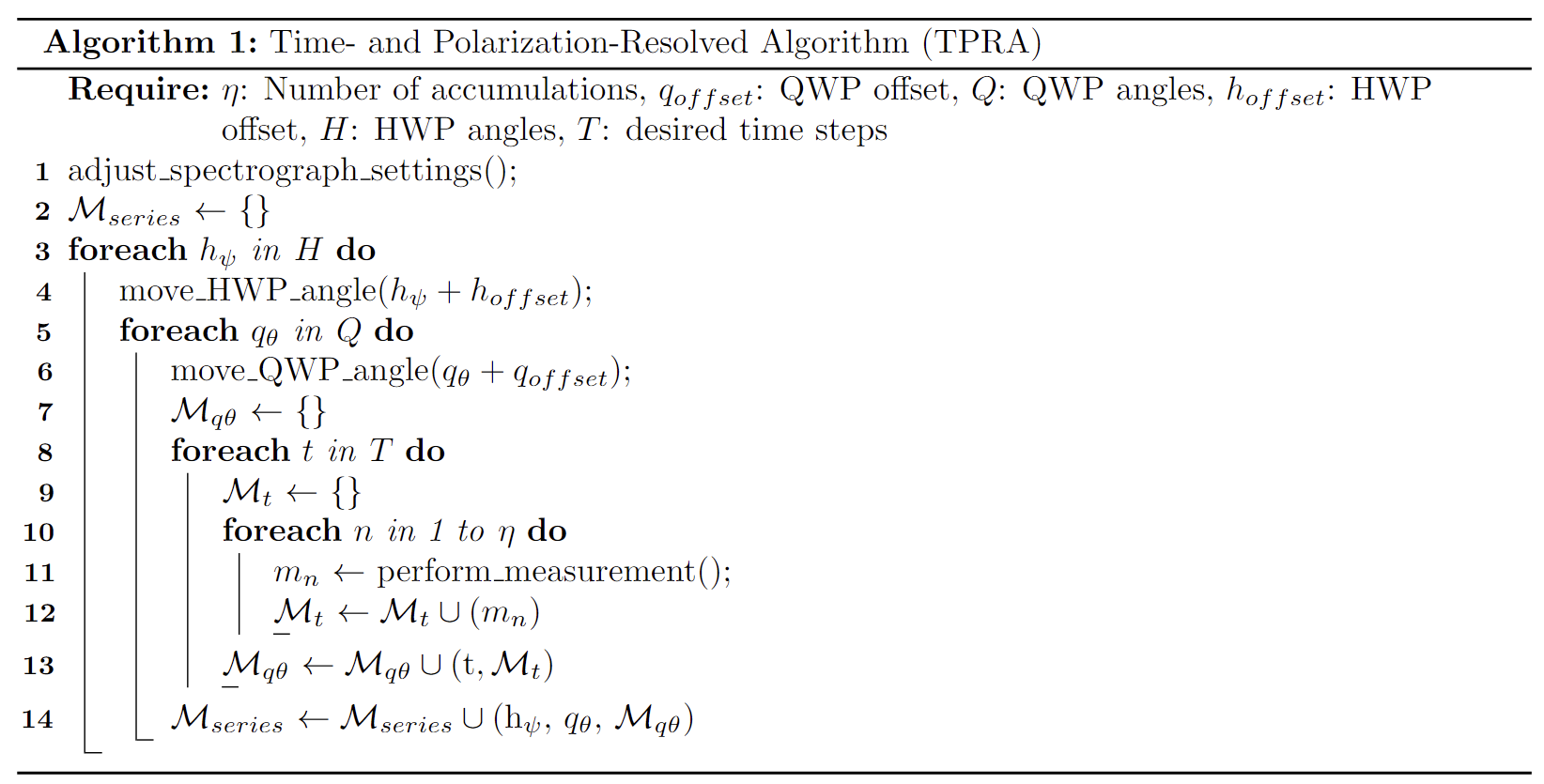


In total, six measurements are required which together yield the static Stokes vector. First, the measurement of *S*_1_ is carried out with the QWP fixed at 0˚, while the HWP is set to 0˚ and 45˚. Second, the measurement of *S*_2_ is performed with the QWP at 0˚, and the HWP rotated to 22.5˚ and 67.5˚. Third, the measurement of *S*_3_ is performed with the HWP set at 0˚, along with the QWP orientations at 45˚ and 135˚. Executing these measurements effectively switches the polarization tracks and minimizes the contributions from other polarizations, achieving the desired results, as explained in detail above. The procedure of stepping the QWP and HWP is described in lines 3-6. To extend the steady-state Stokes vector to the time domain, additionally, each of the six measurements must be performed at different time steps (line 8). These time steps are defined in terms of gate width and delay. The functionality and parameterization of gate width and delay have been explained in the main part. Finally, the loop in line 10 enables redundant measurements for each tuple (comprised of space and time) to minimize random errors based on accumulation.

Algorithm 1 can be extended by employing scanning multichannel detection (*54*) to improve the robustness against artifacts, namely, to reduce pixel sensitivity variations on the iCCD camera, as pointed out above. This extension is depicted as pseudocode in Algorithm 2. To add scanning multichannel detection, each measurement *n* of the total number of accumulations *N* (line 10) is extended by g sub-measurements with slightly different grating positions (line 12).


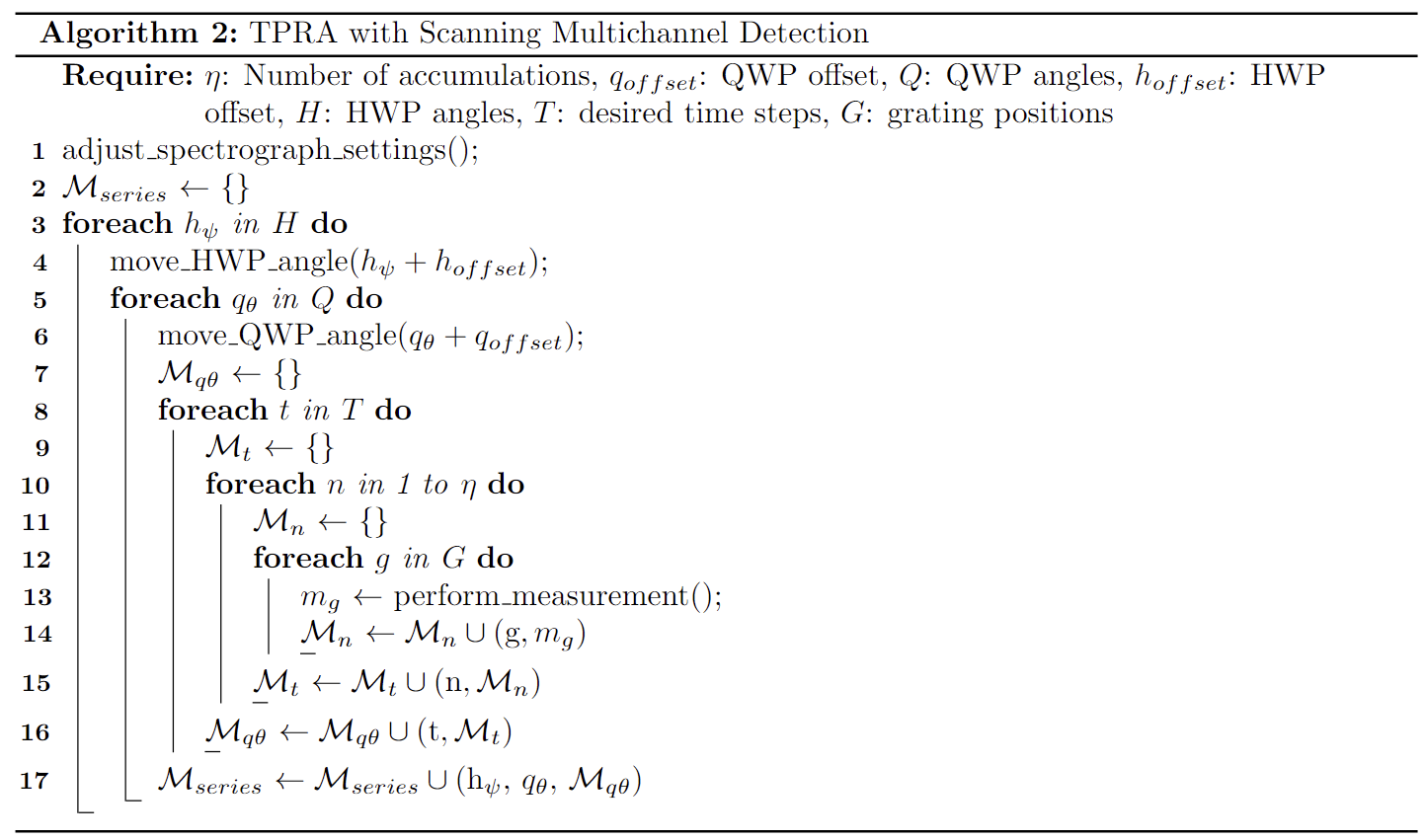


To perform the automated measurements on the real system, the proposed pseudocode in Algorithm 1 was implemented using Python. For hardware control, the code leverages different software plugins. This pseudocode is generic and system-agnostic, and concrete implementations can be realized relatively straightforward for different hardware and programming languages.

In the future, we seek to integrate our existing code into an open-source framework, to make it as open-source as possible. A promising framework could be PyMoDAQ (*56*). It has a large and active community that continuously develops modular plugins for GUIs and hardware for a wide range of instruments, enabling standardized implementations across different experimental setups. This can facilitate the comparison and reproducibility of results.

**Supplementary References**

51. A. H. Kalantar, Consequences of Photoselection on the Intensity and Polarization of Luminescent Molecules. *The Journal of Chemical Physics* **48**, 4992–4996 (1968).

52. G. Longhi, E. Castiglioni, J. Koshoubu, G. Mazzeo, S. Abbate, Circularly Polarized Luminescence: A Review of Experimental and Theoretical Aspects: CPL: EXPERIMENTS AND THEORY. A REVIEW. *Chirality* **28**, 696–707 (2016).

53. G. Albano, G. Pescitelli, L. Di Bari, Chiroptical Properties in Thin Films of π-Conjugated Systems. *Chem. Rev.* **120**, 10145–10243 (2020).

54. P. Knoll, R. Singer, W. Kiefer, Improving Spectroscopic Techniques by a Scanning Multichannel Method. *Appl Spectrosc* **44**, 776–782 (1990).

55. J. R. Challa, Y. Du, D. W. McCamant, Femtosecond Stimulated Raman Spectroscopy Using a Scanning Multichannel Technique. *Appl Spectrosc* **66**, 227–232 (2012).

56. S. J. Weber, PyMoDAQ: An open-source Python-based software for modular data acquisition. *Review of Scientific Instruments* **92**, 045104 (2021).
