# Supplementary material for: Broadband transient full-Stokes luminescence spectroscopy
Source: Nature. 2025 Jun 25;643(8072):675–82. doi: 10.1038/s41586-025-09197-3 (PMC12267050; doi:10.1038/s41586-025-09197-3)
Supplement: Supplementary file 2 — Peer Review File [file 41586_2025_9197_MOESM2_ESM.pdf]

nature portfolio

Peer Review File

Broadband Transient Full-Stokes Luminescence Spectroscopy

Corresponding Author: Professor Sascha Feldmann

This file contains all reviewer reports in order by version, followed by all author rebuttals in order by version.

Version 0:

Reviewer comments:

Referee #1

(Remarks to the Author)
This manuscript describes about "novel approach to achieve high-sensitive, broadband, time-resolved full-Stokes spectral measurements". The proposed approach is original and extremely significant in this research field. The results shown in the current manuscript are high quality and the paper is also well organized. I recommend the publication of this paper in Nature after a minor revision as follows,

1) It is known that glum value is significantly affected by sample temperature (e.g. Front. Chem., 2020, 8, 527). However, no information about the measurement temperature was shown in this manuscript. The authors should show all measurement temperatures.
2) In the insets of the Figures 2a and 2d, the way to show the Y-axis is not same ("x 1000" in Figure 2a and "x 104" in Figure 2d). The authors should standardize the format.
3) Please change "Hananel" to "Hananel et al." (line 26 in page 5, line 14 in page 7).
4) Please change "artefacts" to "artifacts" (line 24 in page 11).

Referee #2

(Remarks to the Author)
This manuscript describes a very valuable contribution to the field of time-resolved circularly polarized luminescence (TRCPL), which is a topic I very warmly welcome and that I believe will have great prospects. I would welcome its publication, possibly following the suggestions that I provide below, labelled in curly brackets. Please notice that especially point {c} and {d} are only suggestions! Overall, the manuscript is clear, with a well introduced contex, and it may call the attention of a wide readership.
The paper describes in detail the experimental setup that was used to achieve TRCPL, in such a way that it can inspire other groups in building analogue instrumentation. One or a few pictures of the actual setup may further be welcome in the SI, accompanying Fig. S1.{a} Noteworthy the proposed instrument allows for full Stokes polarization elements TR-measurements and for this reason it may extend beyond the realm of chirality and allow for fluorescence anisotropy depolarization measurements at the same time.
As examples of applications, the paper discusses a commercial standard, Eu(facam)3, whose TR-CPL had been reported in reference [22], and TADF molecule. In addition, rhodamine in different viscosity solutions is used to challenge the instrumentation for photoselection artefacts.
Polarization measurements and especially CPL may suffer from several artefacts, which are well known in the literature, but are nicely recalled and discussed in the SI. One aspect, which is not treated here, but which may be useful to mention are the artefacts arising from differential extinction (when ECD and CPL bands partially overlap). It should be straightforward to briefly discuss this aspect, as well, on account of its occurrence in most cases where organic fluorophores are involved and too often neglected. {b}
I believe that the detailed analysis of the transient processes occurring in Eu(facam)3 is of utmost interest and that it nicely complements Markovich et al.'s work of ref. [22]. To be even more thorough, it would be instructive to reproduce their data with wet DMSO, as well. {c?}
Concerning the decay of Eu(facam)3 CPL, there is one aspect that may be worth discussing here or in the future. The D0-F1 band around 595 nm, is not a single transition, as well known and seen even here. This is due to MJ splitting and I am not

sure that these components must decay at the same rate. This would account for non-constant glum and even for biexponential behaviour. I should very much appreciate the authors' comment, if they can (I understand that this may fall out of their expertise). {d?}
Concerning photoselection, besides horizontally polarized excitation, there is 180° geometry (in-line), as shown in Figure 1. This is a powerful means to cancel this very nasty artefact, but it seems to me that is not discussed here. From my own experience, I know that it may be extremely difficult to cope with the stray light of the UV-source, but I should be curious to hear the authors' experience. I should also like them to recall that in the limit of infinite viscosity (no molecular tumbling), horizontal excitation should lead to *no signal*. This often reduces the practical utility of this expedient. {e}
Lorenzo Di Bari

Referee #3

(Remarks to the Author)
The work demonstrates a new design for a CPL spectrometer using a single CCD spectrometer to collect both left and right circularly polarised light simultaneously by separating using rotating waveplates and a Wollaston prism.

The work is not novel enough and of broad interest to be published in Nature. The work is a stepwise improvement from the controversial work published by Baguenard et al in Nature Communications in 2023 where they achieved separating left and right circularly polarised light with a polarising beam splitter and then recombined light from fibre optics into a single CCD. This work does not represent a significant enough improvement on that work to warrant publishing in Nature.

There are also more specific additional concerns with the work:
1. The work does not show a comparison to conventional scanning monochromator-PEM based CPL spectrometers. This is essential to show that spectral form in ΔI is maintained on the same sample. Comparing glum only is not enough to validate a CPL spectrometer, particularly when comparing lanthanide complexes as done in this work, as they can have unique sign inversions within individual transitions.
2. The use of a motorised rotating mount to rotate the wave plates within the work introduces significant error that does not seem to be accounted for. The manufacturer claims the mount has a repeatability of ±0.1° which at first glance seems negligible but on repeated rotations the mount can drift and introduce error.
3. The Wollaston prism used does not have even deflection across all wavelengths, is this accounted for?
4. Figure S21 suggests that there is an inherent bias in the spectrometer that is quite significant for a non CPL active emitter.

Version 1:

Reviewer comments:

Referee #1

(Remarks to the Author)
I have checked the revised version of manuscript, SI, and response letter. The authors have accurately addressed the issues pointed out by all referees. In detail, the authors clearly showed all experimental temperatures during measurements. According to the previous reports about temperature dependance of g_{lum} (e.g. Front. Chem., 2020, 8, 527), such a small temperature deviation (21 ± 0.3 ˚C) only cannot account for the drastic variation of glum values shown in Figure 2g and 2h. Therefore, it is proved that the current manuscript showed consistent conclusion also from the aspect of temperature dependence of g_{lum}. The proposed approach is original and extremely significant in this research field. The results shown in the current manuscript are high quality and the paper is also well organized. I consider that the manuscript is now ready to be published in Nature.

Referee #2

(Remarks to the Author)
Dear Authors, dear Editor,
I have commented in detail this paper in my previous report. Let me just add something on this revision.

I am glad to see that this article has undergone a revision, responding to all more relevant concerns that were raised. From my side, I believe that it can now be published essentially as it is (with some attention to my latter comments).
Concerning my own ones, there a point in the rebuttal, which seems to deserve further clarification. I agree that "multiple decay pathways for a single starting state does not in itself lead to multi-exponential behaviour" and very clearly: "multiple starting states" scenario is not the relevant one here. The fact is that here we can't appreciate it, due to limited resolution of the emission monochromator or to intrinsically unresolved lines, but the D0->F1 is *not* a single line, but rather a triplet of lines, potentially each with its own decay rate, hence the (potential) multiexponential decay of the convoluted line.
I should also emphasize that the 90° excitation with H-polarized light in viscous media leads to much weaker emission spectra than with in-line excitation with unpolarized light. This said, I appreciate that the proposed setup uses an intrinsically LP-source and depolarizing it for in-line excitation would be a major complication and prone to error.

I would also like to add a small comment on the exchange with Referee 3 (comment 1), keeping in line with what I wrote just above.

When validating a new instrument or a new setup, comparing data collected with other instrumentation is essential, although it may represent some critical aspects. In the case of chiroptical spectroscopies and in particular of certain metal-centred CPL spectra, there is the very major problem of resolution. As well known in the literature, but clearly and recently pointed out in 10.1021/acs.inorgchem.4c00417, spectra appearance and even quantitative data strongly depend on acquisition parameters and instrumental features. Any comparison must not overlook this aspect and one may even accept that the match is only fair, provided there is a good explanation.
I should also point out that there is no “sign inversion within individual transition” (this can occur only e.g. in the presence of CP-reabsorption). Here one would deal with multiple nearby transitions due to MJ-splittings.
I notice that the g-factor plots are practically zero when the emission (and CPL) vanish. In fact, it should be an undetermined form (0/0). The authors should explain how they took care of this.
Finally, the authors recall potential contributions arising from linear dichroism/linear birefringence. This would apply in absorption, whereas here LD should be substituted by fluorescence anisotropy.

Open Access This Peer Review File is licensed under a Creative Commons Attribution 4.0 International License, which permits use, sharing, adaptation, distribution and reproduction in any medium or format, as long as you give appropriate credit to the original author(s) and the source, provide a link to the Creative Commons license, and indicate if changes were made.
In cases where reviewers are anonymous, credit should be given to 'Anonymous Referee' and the source.
The images or other third party material in this Peer Review File are included in the article’s Creative Commons license, unless indicated otherwise in a credit line to the material. If material is not included in the article’s Creative Commons license and your intended use is not permitted by statutory regulation or exceeds the permitted use, you will need to obtain permission directly from the copyright holder.
To view a copy of this license, visit https://creativecommons.org/licenses/by/4.0/

Referee #1:

This manuscript describes about “novel approach to achieve high-sensitive, broadband, time-resolved full-Stokes spectral measurements”. The proposed approach is original and extremely significant in this research field. The results shown in the current manuscript are high quality and the paper is also well organized. I recommend the publication of this paper in Nature after a minor revision as follows

We thank the reviewer for their appraisal of our work and have now addressed their minor revision comments as shown below.

1) It is known that glum value is significantly affected by sample temperature (e.g. Front. Chem., 2020, 8, 527). However, no information about the measurement temperature was shown in this manuscript. The authors should show all measurement temperatures.

We thank the reviewer for their helpful remark and fully agree that this is useful information for the reader to add. All measurements were carried out in ambient conditions in a laser lab with controlled and monitored temperature. The recorded temperatures on measurement dates (separated temporally by some months) are listed below. As all temperatures are 21 ± 0.3 ºC, we have noted this as the measurement temperature and added this to the Methods section.

$Eu(facam)_3$ µs TRCPL (Fig. 2): 21.2 ºC

$Eu(facam)_3$ steady state CPL (Fig. 2): 20.8 ºC

$Eu(facam)_3$ 5 ns TRCPL (Fig. 3): 21.3 ºC

$Eu(facam)_3$ 2 ns S_1 TRPL (Fig. 3): 21.0 ºC

BPC TRCPL (Fig. 4): 21.1 ºC

Rhodamine B TR-Stokes (Fig. 5): 20.8 ºC

Camphorquinone (Fig. S15-S17): 20.8 ºC

Added measurement conditions to the now created Methods Section:

Sample preparation and measurement conditions
[…]

Solutions were measured at ambient temperature in a laboratory with controlled and monitored temperature, recorded as 21 ± 0.3 ºC on measurement dates for all measurements.

2) In the insets of the Figures 2a and 2d, the way to show the Y-axis is not same ("x 1000" in Figure 2a and "x 10^4" in Figure 2d). The authors should standardize the format.

We thank the reviewer for pointing this out and have amended Figure 2 accordingly (below), with insets in subplots **a** and **d** now showing the y-scale orders of magnitude as "$\times10^{-3}$" and "$\times10^{4}$", respectively.

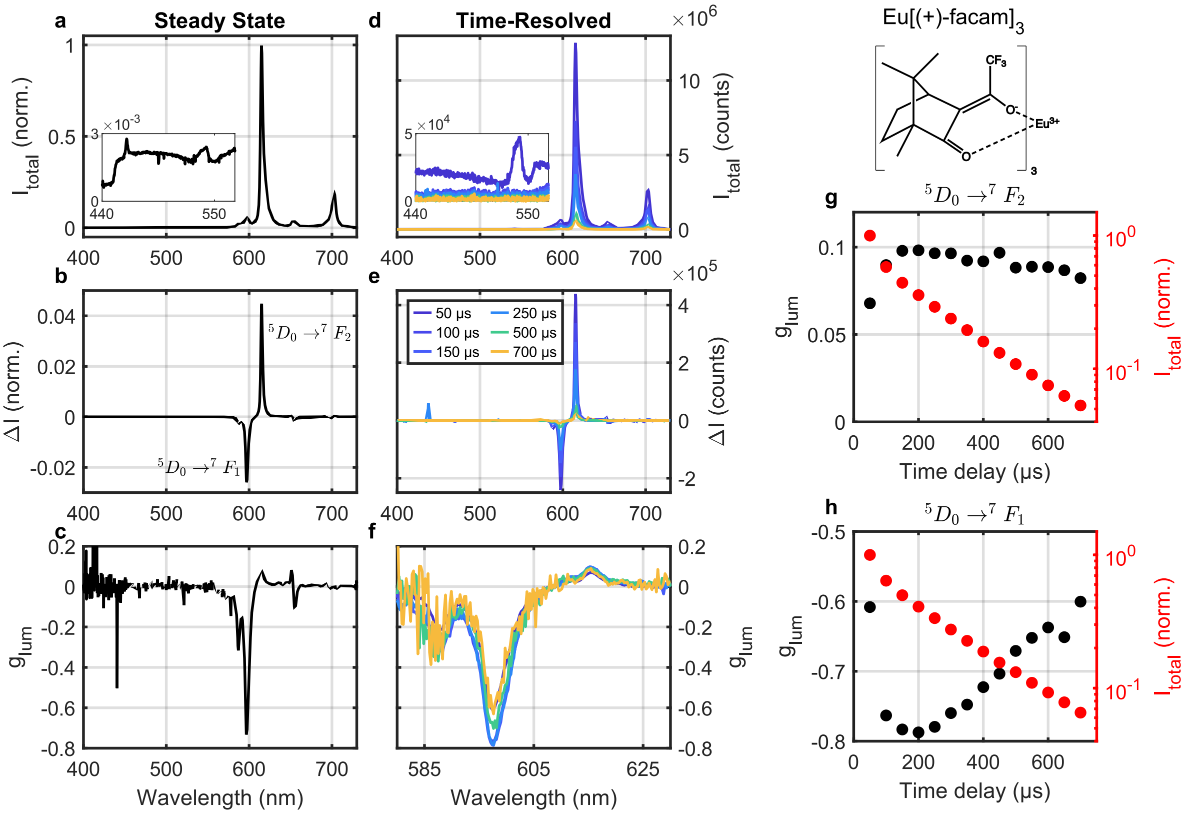

3) Please change "Hananel" to "Hananel et al." (line 26 in page 5, line 14 in page 7).
4) Please change "artefacts" to "artifacts" (line 24 in page 11).

We thank the reviewer for the close read in spotting these points and have amended the text accordingly. We have highlighted the changes in the main body.

We thank the reviewer again for their useful remarks in further improving the quality of our manuscript.

Referee #2:

This manuscript describes a very valuable contribution to the field of time-resolved circularly polarized luminescence (TRCPL), which is a topic I very warmly welcome and that I believe will have great prospects. I would welcome its publication, possibly following the suggestions that I provide below, labelled in curly brackets. Please notice that especially point {c} and {d} are only suggestions! Overall, the manuscript is clear, with a well introduced context, and it may call the attention of a wide readership.

We are very grateful for the appreciation of our work by the reviewer and that they welcome its publication. We are also grateful for their very helpful suggestions to further improve our manuscript and have implemented those suggestions as shown below.

The paper describes in detail the experimental setup that was used to achieve TRCPL, in such a way that it can inspire other groups in building analogue instrumentation. One or a few pictures of the actual setup may further be welcome in the SI, accompanying Fig. S1.

We thank the reviewer for their remark and have added additional pictures to the SI, as shown below.

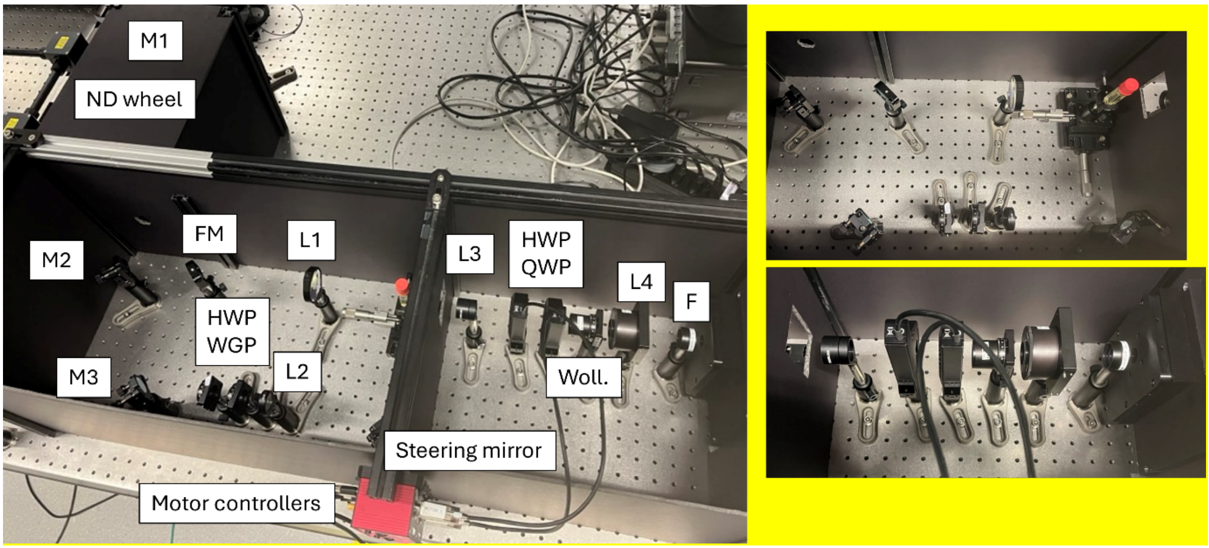

Figure S2: Pictures of the setup, with parts laid out and labelled as in Figure S1. WGP refers to a wire grid polarizer.

{a}

Noteworthy the proposed instrument allows for full Stokes polarization elements TR-measurements and for this reason it may extend beyond the realm of chirality and allow for fluorescence anisotropy depolarization measurements at the same time.
As examples of applications, the paper discusses a commercial standard, Eu(facam)3, whose TR-CPL had been reported in reference [22], and TADF molecule. In addition, rhodamine in different viscosity solutions is used to challenge the instrumentation for photoselection artefacts.

Polarization measurements and especially CPL may suffer from several artefacts, which are well known in the literature, but are nicely recalled and discussed in the SI. One aspect, which is not treated here, but which may be useful to mention are the artefacts arising from differential extinction (when ECD and CPL bands partially overlap). It should be straightforward to briefly discuss this aspect, as well, on account of its occurrence in most cases where organic fluorophores are involved and too often neglected.

We thank the reviewer for their remark and fully agree that this is a common artifact which we now discuss in an added Section 2.6 in the SI (see below). We also include an illustration of how such an effect may impact data and a simple approach to check for its presence in the measurement geometry we used. We also direct the interested reader to further relevant resources.

2.6: Circular Dichroism-induced CPL Artifacts

A well-known but easily overlooked source of erroneous CPL dissymmetry values is self-absorption in the presence of electronic circular dichroism (ECD). Corrections for such effects specifically for measurement geometries similar to ours can be derived (10.1002/chir.22647). Similar effects are seen in solid films, which may display substantial effects from self-absorption with ECD (10.1021/acs.chemrev.0c00195).

Here, we will briefly illustrate the effect when luminescence and the lowest-energy absorption feature have the same sign of dissymmetry (as expected, when the same transition is involved in both features).

For example: absorption and emission of right-handed light is preferred. In this case, right-handed light is preferentially emitted, but preferential self-absorption of the right-handed component in luminescence will act to reduce the magnitude of apparent CPL measured. If sufficient self-absorption occurs, a bisignate CPL spectrum could result.

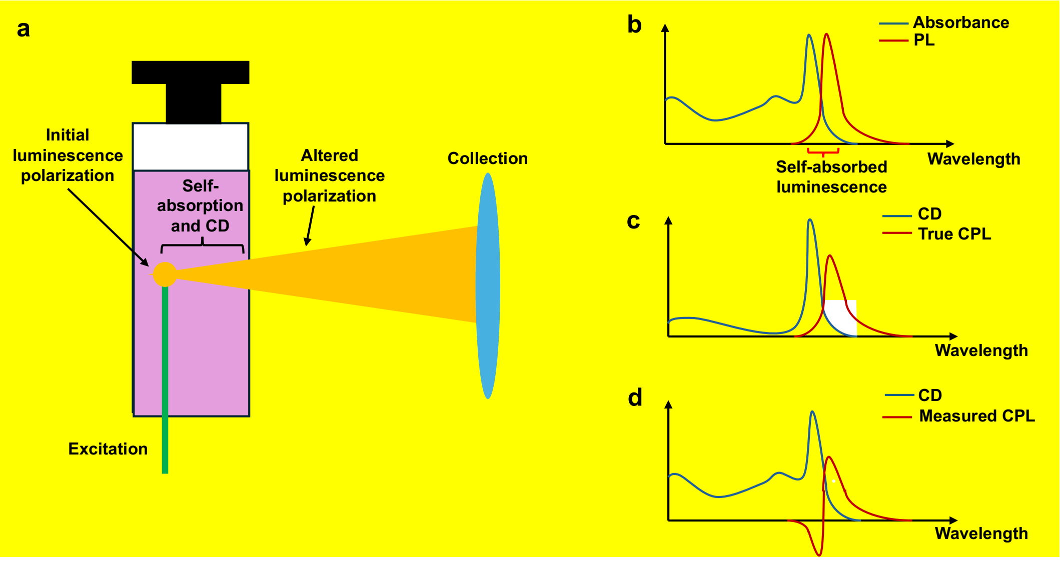

For a solution sample in a thick cuvette measured in the 90º geometry, one could in principle adjust the degree of self-absorption by controlling the position of the sample relative to the excitation beam, as illustrated below. While this may not fully remove the artifact directly, it can at least act as a "sanity check" to easily determine whether self-absorption is influencing the measured CPL values significantly.

Alternatively, a concentration series for a solution sample can also provide information about self-absorption effects, if measured dissymmetries are found to be concentration-dependent.

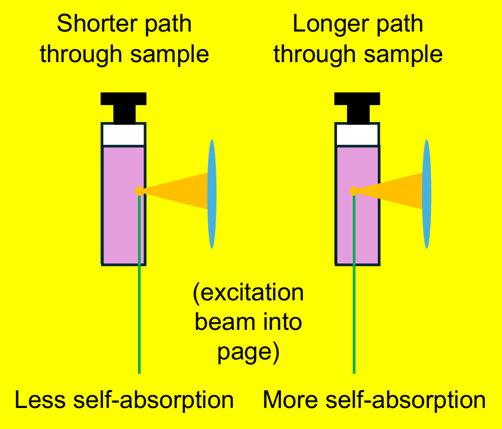

{b}
I believe that the detailed analysis of the transient processes occurring in Eu(facam)3 is of utmost interest and that it nicely complements Markovich et al.'s work of ref. [22]. To be even more thorough, it would be instructive to reproduce their data with wet DMSO, as well.

We fully agree that reproducing the wet DMSO data as initially reported by Markovich *et al.* (which we appreciate a lot) is insightful and have now measured this.

We have added Figure S19 to the SI showing the data with some context given in the figure caption. We have also updated the Methods section to describe this measurement (since the initial work, we have obtained an optical parametric amplifier allowing for a greater selection of excitation wavelengths).

We followed the methodology described by Markovich *et al.* as follows: The initial solution was freshly prepared in a glovebox using $Eu(facam)_3$ and anhydrous DMSO. We added 0.5% v/v water to the solution, waited several (approximately 5) hours, and measured. To ensure robust comparison, we also measured a sample in anhydrous DMSO with no added water during the same measurement session, using otherwise identical methodology (excitation wavelength, repetition rate etc.). The results are presented below.

Intriguingly, in our experiment we find the water-contaminated and anhydrous samples to act in a very similar manner. The trend of g_{lum} decreasing over time may be slightly weaker for the water-contaminated sample, and the overall luminescence decay appears slightly faster. However, the impact of water contamination is much less apparent here that what was observed by Markovich *et al.* in their work.

We note that the time-varying data showing decreasing anisotropy at longer time delays is similar to what was reported for anhydrous DMSO by Markovich *et al.* (by contrast, water-contaminated solutions in their work showed higher g_{lum} which remained approximately constant over time). Therefore, water contamination of the anhydrous samples in our work would not explain the discrepancy between our work and Markovich *et al.*'s study. Further, our new results in anhydrous DMSO are consistent with earlier experiments performed by us, such as shown in Figure 2 of the main body.

In these responses, we show the data sets collected in "wet" and anhydrous DMSO. A shorter summary figure for clearer comparison was placed into the SI, presented after these.

First, results in “wet” (0.5% v/v) DMSO:

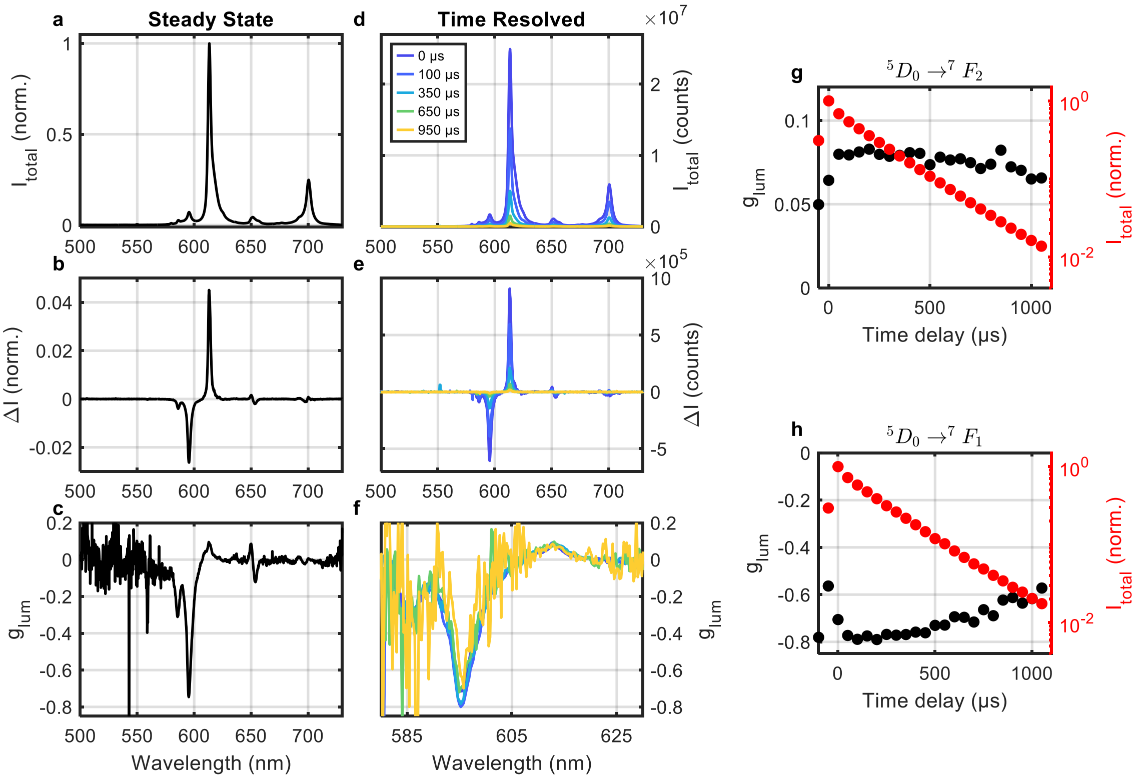

Results in anhydrous DMSO during the same measurement session, using otherwise identical methodology (excitation wavelength, repetition rate etc.):

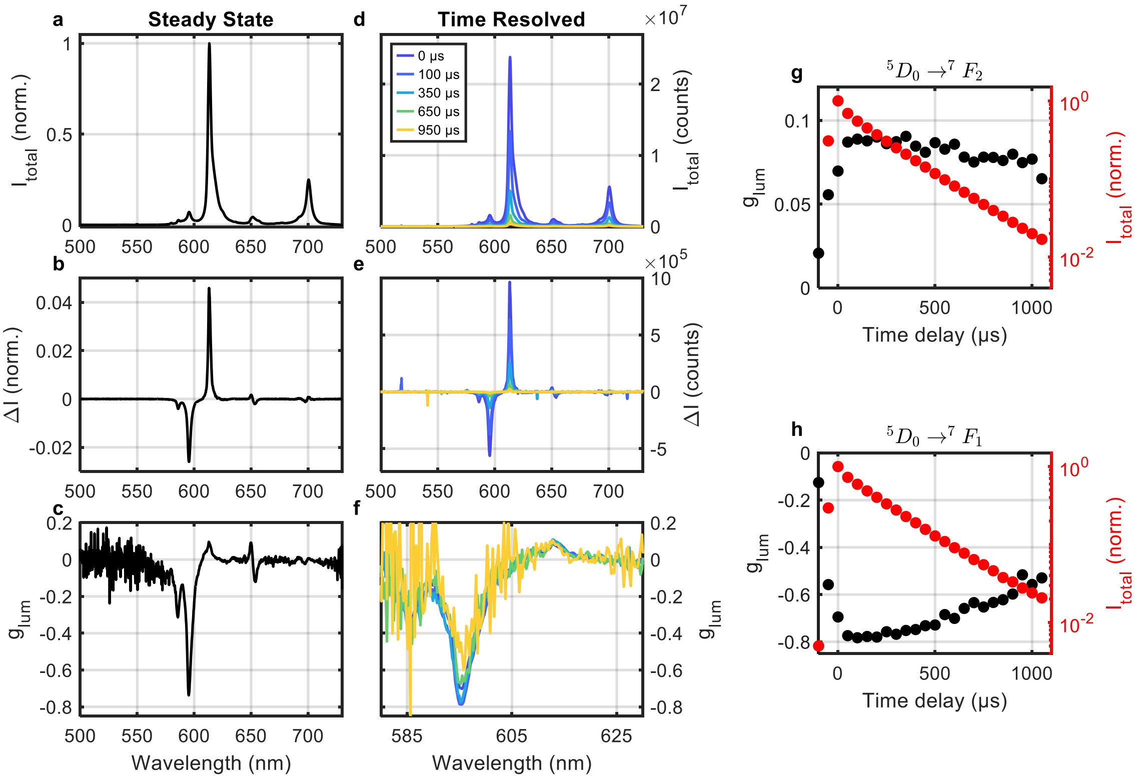

Added SI Figure S19 showing measurements for a "wet" DMSO solution sample of Eu(facam)$_3$ in steady-state and time-resolved CPL, as well as comparison between "wet" and anhydrous DMSO solutions measured during the same session with the same parameters:

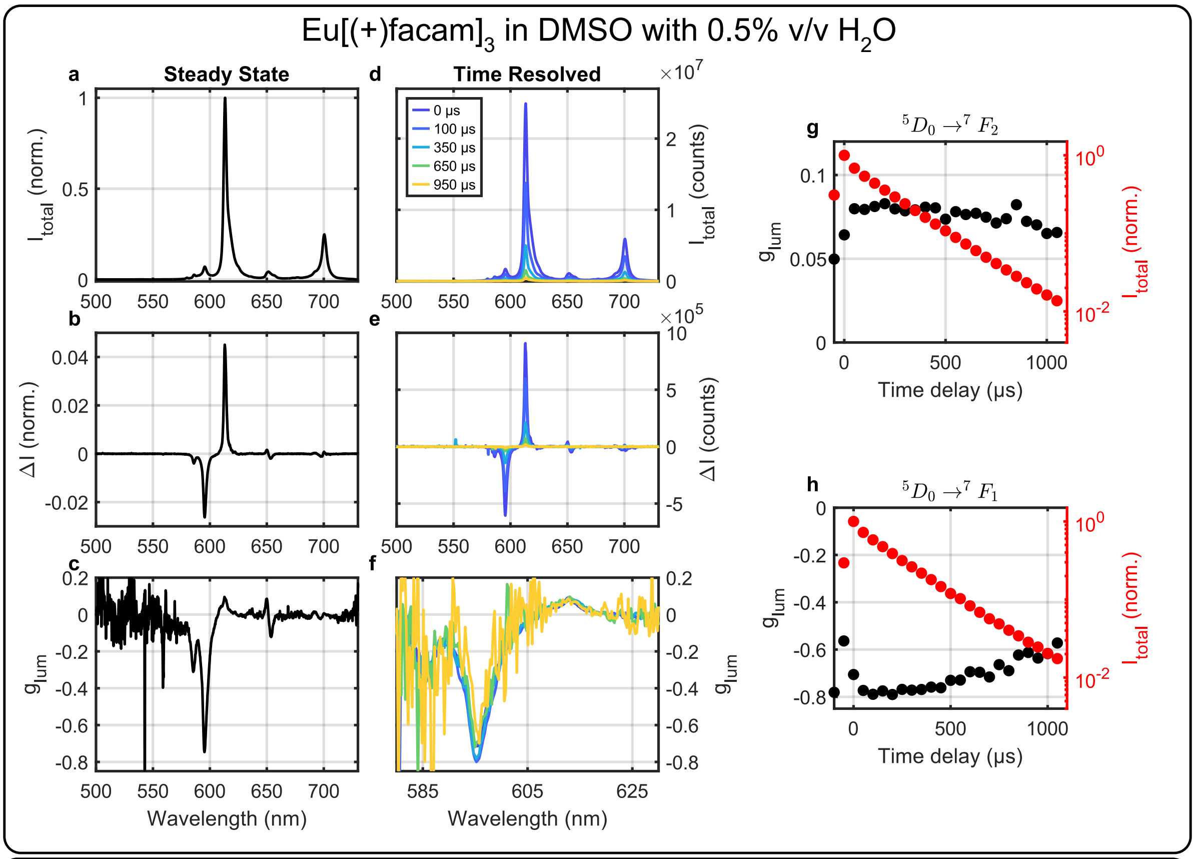

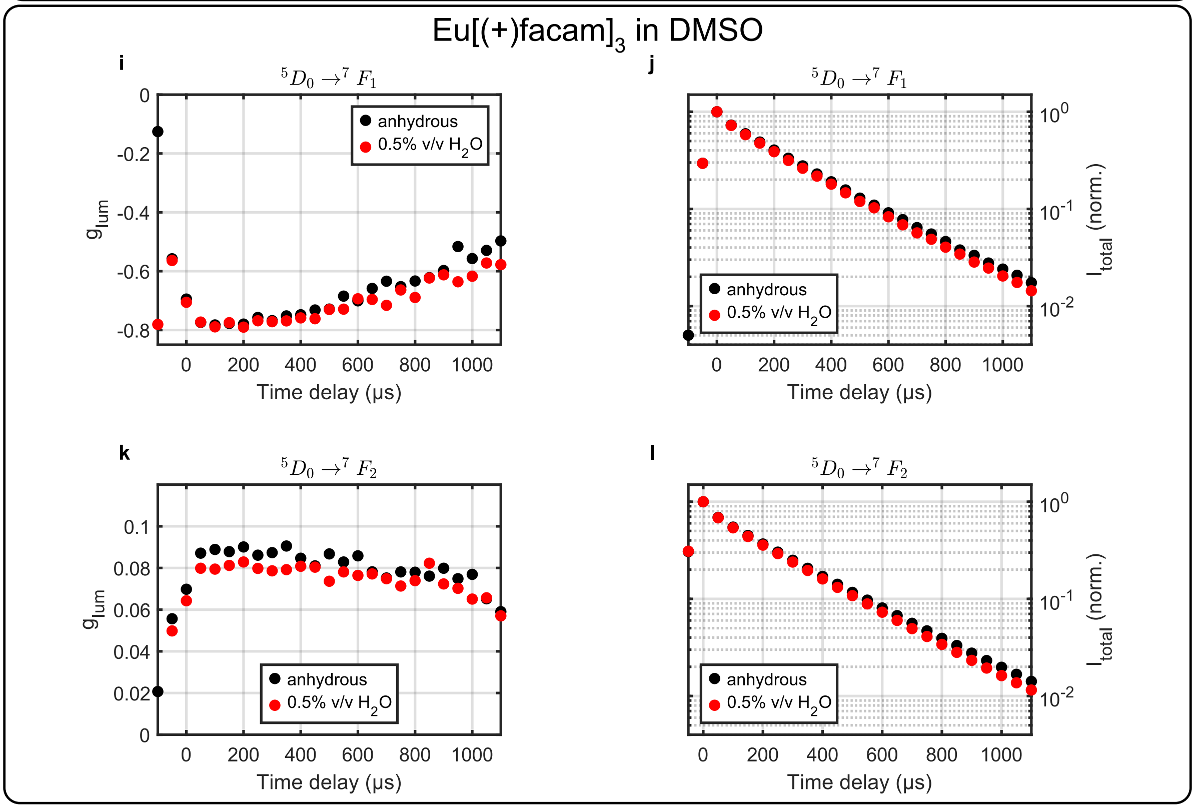

Figure S19: Time-resolved CPL measurements of Eu[(+)-facam]$_3$ in "wet" DMSO (with 0.5% v/v H_2O added, as done by Hananel et al. (16)). Excitation 373 nm, 200 fs, 500 Hz, horizontally polarized. ***a-c)*** *Time-averaged CPL measurement (2 ms time gate width).* ***d-f)*** *Time-resolved CPL measurement (50 µs time gate width and 50 µs gate steps) showing spectra at selected time slices.* ***g-h)*** *Intensity and dissymmetry factor as a function of time at the $^5D_0 \rightarrow {}^7F_2$ (near 615 nm) and $^5D_0 \rightarrow {}^7F_1$ (near 595 nm) luminescence peaks.* ***i-l)*** *Comparison of dissymmetry and total luminescence dynamics for Eu[(+)-facam]$_3$ in "wet" DMSO and in anhydrous DMSO (measured with the same parameters and concentration). Compared to results obtained by Hananel et al., the time-evolution of dissymmetry in anhydrous DMSO is similar but the impact of water contamination is less apparent.*

Added description of "wet" DMSO Eu(facam)$_3$ sample preparation and OPA sample excitation to the Methods section:

Sample preparation and measurement conditions

[…] The Eu[(+)-facam]$_3$ solution in "wet" DMSO was prepared by adding 0.5% v/v deionized water to the 11 mM Eu[(+)-facam]$_3$ solution, where the solution was measured 5 hours after the addition of water, similar to the methodology described in the work of Hananel *et al.*[16].

Sample excitation

[…] Other excitation wavelengths were generated using an optical parametric amplifier (Light Conversion ORPHEUS-NEO).

{c?}
Concerning the decay of Eu(facam)3 CPL, there is one aspect that may be worth discussing here or in the future. The D0-F1 band around 595 nm, is not a single transition, as well known and seen even here. This is due to MJ splitting and I am not sure that these components must decay at the same rate. This would account for non-constant glum and even for biexponential behaviour. I should very much appreciate the authors' comment, if they can (I understand that this may fall out of their expertise).

We thank the reviewer for the very interesting remark. This does indeed fall a bit outside our expertise. However, we can provide some discussion, yet, please note that the applicability to the specific circumstances may be limited.

Presumably the MJ-splitting here refers to the F1 states since the D0 states have J = 0. In general terms, the presence of multiple decay pathways for a single starting state does not in itself lead to multi-exponential behaviour. Rather, in such a case the result is a monoexponential decay at a rate which is the sum of the individual rates (assuming no back-transfer and lifetimes independent of excited-state concentration). In the case of multiple luminescent decay pathways, the resultant luminescence spectrum would be that of the individual transitions weighted by their relative decay rates, but all spectral features would decay at the same rate (as their intensity is proportional to the population of the excited state).

Similar considerations apply, if instead of a single starting state there are multiple sub-states in dynamic equilibrium decaying to a ground state (*i.e.,* the population transfer between the sub-states, both ways, is much faster than the transfer to the ground state; this would be expected given the small energetic splittings). In this event, the luminescence lifetime would be the weighted sum of the individual sub-state lifetimes (with the weighting being their Boltzmann population, governed only by their energetic separation and multiplicity in the limit of dynamic equilibrium). However, presumably this "multiple starting states" scenario is not the relevant one here.

{d?}
Concerning photoselection, besides horizontally polarized excitation, there is 180° geometry (in-line), as shown in Figure 1. This is a powerful means to cancel this very nasty artefact, but it seems to me that is not discussed here. From my own experience, I know that it may be extremely difficult to cope with the stray light of the UV-source, but I should be curious to hear the authors' experience. I should also like them to recall that in the limit of infinite viscosity (no molecular tumbling), horizontal excitation should lead to *no signal*. This often reduces the practical utility of this expedient.

We thank the reviewer for their comment. We agree that the 180º excitation-collection geometry does, in general terms, provide a viable pathway for mitigating photoselection effects. 180º geometry with unpolarized excitation and 90º geometry with horizontally polarized excitation are indeed the two configurations noted by Blok and Dekkers to result in negligible polarization artifacts in CPL measurements (10.1366/0003702904085679). For these reasons, we included in Figure 1 an illustration of both of these excitation-collection geometries available for use in our setup.

For our reported measurement cases, the 90º excitation-collection geometry was particularly suitable. A particular advantage is related to the fact that our excitation source is most often a pulsed ultrafast laser, polarized at the source. For the 90º geometry, minimizing artifacts is achieved by using horizontal polarization, easily achieved with high extinction ratio by a simple stock polarizer. On the other hand, the 180º would require unpolarized excitation to avoid artifacts. While depolarization approaches exist, this is experimentally a bit more complicated, especially to a high degree of depolarization with a pulsed laser.

Helpfully, all our samples were solutions in square-based four-window cuvettes. Such samples can easily be mounted at right angles to excitation and collection, avoiding polarization-sensitive reflection angles. We found this to be a robust and straightforward methodology.

However, we recognize that the 90º geometry faces limitations for other sample types such as solid-state samples, which are often thin films. Orienting such samples so that they are normal and homogeneous along the excitation-collection axes may not be possible. Issues of polarization-sensitive reflections from a sample mounted at an angle may become apparent. The point raised about effects in the limit of infinite viscosity are also especially relevant for such samples. In these cases, the 180º geometry is a valuable (and indeed possibly only) approach for artifact-free CPL measurements.

As a slight addition, we think that the recorded intensity in the 90º geometry in the limit of infinite viscosity is not zero in all cases. This will be affected by factors such as the symmetry of the molecule (electronically isotropic molecules cannot be photoselected) and possible energy transfer processes (10.1063/1.1668168).

As we agree fully that these points might be helpful for readers more generally, we have updated SI section 2.5 to include most of this discussion, specifically with added explanation regarding the 180º geometry, and added a reference to this section in the main body.

Rewrote SI section 2.5 to incorporate discussion of artifact-free CPL measurement configurations and practical considerations for choosing between these:

2.5: Photoselection Effects and How to Mitigate Them

Photoselection arises from preferential excitation of dipoles along the electric field (polarization) axis of excitation light. It is important to consider in CPL measurements due to their sensitivity to linear anisotropy-induced artifacts, with photoselection being a common source of linear anisotropy even in otherwise isotropic samples, as well described by Blok and Dekkers (*29*).

Photoselection can never be truly eliminated, as the propagating light beam contains no electric field component parallel to its propagation direction for any polarization (including unpolarized light). Dipoles along this axis can therefore not be excited. However, mitigation strategies for eliminating preferential linear polarization in detected light exist. Blok and Dekkers (*29*) point out two particular configurations where artifact-free CPL values can be recorded: orthogonal excitation and collection (90º geometry) with horizontally polarized excitation, and collinear excitation and collection (180º geometry) with unpolarized excitation. We briefly discuss these options, and the particular strengths of both configurations from a practical point of view.

Photoselection with a 90º Experimental Geometry

Preferential orientation of dipoles along an axis parallel to the light collection axis will result in uniformly (un)polarized light at the collector. This is illustrated in the cartoon below, and is the situation when the excitation polarization is horizontal and the collection axis is orthogonal to the excitation axis (*i.e.*, using the 90º geometry).

On the other hand, using vertical polarization in the same 90º geometry results in maximally polarized light along the collection axis. These two experiments therefore provide a convenient comparison for minimizing and intentionally maximizing photoselection effects in the collected light.

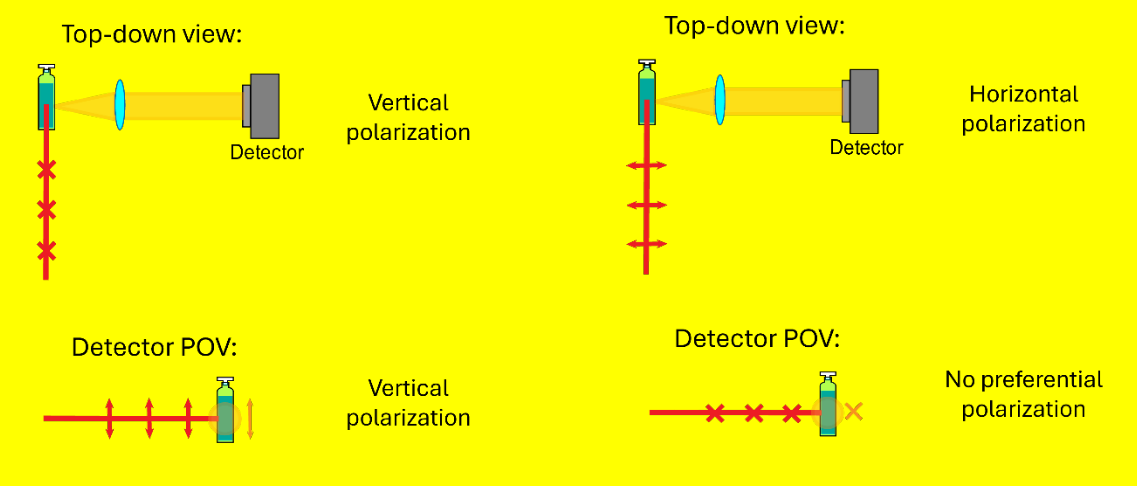

A particular advantage of the 90º geometry is that the avoidance of photoselection-induced preferential linear polarization in the collected light can be achieved by using a well-defined linear polarization state. This can be experimentally achieved straightforwardly and with high extinction ratio using a suitable polarizer. This is especially relevant for excitation sources such

as ultrafast pulsed lasers which are strongly polarized at the source and for which depolarization to a sufficient degree may not be experimentally trivial.

Further, solution samples in square-based cuvettes can be oriented at right angles to excitation and collection, avoiding polarization-selective reflection angles and yielding a robust and straightforward methodology.

Photoselection with a 180º Experimental Geometry

However, for samples such as thin films, mounting the samples to be normal to and homogeneous along the excitation-collection axes is often not possible. Polarization-selective reflections may become an issue when the excitation is incident at an offset angle. Further, emission intensity along an axis orthogonal to the excitation may be significantly reduced in the limit of infinite viscosity and limited depolarization (10.1063/1.1668168).

In such cases, the 180º provides a valuable alternative for mitigating photoselection effects in collected luminescence. In this case, even a thin sample can be mounted orthogonal to the excitation and collection axes.

In a 180º measurement geometry, both vertical and horizontal excitation polarizations will result in linear photoselection effects in the collected luminescence (illustrated below). Instead, unpolarized excitation light is thus required in this measurement geometry to minimize polarization artifacts in CPL measurements.

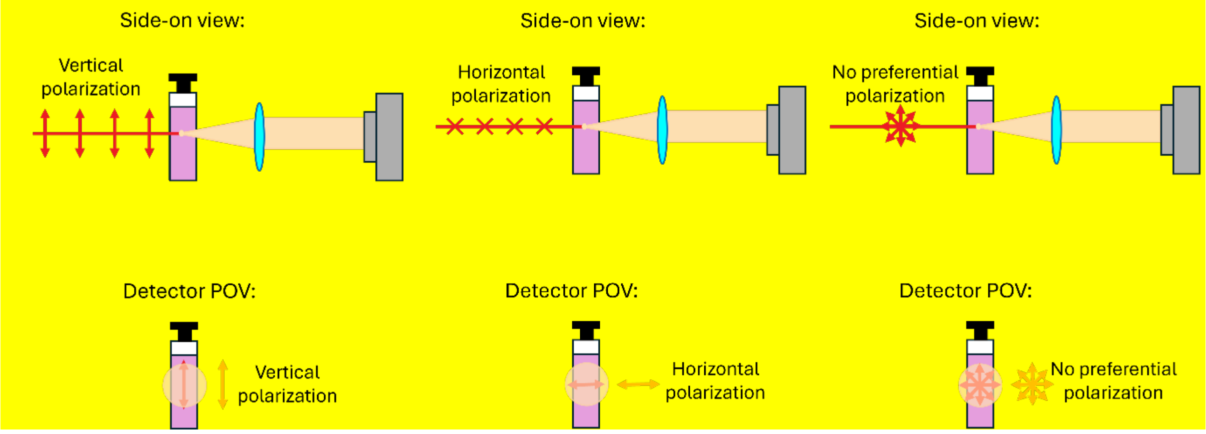

Added a reference to this updated SI section to the main body (immediately after Figure 1):

All data presented in this work was collected from solution samples, for which an orthogonal geometry with square-based four-window cuvettes is the most straightforward approach (SI Section 4.1). In this geometry, photoselection effects can be minimized by using a horizontal excitation polarization and maximized by a vertical excitation polarization.

We would like to thank the reviewer again very much for stimulating such a fruitful discussion on CPL detection through their thoughtful suggestions.

Referee #3:

The work demonstrates a new design for a CPL spectrometer using a single CCD spectrometer to collect both left and right circularly polarised light simultaneously by separating using rotating waveplates and a Wollaston prism. The work is not novel enough and of broad interest to be published in Nature. The work is a stepwise improvement from the controversial work published by Baguenard et al in Nature Communications in 2023 where they achieved separating left and right circularly polarised light with a polarising beam splitter and then recombined light from fibre optics into a single CCD. This work does not represent a significant enough improvement on that work to warrant publishing in Nature.

We thank the reviewer for sharing their opinion. While questions of novelty and significance are inherently subjective, we present arguments for why we believe our work represents significant progress in the field and is of interest to a broad scientific readership beyond our niche.

We point out that chiroptical spectroscopy in general is very widely utilized in fields ranging from biology to chemistry and physics, with well-known applications in medicine and, especially recently, photonics and materials engineering where CPL-emitting devices and materials are actively developed, for applications ranging from holography to quantum information technologies. We therefore feel that significant advances in methods with such widespread applications are of interest to a very general audience.

The work of Baguenard *et al.* is appreciated by us as relevant and valuable background to our manuscript, and we reference it as such. However, the work of Baguenard *et al.* does not contain any aspect or consideration of time resolution, instead demonstrating steady-state experiments for which well-established and highly sensitive methodology already exists.

We observe, explain and demonstrate that our developed methodology is especially well-suited for time-resolved CPL experiments, for which existing techniques are strongly limited in timescales, sensitivity, wavelength ranges and acquisition times achievable. As a result, TRCPL has previously only been feasible for a small subset of materials (generally lanthanide complexes) and even in these, broadband measurements or access to ns timescales (on which several energy transfer steps occur) have been previously unachievable. Time resolution, a key component of studying materials generally, has therefore been practically unattainable for CPL spectroscopy of many (if not most) materials systems of interest in the field.

Beyond time resolution, the sensitivity achieved by us is superior to what is demonstrated by Baguenard *et al.* by almost an order of magnitude, with us measuring dissymmetry factors on the order of 10^{-3} (as opposed to 10^{-2} measured by Baguenard). This is important because 10^{-3} is commonly the scale at which CPL-active organic molecules in solution exhibit dissymmetry, demonstrating applicability to a far wider range of materials than before.

Simply put, we do not believe that any previously published methodologies would be capable, in practice, of producing the data sets we have collected and presented here.

There are also more specific additional concerns with the work:

1. The work does not show a comparison to conventional scanning monochromator-PEM based CPL spectrometers. This is essential to show that spectral form in ΔI is maintained on the same sample. Comparing glum only is not enough to validate a CPL spectrometer, particularly when comparing lanthanide complexes as done in this work, as they can have unique sign inversions within individual transitions.

We thank the reviewer for their comment. We fully agree that comparison of the data collected with our technique to that from conventional PEM-based CPL spectrometers is useful. To this end, we have used such a (monochromatic, steady-state-only) setup to collect CPL spectra (both ΔI and g_{lum}) for the CPL-active materials studied in the main body of our manuscript.

We point out that results are consistent between setups both for the lanthanide complex and the organometallic TADF molecule. For the former, sign inversions specifically pointed out by the reviewer are consistent both for ΔI and g_{lum}.

We also note that, while comparing both ΔI and g_{lum} certainly gives more information than just one or the other, the quantity ΔI will in general incorporate the transmission and sensitivity characteristics of the measurement setup whereas g_{lum} is insensitive to these. Therefore, comparison of ΔI between a PEM/monochromator/PMT setup and a Wollaston/iCCD setup will not necessarily yield identical ΔI spectra. This, however, is inherently the case with any spectroscopic measurement, including comparison of different PEM-based instruments (*e.g.* from different vendors), and is not specific to our TRCPL technique.

Added SI section describing methodology for PEM-based CPL measurements:

1.2: PEM-based CPL setup

To establish consistency of our approach with standard photoelastic modulator (PEM)-based (monochromatic, steady-state) CPL detection systems, we have collected some steady-state CPL (SSCPL) spectra using such a PEM-based setup. Setup specifics and details of its operation are described at length in our earlier work (19). The setup consists of the following main components:

Excitation source: 365 nm LED (Thorlabs Solis 365C)

PEM: Hinds PEM200

Linear polarizer: Thorlabs WP25M-UB

Monochromator: Acton SP-2155

PMT: Hamamatsu H10723-20

Lock-in amplifier: Zürich Instruments MFLI 5 MHz

To block scattered excitation light, a 365 nm bandpass filter was placed between the LED and the sample and a 380 nm longpass filter was placed between the sample and detector.

Added PEM-based steady-state CPL measurements to SI section 3 (“Additional data”):

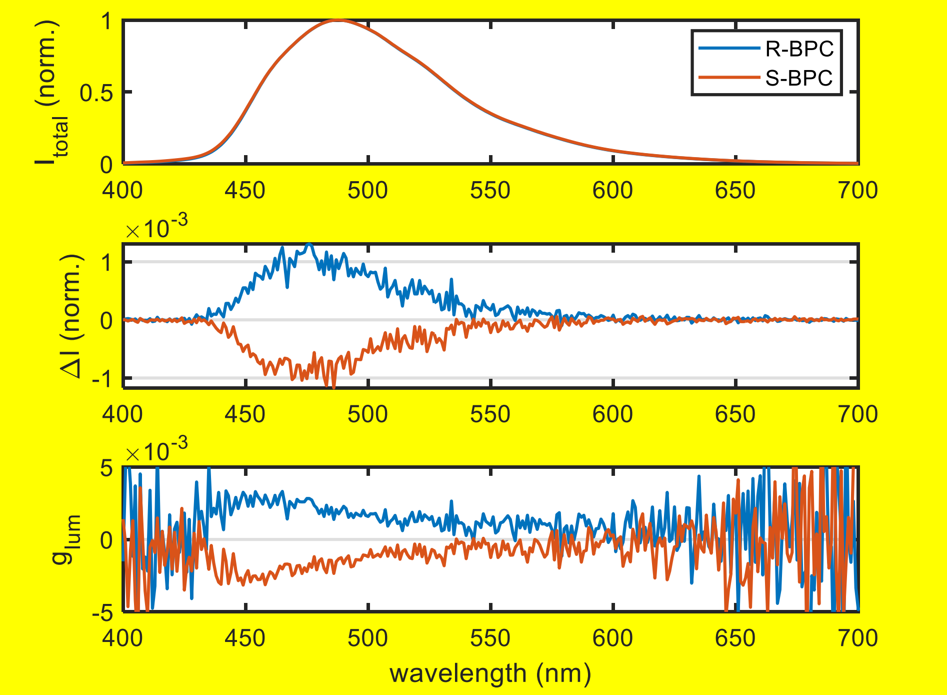

Figure S20: Steady-state CPL spectra of the chiral TADF-active compound BPC in toluene solution. Data for both enantiomers is shown. Spectra were acquired using a PEM-based CPL setup (excitation with 365 nm LED, continuous wave).

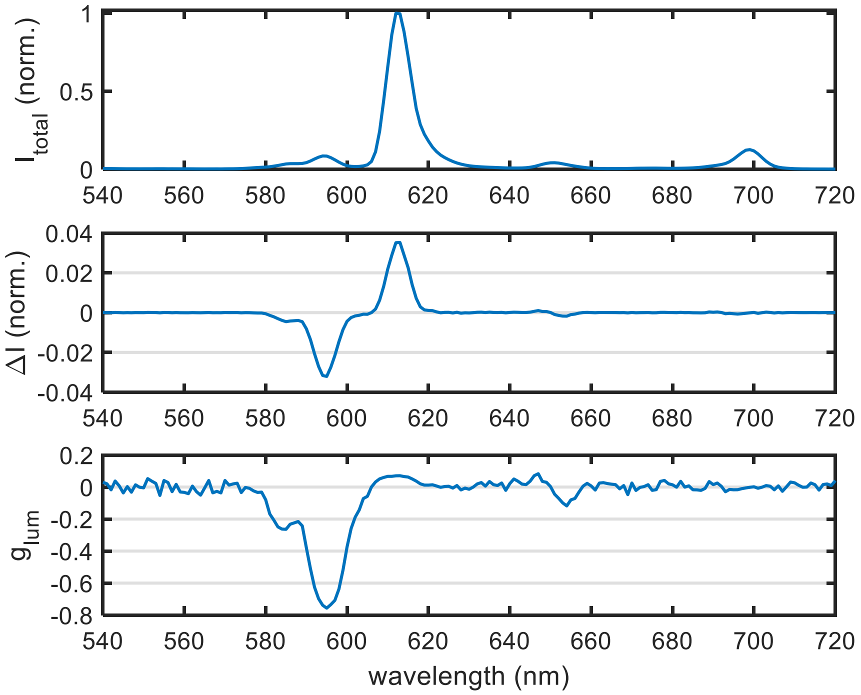

Figure S21: Steady-state CPL spectra of the chiral lanthanide complex Eu(facam)$_3$ in DMSO solution. Spectra were acquired using a PEM-based CPL setup (excitation with 365 nm LED, continuous wave) and previously included in our publication describing that setup (19).

Since the spectral range of Figure S21 is smaller than Figure 1 a-c (as the steady-state emission of Eu(facam)$_3$ has little discernible emission below approximately 550 nm), we have also attached to these responses a zoomed-in version of Figure 1 a-c with axis limits matching Figure S20 for the reviewer's convenience (see below).

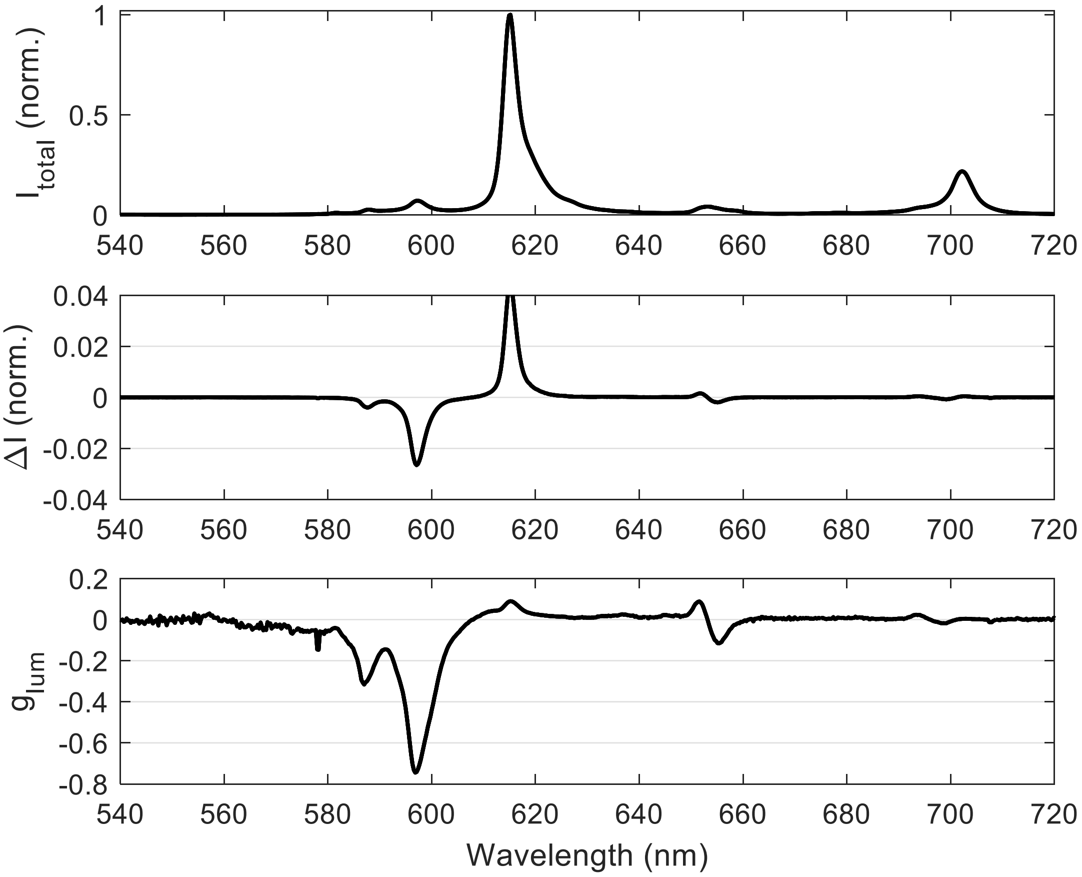

2. The use of a motorised rotating mount to rotate the wave plates within the work introduces significant error that does not seem to be accounted for. The manufacturer claims the mount has a repeatability of ±0.1° which at first glance seems negligible but on repeated rotations the mount can drift and introduce error.

We thank the reviewer for their comment. We agree that mechanical stability of components is a relevant consideration for this, or any, setup. We are always looking for ways to improve setup robustness and welcome suggestions and insights.

However, there does not appear to be any indication in our data apparent to us or presented by the reviewer to support the hypothesis that the motorized mount position repeatability introduces any "significant error" as stated.

We note that, as illustrated in Figure 1c of the main body, the left-and right-handed intensities are sinusoidally varying functions of the QWP angle with a period of 180º. The QWP angles used for data collection are extremal points, about which the gradient of the function is zero. Linear components are likewise sinusoidal functions of the HWP angle, collected at extremal points.

From the small-angle approximation about an extremal point, the expected relative amplitude variation for fully circularly polarized light from the QWP angle deviation of 0.1° (0.00174 rad) is on the order of $\Delta\theta^2 \approx 3 \times 10^{-6}$. We note that this is indistinguishable within the accuracy of our instrument by several orders of magnitude. Further, since the amplitude of the sinusoid is proportional to the degree of polarization, for a partially (un)polarized input state (which for real materials is what is almost always measured) this variation will be even smaller.

Therefore, in the absence of evidence suggesting otherwise, we feel confident in stating that motor accuracy is not, to the best of our understanding, a significant error source in the results presented here.

Finally, the specific choice of motor is not a key aspect of the reported technique. For the sake of easy adoption by others we have used off-the-shelf components from standard suppliers and included a full component list in the SI. Should motor accuracy present a long-term limiting factor in setup accuracy, replacement of the motor in question to one of higher specification would be a trivial change (*e.g.* https://www.zaber.com/products/rotary-stages).

3. The Wollaston prism used does not have even deflection across all wavelengths, is this accounted for?

We thank the reviewer for the comment. A similar caution regarding Wollaston prisms was raised by Baguenard *et al.* who instead used a polarizing beamsplitter. From data obtained from the manufacturer and collected by us, we found this effect to be small to the point of deflection variation being barely visible in our setup, with no apparent effects observed (or expected) on the CPL data collected.

As this point may be of interest to readers, we have now added a section to the SI to present our findings, alongside our motivation for using a Wollaston prism (which offers superior extinction ratios and broader response compared to standard polarizing beamsplitters, both of which are key performance specifications directly impacting quality of broadband CPL measurements). This will provide a more detailed answer to the reviewer's question.

Added SI section 4.8 with Figure S24:

4.8: Wollaston prism: Deflection angle wavelength dependence

Compared to standard polarizing beamsplitter cubes or plates, Wollaston prisms can offer superior performance in terms of extinction ratio and polarization splitting over a wide wavelength range. In our measurement scheme, both output beams exiting from the same face at symmetric deviation angles is also advantageous for a splitting/collection approach minimizing the total amount of optical components necessary and thus also potential artifact sources.

However, the deflection angle of Wollaston prisms is wavelength-dependent in general, and it has been suggested that this could necessitate complex workarounds in applications like ours (*31*). To address such concerns, we briefly present data showing that a suitable choice of components will result in the wavelength-dependence being miniscule.

For the Wollaston prism used (WPQ10, Thorlabs), the manufacturer provides data of the variation in deflection with wavelength. Over the full span of the approximate operation range of our instrument (ca. 400-900 nm) this deviation is less than 0.1º.

To demonstrate the effect on our setup specifically, we refer to Figure S24, which displays a full-sensor image of rhodamine B luminescence (horizontally zoomed to the emission region) showing the two polarization tracks. To help visualize possible line shifts in the vertical sensor direction, we have also plotted lines showing vertical positions of peak counts at each wavelength.

The measurement in question shows a variation in peak y-position of approximately 1-2 px over 150 nm, which is small compared to the sensor size (512 px), track separation (~150 px) and vertical peak width (~10 px).

During actual measurements, vertical binning is performed over two regions which are ~200 px in height (the top and bottom halves of the sensor, approximately). The full line is therefore collected regardless of small variations in vertical position.

In summary, the variation in angular deviation is small to the point of being barely detectable, and especially with vertical binning over a much larger pixel range is unlikely to result in

significant changes of the collected signal. In particular, Baguenard *et al.* found their CCD-based CPL measurements insensitive to much larger tilt misalignments (31).

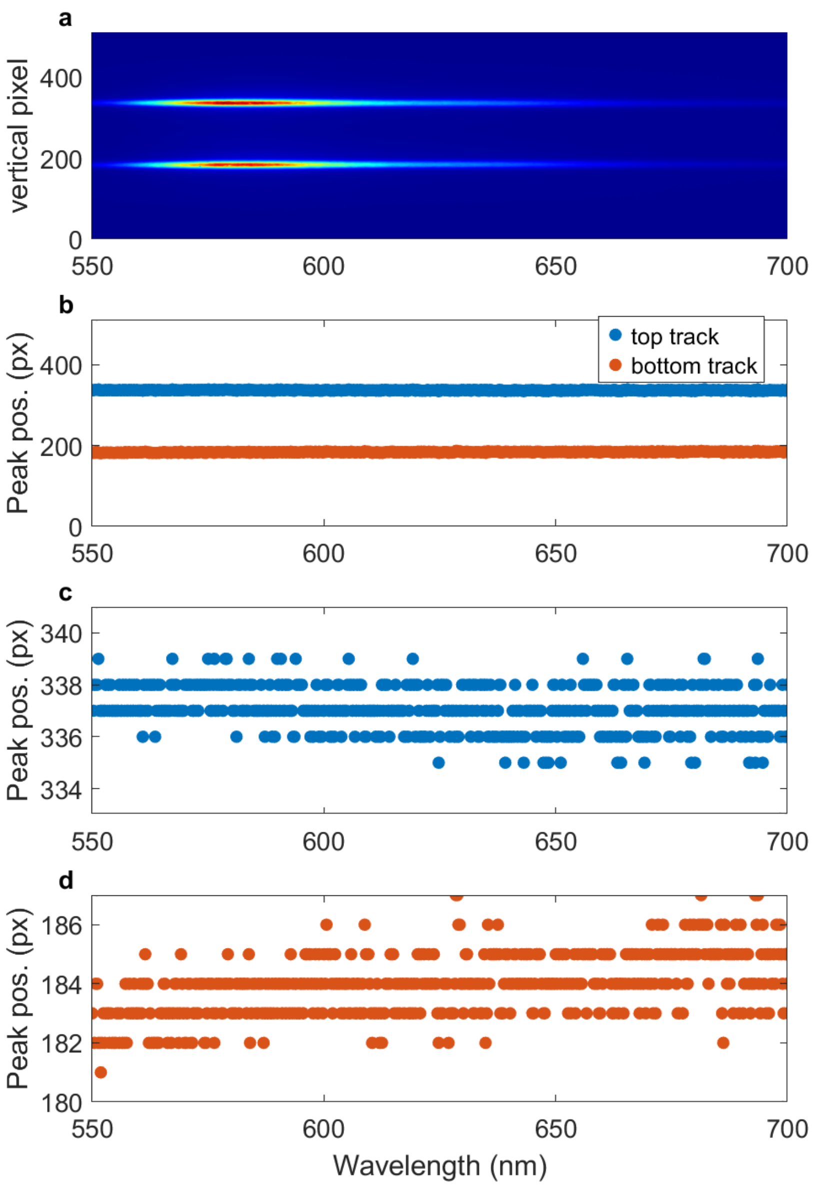

Figure S24: a) Full-sensor image showing orthogonally polarized tracks (rhodamine B in water, 343 nm excitation, vertical ex. polarization). b) Vertical pixel peak positions for the upper and lower tracks. c/d) Zoomed-in traces of vertical peak positions for the top/bottom tracks, respectively.

4. Figure S21 suggests that there is an inherent bias in the spectrometer that is quite significant for a non CPL active emitter.

We thank the reviewer for the comment. We will first point out why the figure in question (Figure S27 currently, Figure S21 in the earlier SI version) shows an offset, and then show that the instrument does not have a significant inherent bias in general.

It is important to note that Figure S27 is in a section of the SI where various error sources are discussed, and their effect illustrated with data. The inclusion of such "debugging" data in the SI was an effort to aid the adoption of our methodology by others, as many of these error sources are not immediately obvious, and it is likely that future users might run into similar issues initially.

Figure S27 was collected with a vertical excitation polarization, as stated in the figure, leading to a CPL artifact despite the emitter being achiral. The characterization of such artifacts is a substantial part of the efforts in measuring CPL accurately, and linear polarization artifacts are discussed in the main text. Data of the same sample, collected during the same measurement session using horizontally polarized excitation, shows negligible baseline offset. We have added this to Figure S27. In conclusion, the baseline offset seen in Figure S27 is mainly due to a polarization artifact caused by the vertical photoexcitation and subsequent photoselection.

We found that the offset artifact in Figure S27 retained its shape and most of its amplitude despite the large reduction in noise level upon beam resteering. We felt that this is a valuable point to include for clarification of exactly what is affected by beam steering (the noise level is, the artifact is largely not). For this reason, illustration by using data with an offset artifact is particularly useful for Figure S27.

Besides the renewed Figure S27, we have reproduced for the reviewer's convenience multiple figures from the manuscript showing measurements with low baseline offsets (generally below 10^{-3}). We believe this to conclusively demonstrate that sufficient experimental design allows us to eliminate inherent biases to a very high degree.

However, these aspects were perhaps not stated with sufficient clarity in the original SI section, and we felt that clarifying the surrounding SI section as well would likely benefit the wider readership.

We have updated Figure S27 to include the horizontally polarized measurement and updated its caption. In doing so, we found a slight inaccuracy in the caption of Figure S27, namely, the data was collected with a 343 nm excitation wavelength unlike the 515 nm excitation used for the Rhodamine B results presented in Figure 5 of the main body and Figures S10-S12 of the SI. We have taken this opportunity to amend the caption. For the sake of completeness, we included baselines for all Stokes components measured at 343 nm excitation in Figure S13.

For further clarification, we rewrote parts of the SI section in question (Section 4.10) to more explicitly address measurement artifacts and show examples of how they can be mitigated.

Modified Figure S27 (formerly Figure S21) and its caption:

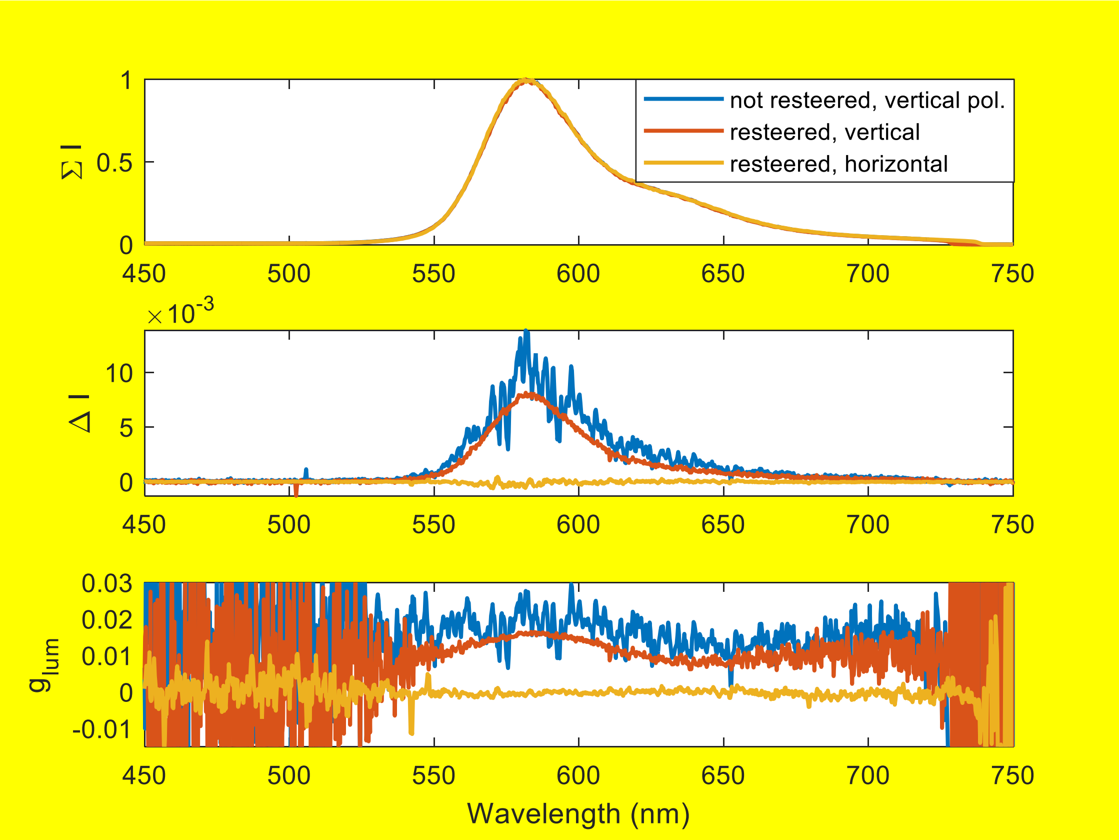

Figure S27: CPL measurement of rhodamine B in aqueous solution (excitation 343 nm, 200 fs, 50 kHz). Vertically polarized excitation leads to a photoselection-induced CPL artifact as discussed in the main body, resulting in apparent CPL for this achiral dye. Data showing such an artifact was measured with and without manual resteering of the beam between waveplate rotations, with otherwise exactly the same parameters. Resteering greatly reduces the apparent noise level, but preserves most of the shape and intensity of the CPL artifact. For a horizontal excitation polarization no CPL artifact is present, and a low-noise baseline is measured upon beam resteering.

To assure the reviewer that the setup does not have a significant baseline offset, we have also copied here figures from the SI demonstrating absence of significant baseline offsets in various measurements of achiral compounds (rhodamine B) and racemic mixtures (camphorquinone), when horizontal excitation polarization is used (see below).

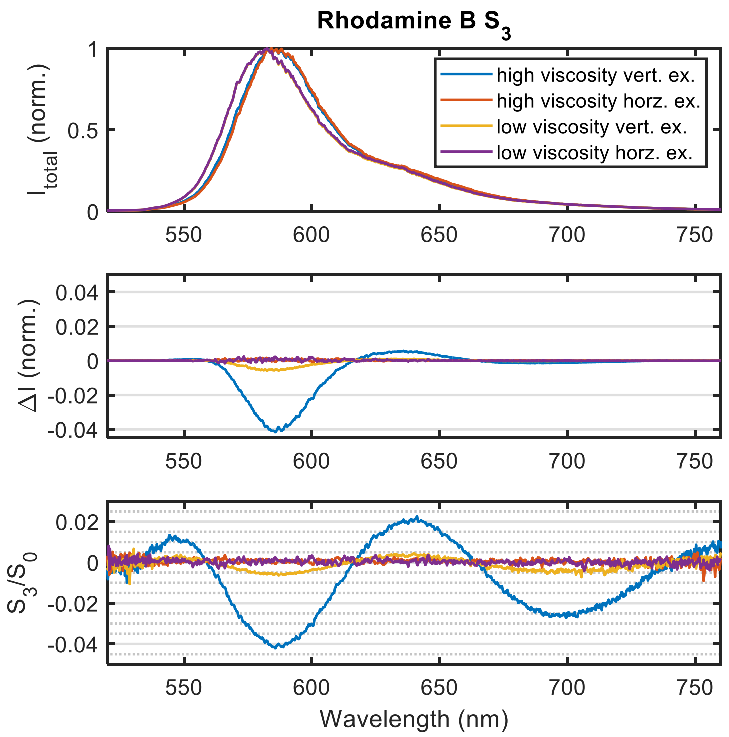

Figure S12: S_3 Stokes measurement of rhodamine B in aqueous solutions with horizontal and vertical excitation polarization (excitation 515 nm, 200 fs, 50 kHz).

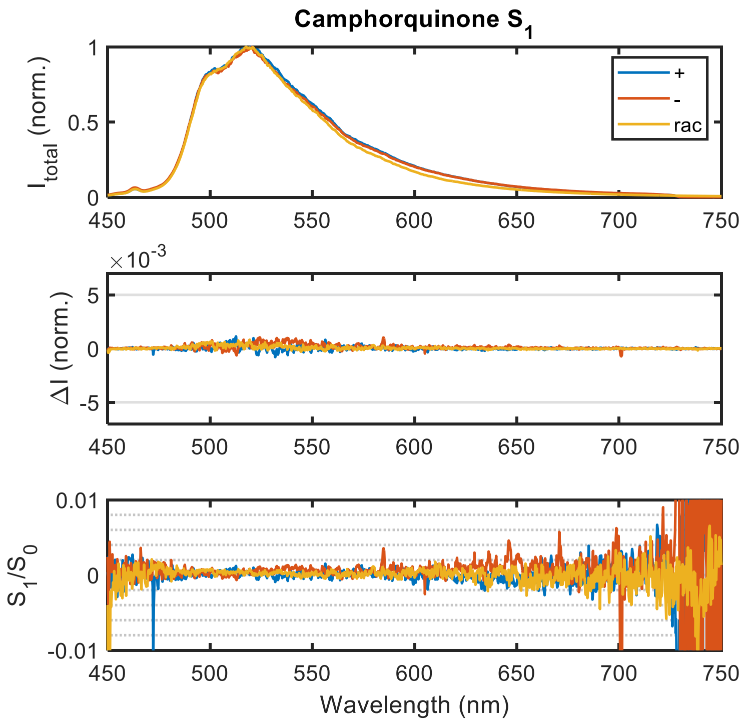

Figure S15: S_1 Stokes measurement of camphorquinone (both enantiomers and racemate) in aqueous solutions with horizontal excitation polarization (excitation 405 nm, CW).

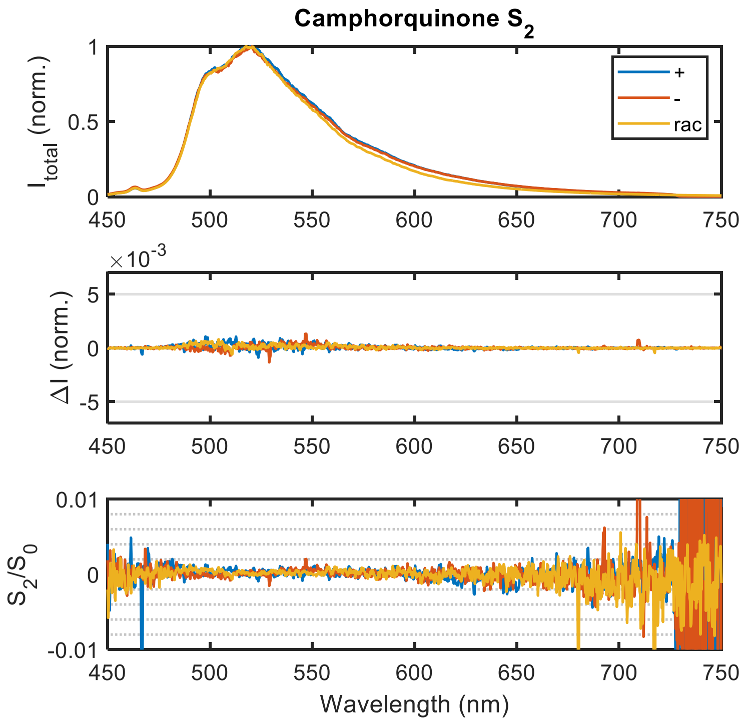

Figure S16: S_2 Stokes measurement of camphorquinone (both enantiomers and racemate) in aqueous solutions with horizontal excitation polarization (excitation 405 nm, CW).

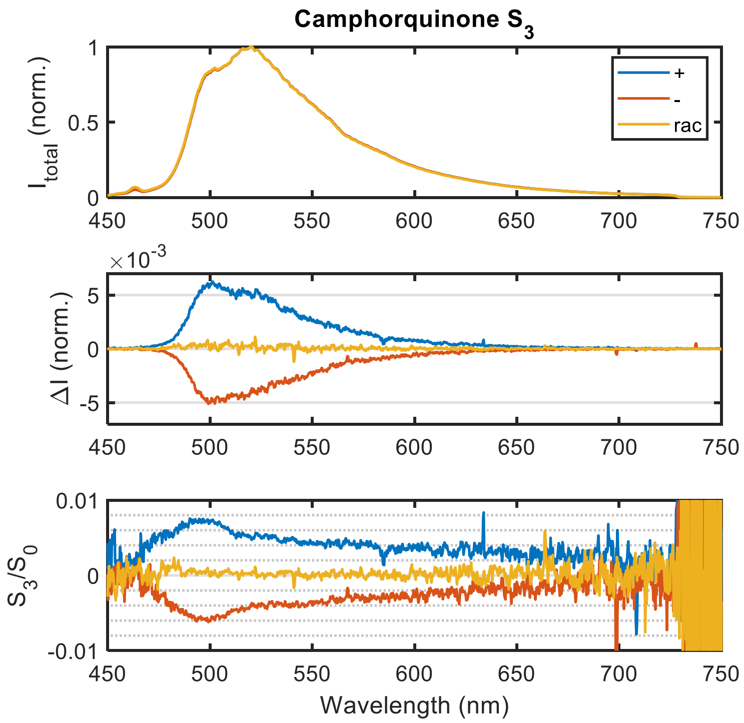

Figure S17: S_3 Stokes measurement of camphorquinone (both enantiomers and racemate) in aqueous solutions with horizontal excitation polarization (excitation 405 nm, CW).

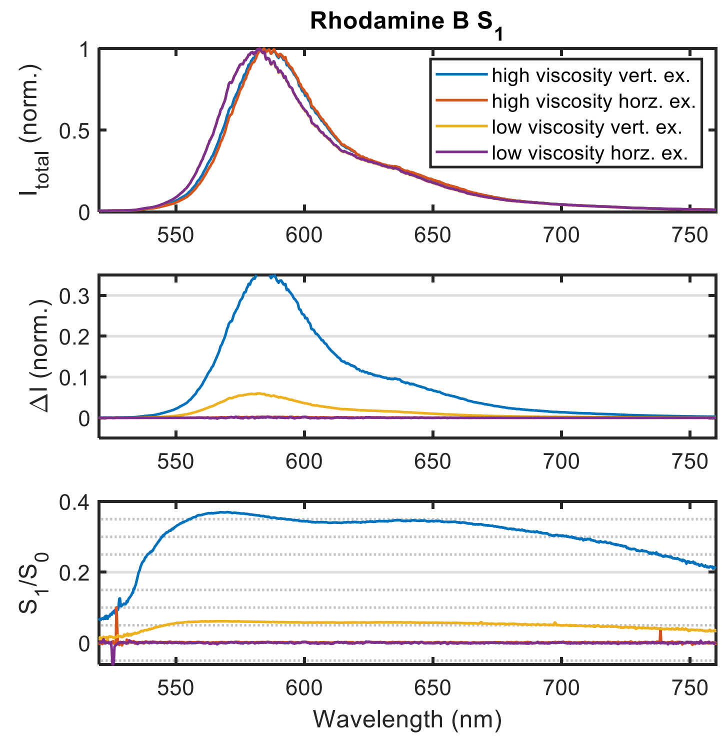

Figure S10: S_1 Stokes measurement of rhodamine B in aqueous solutions with horizontal and vertical excitation polarization (excitation 515 nm, 200 fs, 50 kHz).

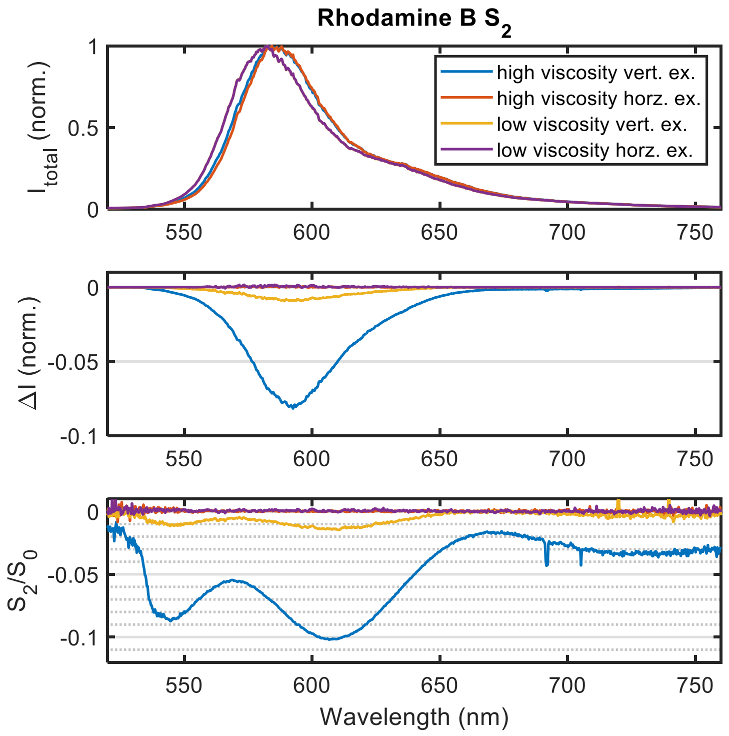

Figure S11: S_2 Stokes measurement of rhodamine B in aqueous solutions with horizontal and vertical excitation polarization (excitation 515 nm, 200 fs, 50 kHz).

Added Figure S13 to display baselines for all Stokes components at 343 nm excitation:

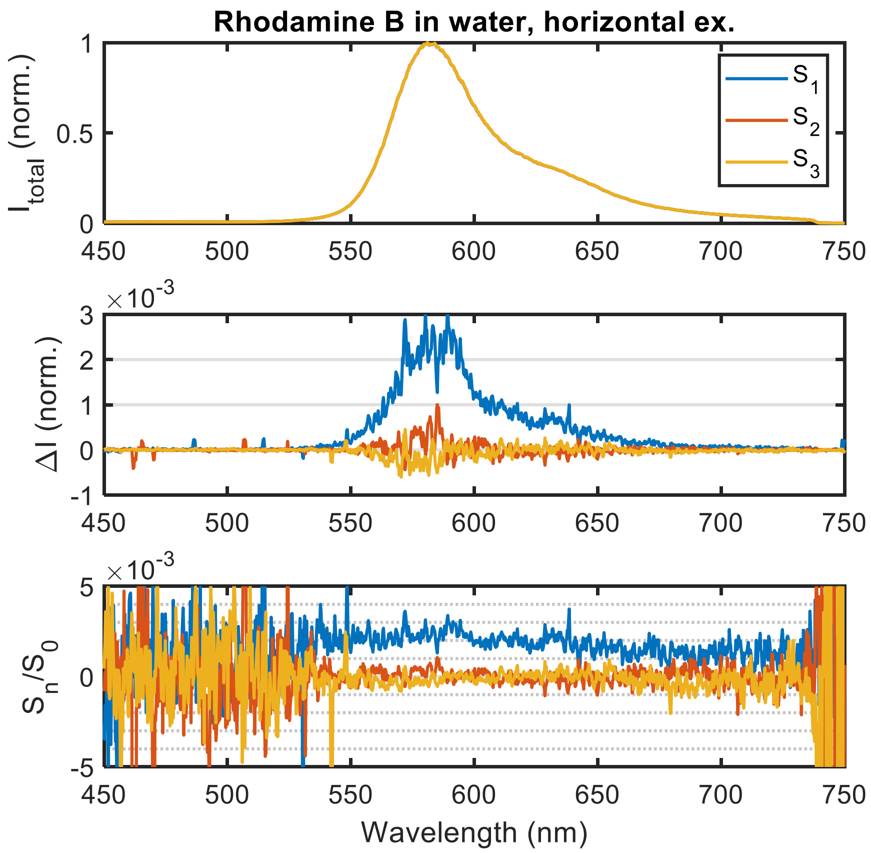

Figure S13: Full-Stokes measurement of rhodamine B in aqueous solution with horizontal excitation polarization displaying low-noise baselines (excitation 343 nm, 200 fs, 50 kHz). There is a small S_1 offset observed, which may be due to slight imperfections in the polarization state of the excitation beam or the excitation beam orientation slightly deviating from orthogonal w.r.t the collection axis.

Restructured and rewrote parts of SI Section 4.10 (formerly Section 4.4), adding details about error sources including baseline shifts and how these can be mitigated:

4.10: Beam Deviation and Waveplate Rotation

For the waveplates used (Thorlabs, QWP: SAQWP05M-700 and HWP: SAHWP05M-700) the manufacturer specifies a beam deviation of < 3 arcmin. We have found the extent of deviation to vary somewhat between individual waveplates. Though the deviation tolerances are quite small, rotating the waveplate causes the transmitted beam to trace a circle. This may cause noticeable effects at collection optics, with some dependence on experimental parameters like slit width, excitation geometry and so on. While good results may be achievable without addressing waveplate deviation explicitly in fortuitous cases, for reproducible performance across different measurements awareness of such effects is important.

For simplicity, we split the deviation effects into horizontal and vertical deviation, as illustrated in Figure S23. These predominantly affect different parts of detection, with horizontal deviation mainly an issue at the spectrograph entrance slit and vertical deviation mainly an issue at the CCD array.

Horizontal deviation may cause partial clipping of the beams going into both tracks, as the spectrograph in-coupling slit is small and vertically oriented. This will lead to a change in recorded intensity as the waveplate is rotated. We have found that this intensity change is not always fully cancelled out. We suspect this is because the assumption for error-cancelling is that any “time instability” error sources affect both channels in an identical fashion, which is not necessarily the case as the clipping at the slit happens after the polarization components are separated into two beams. The result is generally a baseline offset for dissymmetry measurements, for example, an achiral sample showing apparent CPL activity with g_{lum} on the order of 10^{-3} (see Figure S24 for rhodamine B). This effect can, however, be mitigated.

Vertical deviation, on the other hand, causes the pixels which collect light in the second measurement (waveplate orientation) to not be exactly the same pixels as in the first measurement. Due to this, the assumption for error-cancelling (that any “channel mismatch” error sources are constant over subsequent measurements) of pixel sensitivity variations is not met, resulting in fixed-pattern noise (*i.e.* consistent between measurements) approximately at the 10^{-2} level which does not improve on further data accumulation. This is illustrated in Figure S24, where it can be seen that noise is consistently present in the same pixels even in separate measurements with different horizontal pixel binning values. This can also be mitigated.

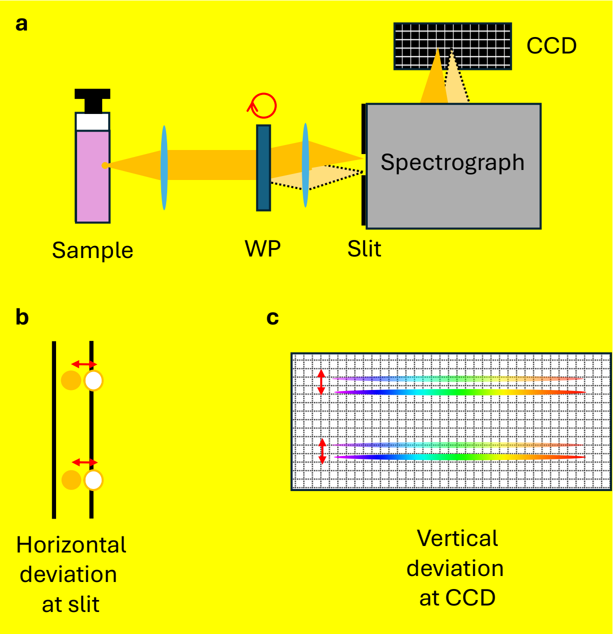

Figure S23: Simplified illustration of the effects of waveplate deviation, which causes the beam to slightly move upon waveplate rotation. At the slit, this may result in clipping of the beam, if horizontal movement occurs. At the CCD, this may result in different pixels collecting the signal, if vertical movement occurs, which will interfere with averaging out channel transmission characteristics.

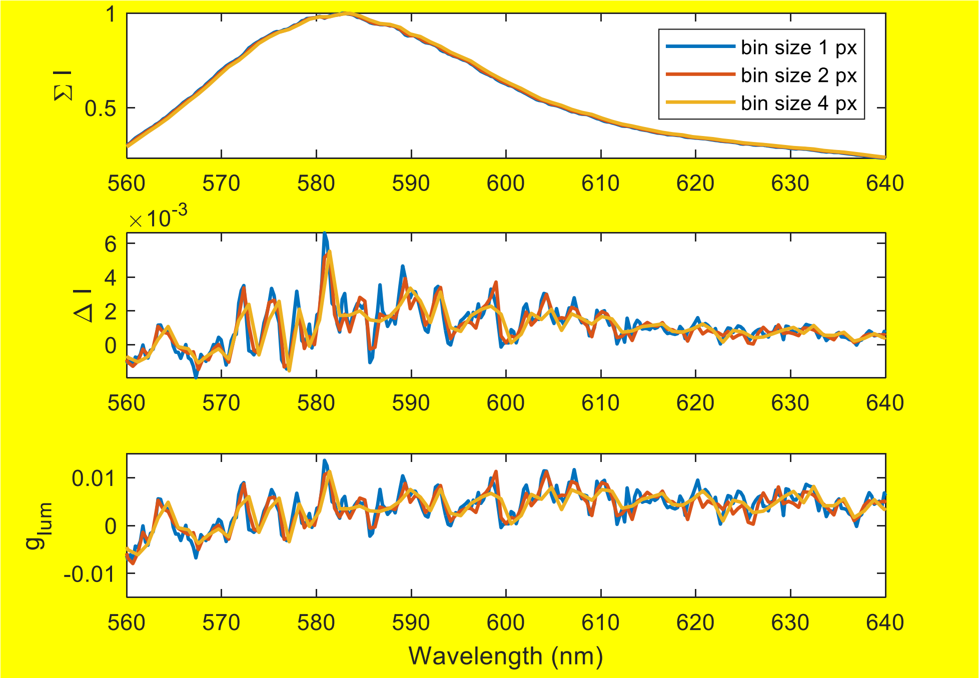

Figure S24: CPL measurement of rhodamine B in aqueous solution (excitation 515 nm, 200 fs, 50 kHz, horizontal polarization) with various horizontal pixel binning values. This data shows two effects of beam deviation during waveplate rotation. First, a baseline offset (CPL artifact) due to channel-dependent intensity variations from horizontal beam deviation. Second, a higher-than-expected non-random noise background arising from incomplete cancellation of fixed-pattern noise due to vertical beam deviation. Both effects can be mitigated, as explained in the text.

While the primary error-cancellation procedure calls for measuring over two waveplate orientations (out of 4/8 possible ones for the QWP/HWP, respectively), using further waveplate orientations (calculation described in SI section 2.1) can alleviate beam deviation effects. This is illustrated for horizontal deviation offsets in Figure S25. Depending on the waveplate angle pair chosen, the CPL offset changes sign but approximately retains its magnitude. We believe this to arise from the deviated beam tracing a circle over full rotation of the waveplate, causing a symmetrical effect on the channel intensities for the second pair of angles. Consequently, using data from all four QWP angles results in near-complete elimination of the baseline offset.

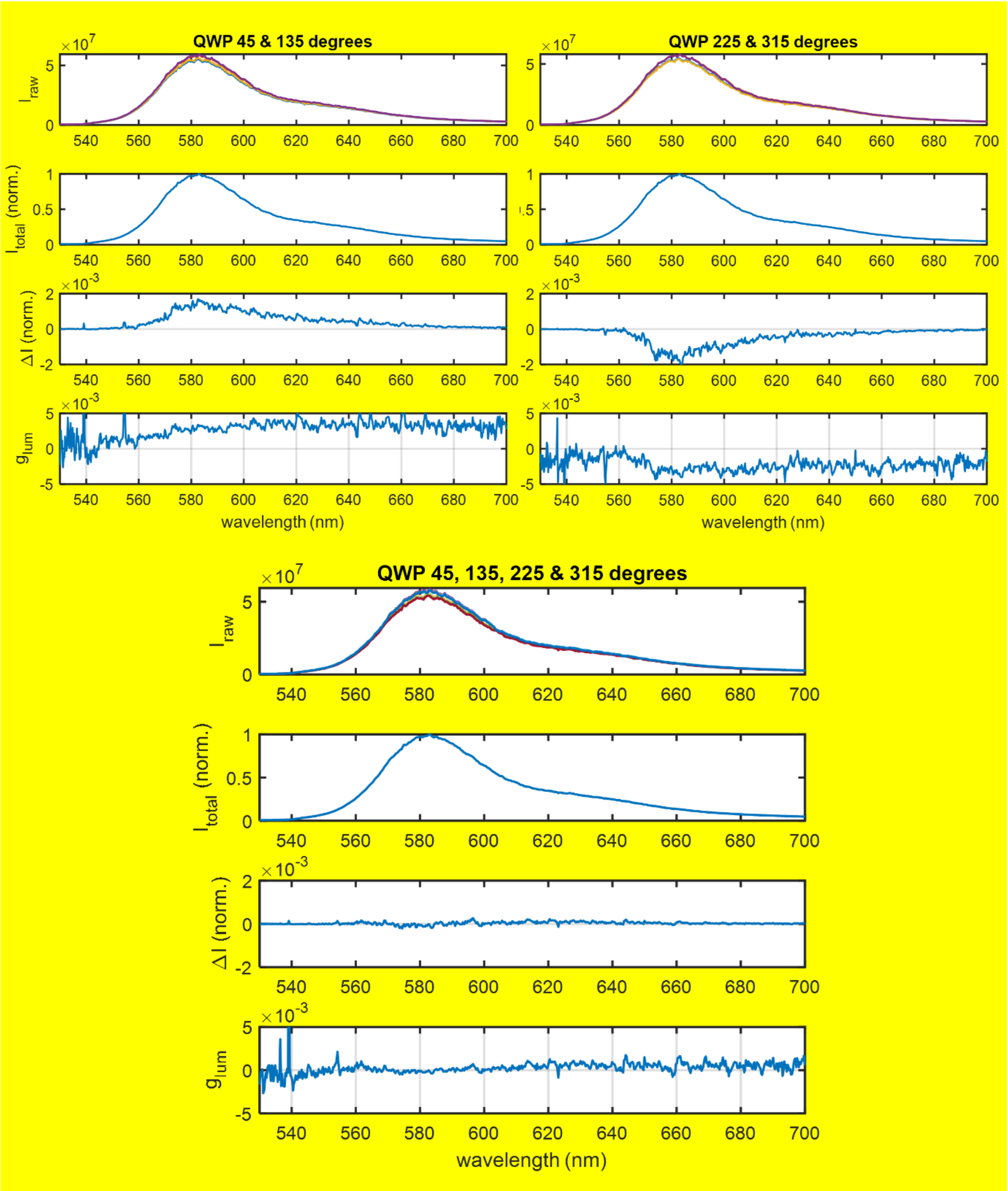

Figure S25: CPL measurement of rhodamine B in aqueous solution (excitation 515 nm, 200 fs, 50 kHz) using various quarter-wave plate (QWP) angle combinations. Due to horizontal beam deviation, a small baseline offset artifact is observed when using two QWP angles for error-

cancellation, with the sign depending on the angle pair chosen. Measuring over all four QWP angles cancels out this effect, resulting in a clean baseline.

Horizontal beam deflection can further be mitigated by using a 90º excitation-collection geometry with a slow excitation focusing lens, such that the excitation beam forms a line through the sample.

Besides using multiple waveplate orientations, vertical deviation effects can also be corrected for by the somewhat crude but effective method of steering the excitation beam vertically after QWP rotation such that the same pixels are used to collect signal in both measurements. This is relatively simple, as the detector has a live imaging readout making alignment straightforward. However, this relies on the sample being homogeneous (valid for solutions). Some amount of transmission change from beam steering is unavoidable, although this was found to work sufficiently well for high-accuracy measurements in our case.

Manual beam resteering is illustrated in Figure S26, and the result on measurement "noise" in Figure S27. For exactly the same measurement parameters, the apparent noise level is massively improved by manually resteering the beam after waveplate rotation. This is because the "noise" without beam resteering is not true statistical noise, but rather static imperfections in the error cancellation caused by changing the pixels over which data is collected.

While these approaches allow for control of deviation-induced effects sufficiently to perform high-accuracy polarization measurements, it would be preferable to eliminate waveplate deviation entirely in the future. Besides higher-spec waveplates, approaches such as deviation-compensating optical elements are a potential way to manage the effect directly at the cost of additional optical elements.

Besides mitigating beam deflection by optics, a possibly elegant and automatable approach to mitigate pixel sensitivity is the scanning multichannel approach. Here, instead of doing a single series of accumulations at a given grating position, a smaller number of accumulations is performed at multiple slightly offset grating positions (*54*). This approach has been successfully used to mitigate pixel noise in, for example, femtosecond stimulated Raman spectroscopy (*55*). As an added benefit, this will similarly smooth out any larger-scale sensitivity variations of the intensifier/detector, which normally have slightly lower sensitivity near the edge regions, without requiring a manual calibration file. However, transmission characteristics of optics will still contribute to spectral shape and require calibration to recover the original spectrum.

We thank the reviewer again for stimulating this extended discussion on potential instrument error sources and how they are mitigated which we believe has further improved the quality of this work.

Response Letter for Nature manuscript 2025-01-00882A

“Broadband transient full-Stokes luminescence spectroscopy”

Referee #1:

I have checked the revised version of manuscript, SI, and response letter. The authors have accurately addressed the issues pointed out by all referees. In detail, the authors clearly showed all experimental temperatures during measurements. According to the previous reports about temperature dependance of g_{lum} (e.g. Front. Chem., 2020, 8, 527), such a small temperature deviation (21 ± 0.3 ˚C) only cannot account for the drastic variation of glum values shown in Figure 2g and 2h. Therefore, it is proved that the current manuscript showed consistent conclusion also from the aspect of temperature dependence of g_{lum}. The proposed approach is original and extremely significant in this research field. The results shown in the current manuscript are high quality and the paper is also well organized. I consider that the manuscript is now ready to be published in Nature.

We thank the reviewer again for their kind comments and confidence in our work.

Referee #2:

Dear Authors, dear Editor,

I have commented in detail this paper in my previous report. Let me just add something on this revision.

I am glad to see that this article has undergone a revision, responding to all more relevant concerns that were raised. From my side, I believe that it can now be published essentially as it is (with some attention to my latter comments).

We would like to thank the reviewer again for their in-depth analysis in this and the previous review round, and for their support for our work. We address the additional comments below.

Concerning my own ones, there a point in the rebuttal, which seems to deserve further clarification. I agree that "multiple decay pathways for a single starting state does not in itself lead to multi-exponential behaviour" and very clearly: "multiple starting states" scenario is not the relevant one here. The fact is that here we can't appreciate it, due to limited resolution of the emission monochromator or to intrinsically unresolved lines, but the D0->F1 is *not* a single line, but rather a triplet of lines, potentially each with its own decay rate, hence the (potential) multiexponential decay of the convoluted line.

We thank the reviewer for the clarification. The part of our discussion in the last response letter pertaining to the case of a single starting state and multiple final states remains relevant. It would certainly be interesting to see how the dissymmetries of transitions to different sublevels differ, especially in a time-resolved manner. Yet, as pointed out by the reviewer, this is unlikely to be feasible as the line widths are greater than the sublevel separation, at least in the current setup but quite possibly even intrinsically, at least at room temperature, making such experiments difficult to realize in practice using our currently employed methodology.

I should also emphasize that the 90° excitation with H-polarized light in viscous media leads to much weaker emission spectra than with in-line excitation with unpolarized light. This said, I appreciate that the proposed setup uses an intrinsically LP-source and depolarizing it for in-line excitation would be a major complication and prone to error.

We thank the reviewer for the comment, and agree that this is a potentially useful consideration when considering suitable methodologies. SI Section 4.1 already contained a statement to this effect, and we have now added emphasis:

Further, emission intensity along an axis orthogonal to the excitation may be significantly reduced, especially in the limit of infinite viscosity and limited depolarization (*51*).

I would also like to add a small comment on the exchange with Referee 3 (comment 1), keeping in line with what I wrote just above.

When validating a new instrument or a new setup, comparing data collected with other instrumentation is essential, although it may represent some critical aspects. In the case of chiroptical spectroscopies and in particular of certain metal-centred CPL spectra, there is the very major problem of resolution. As well known in the literature, but clearly and recently

pointed out in 10.1021/acs.inorgchem.4c00417, spectra appearance and even quantitative data strongly depend on acquisition parameters and instrumental features. Any comparison must not overlook this aspect and one may even accept that the match is only fair, provided there is a good explanation.

I should also point out that there is no “sign inversion within individual transition” (this can occur only e.g. in the presence of CP-reabsorption). Here one would deal with multiple nearby transitions due to MJ-splittings.

We thank the reviewer for providing clarification on these points and fully agree.

I notice that the g-factor plots are practically zero when the emission (and CPL) vanish. In fact, it should be an undetermined form (0/0). The authors should explain how they took care of this.

We thank the reviewer for the comment. This is an interesting point so we will discuss it at some length.

We agree that, in principle, in spectral regions outside the sample luminescence the g-factor should be undetermined rather than zero. In such regions the measured g-factors do show increased noise (as shown *e.g.* in Figure S13, copied below). However, as the reviewer points out, the g-factor maintains a “finite” noise level about zero, which may appear unexpected given that the denominator in the g-factor calculation in this region should be zero.

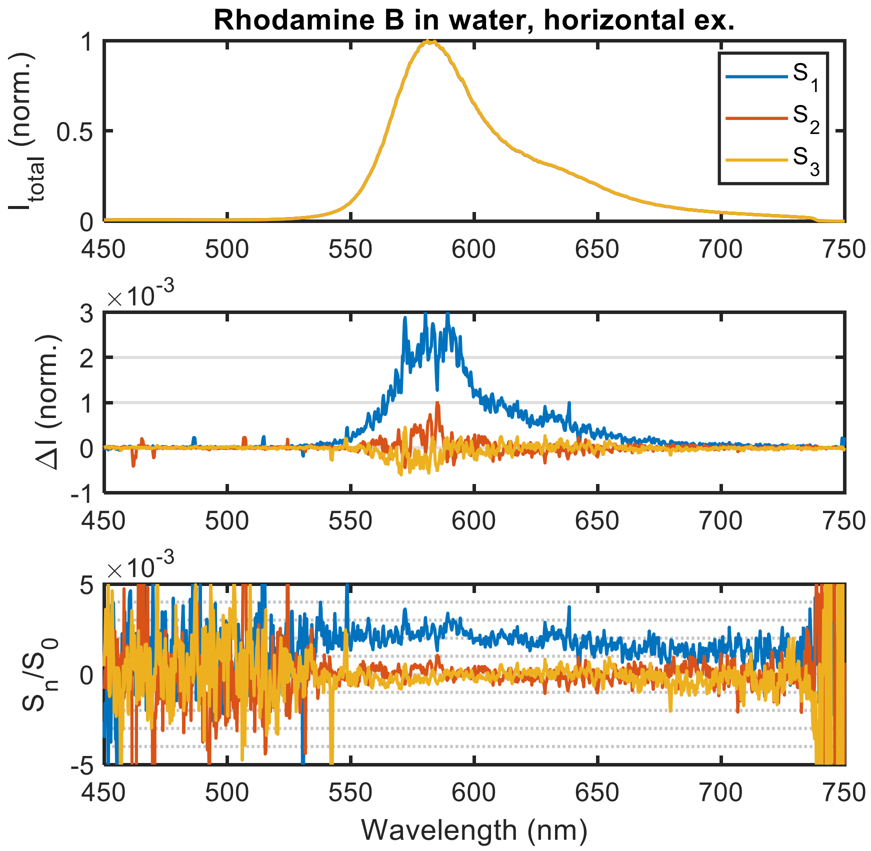

Our data processing is extremely simple (as described in the corresponding Methods subsection) and does not have anything to “force” a zero g-factor in any spectral region. As CCD detectors have a finite “dark” background (counts measured in the absence of light incident), our data is background corrected, with the background taken using exactly the same measurement parameters, only with the excitation source blocked.

A non-diverging g-factor in regions where sample luminescence is present is most straightforwardly explained by the presence of a small “background” signal which is only present when the excitation source is switched on. In this case, even in “dark” spectral regions the measured intensity is finite and therefore the g-factor, while random about zero, is not completely diverging.

We believe a likely cause for such a background is stray light within the spectrograph. This will in general cause a non-zero intensity reading in pixels located outside the horizontal spectral range of the true luminescence spectrum.

Some degree of stray light is inevitable even in relatively high-spec systems. While weak, such light might be expected to be present at intensities on the order of 10^{-4}-10^{-5} relative to the true signal peak (*e.g.* values mentioned in https://www.newport.com/t/stray-light-in-monochromators-and-spectrographs).

As collecting CPL spectra with high signal-to-noise ratios requires collecting counts at levels on the order of 10^{6}-10^{8}, even at these low stray light specifications it is expected that stray light is accumulated at measurable levels. Accordingly, it is not entirely surprising that the g-factors do not completely diverge even in regions outside the luminescence spectrum.

Systems based on monochromators (slit output) and especially multiple monochromators can, in principle, significantly reduce stray light. However, for the purposes of broadband detection this is not feasible. In general, stray light “correction” is extremely challenging as it tends to vary with measurement parameters, and efforts to eliminate stray light are already implemented by spectrograph manufacturers to a good standard.

Importantly, in our opinion, such stray light is unlikely to significantly impact real dissymmetry values measured. The impact on g-factors would be second-order small as both polarization components are very similarly affected by stray light. More explicitly, the difference in stray light levels between polarization components is proportional, with a small constant of proportionality, to the difference in intensity between polarization components, which itself is generally a small fraction of the overall intensity.

As far as we can conceive, stray light is only likely to impact g-factors in regions where the luminescence intensity is a small ($\sim 10^{-4}$) fraction of its peak value. Generally, most interest is in evaluating CPL activity in regions where significant luminescence is actually present. If such weak regions are to be focused on, the bright features can be blocked (and stray light reduced accordingly) by spectral filtering. In fact, this can also be achieved by time gating in appropriate cases and constitutes a major strength of our approach, as illustrated in Figures 2 and 3 of the main body where short-lived species are temporally separated from the main luminescence. Time gating has the added advantage of allowing separation of spectrally overlapping species with disparate steady-state intensities.

The fact that *e.g.* Figure S13 shows a non-diverging noise level centered about zero rather than a false CPL value implies that even in a weak luminescence region and a low-noise

measurement with many accumulations, stray light does not lead to false g-factors on levels measurable within signal-to-noise.

For all the reasons above, we believe that the presence of non-diverging g-factors in areas outside the luminescence spectrum for low-noise measurements with high total accumulations is expected given imperfections in optical components. While impractical to completely avoid in a real system, these do not constitute a significant concern for data validity or present a significant limitation of the method's applicability.

Finally, the authors recall potential contributions arising from linear dichroism/linear birefringence. This would apply in absorption, whereas here LD should be substituted by fluorescence anisotropy.

We thank the reviewer for their comment. In this particular instance we are slightly unsure about which part of the manuscript the comment refers to.

SI Section 4.2 discusses errors due to sample birefringence, but in this instance, it is circular birefringence which is being discussed, and this is probably not what the reviewer refers to.

The main body contains several references to linear anisotropy of luminescence and the errors associated (*e.g.* in the section "Measuring principle" on page 3 and "Polarization artifacts and relaxation in an achiral dye" on pages 12-13). Some discussion of these effects is also found in SI Section 4.1. All these instances are indeed the same concept and context referred to by the reviewer, *i.e.* artifacts arising from linearly polarized luminescence (a.k.a. fluorescence (linear) anisotropy), as opposed to LD (linear dichroism) in the absorbance.

In our work, we refer to such luminescence components as linear polarization (LP) components, to distinguish them from circular polarization (CP, or CPL for circularly polarized luminescence) components.

Since the manuscript contains multiple references to linearly polarized luminescence (abbreviated there as LP) and we make no references to linear dichroism (commonly abbreviated as LD), we believe there may have been a confusion between these abbreviations.

In any case, in the instances where linear polarization-induced artifacts are discussed in the manuscript, such artifacts are indeed explicitly identified as originating from linear anisotropy of emission rather than absorption, and abbreviated distinctly (LP for linearly polarized luminescence) from the standard abbreviation for absorption linear anisotropy (LD for linear dichroism).

To reduce potential confusion, we have explicitly added the word "luminescence" to the section defining the abbreviation LP on page 3 of the main body:

"Accordingly, linear polarization (LP) components in luminescence (a primary error source in CPL measurements[18]) can be quantified in addition to CPL."

We would like to thank the reviewer again for all the insightful discussions along the review process.
